# Lack of nAChR Activity Depresses Cochlear Maturation and Up-Regulates GABA System Components: Temporal Profiling of Gene Expression in α9 Null Mice

**DOI:** 10.1371/journal.pone.0009058

**Published:** 2010-02-04

**Authors:** Sevin Turcan, Donna K. Slonim, Douglas E. Vetter

**Affiliations:** 1 Department of Biomedical Engineering, Tufts University, Medford, Massachusetts, United States of America; 2 Department of Computer Science, Tufts University, Medford, Massachusetts, United States of America; 3 Department of Neuroscience, Tufts University School of Medicine, Boston, Massachusetts, United States of America; Vrije Universiteit Amsterdam, The Netherlands

## Abstract

**Background:**

It has previously been shown that deletion of *chrna9*, the gene encoding the α9 nicotinic acetylcholine receptor (nAChR) subunit, results in abnormal synaptic terminal structure. Additionally, all nAChR-mediated cochlear activity is lost, as characterized by a failure of the descending efferent system to suppress cochlear responses to sound. In an effort to characterize the molecular mechanisms underlying the structural and functional consequences following loss of α9 subunit expression, we performed whole-transcriptome gene expression analyses on cochleae of wild type and α9 knockout (α9^−/−^) mice during postnatal days spanning critical periods of synapse formation and maturation.

**Principal Findings:**

Data revealed that loss of α9 receptor subunit expression leads to an up-regulation of genes involved in synaptic transmission and ion channel activity. Unexpectedly, loss of α9 receptor subunit expression also resulted in an increased expression of genes encoding GABA receptor subunits and the GABA synthetic enzyme, glutamic acid decarboxylase. These data suggest the existence of a previously unrecognized association between the nicotinic cholinergic and GABAergic systems in the cochlea. Computational analyses have highlighted differential expression of several gene sets upon loss of nicotinic cholinergic activity in the cochlea. Time-series analysis of whole transcriptome patterns, represented as self-organizing maps, revealed a disparate pattern of gene expression between α9^−/−^ and wild type cochleae at the onset of hearing (P13), with knockout samples resembling immature postnatal ages.

**Conclusions:**

We have taken a systems biology approach to provide insight into molecular programs influenced by the loss of nicotinic receptor-based cholinergic activity in the cochlea and to identify candidate genes that may be involved in nicotinic cholinergic synapse formation, stabilization or function within the inner ear. Additionally, our data indicate a change in the GABAergic system upon loss of α9 nicotinic receptor subunit within the cochlea.

## Introduction

In the mature mammalian cochlea, auditory stimuli are transduced into receptor potentials and transmitted to the brain via cochlear inner hair cells (IHCs) and the ganglion cells contacting them. However, unlike afferent activity of other major sensory systems, sound transmission from the cochlea is directly controlled by a sound-evoked feedback loop embodied by the olivocochlear (OC) efferent system fibers. The murine OC system is classically divided into lateral and medial olivocochlear systems, each with their own origin and hair cell population as a target. The medial OC (MOC) efferents originate in the medial portion of the superior olivary complex and project axons that form nicotinic cholinergic synapses at the base of outer hair cells (OHCs) [1]–[3]. The acetylcholine sensitivity of this synapse is generated by a heteromeric nicotinic receptor composed of two subunits, α9 and α10 [4], [5], arranged in a pentameric structure [6]. The activation of receptors composed of α9α10 subunits leads to an increase in intracellular Ca^2+^ levels [7] and subsequent opening of calcium-activated K^+^ channels (SK2 channels) leading to hyperpolarization of OHCs [8]. Overall, the activation of MOC efferents inhibits mechanical amplification of sounds, thus reducing cochlear sensitivity [9], which has also been shown to provide protection against acoustic trauma [10]–[12]. However, a recent study of postnatal development of the cochlea has provided clues that loss of nAChR activity has consequences to the expression of a wide array of proteins potentially involved in synapse maturation [13].

In rodents, MOC efferents follow a dynamic pattern of innervation during the first two weeks of postnatal life. During early postnatal development, MOC efferent axons innervate IHCs. However, by the end of the second postnatal week, two major changes in the cochlea have occurred. Structurally, most of the early direct efferent synaptic connections to the IHCs are pruned away from directly contacting the IHCs, and re-targeted to OHCs, where they form mature synapses that are maintained through adulthood [14], [15]. Functionally, it is near the end of the second postnatal week that mice begin responding to sound with more mature auditory nerve activation thresholds [16]. Constitutive genetic ablation of *chrna9*, the gene encoding the nAChR α9 subunit (α9^−/−^) results in abnormal synaptic morphology that includes hypertrophy of the efferent synaptic terminals at the base of outer hair cells beginning from the earliest stages of postnatal development [17]. Physiologically, adult α9^−/−^ mice fail to show classic OC responses, characterized by an inability of OC system to modulate OHC activity [17]. During the second postnatal week, α9^−/−^ mice possess altered expression of presynaptic active zone proteins, trans-synaptic cell adhesion proteins, and CREB signaling proteins, while also having hypertrophied efferent synaptic terminal structure similar to that described in adults [13]. Contrary to α9^−/−^ mice, a gain-of-function point mutation in the α9 subunit results in hyperinnervation of OHCs [13]. Additionally, loss of SK2 channel expression, which does not alter expression of either α9 or α10 transcript expression, results in a degenerating efferent innervation that begins shortly after initial synaptic contact with the OHCs [18]. Together, these mouse models suggest that activity via the hair cell nAChR is capable of altering gene expression of numerous genes involved in synapse formation and efferent function, many of which remain unknown.

Currently, the molecular mechanisms underlying formation and maintenance of cholinergic synapses beyond those of the neuromuscular junction (NMJ) remain largely unidentified. Revealing the intrinsic program of gene expression that accompanies nicotinic cholinergic synapse development in the cochlea is a key step in identifying molecular targets and pathways involved in fast nicotinic transmission and that may be involved in the inner ear's protective mechanisms against moderately intense sound and human disease states such as tinnitus. In this study, whole-genome microarrays were utilized to conduct a comparative analysis of gene expression changes in wild type and α9^−/−^ mice spanning the period of active efferent synaptogenesis and stabilization in the postnatal cochlea. We provide insights into the molecular mechanisms and consequences affected by loss of nicotinic cholinergic activity throughout cochlear development. Chief among our results is the discovery of several functional modules involved in synaptic transmission that are differentially represented in the α9^−/−^ cochlea compared to age-matched wild type mice. Moreover, the data indicate that the overall cochlear gene expression profile of α9^−/−^ mice is distinctly different from that of wild type mice at the onset of hearing. Interestingly, at the onset of hearing, knockout samples are represented by an expression profile more characteristic of immature postnatal ages. Furthermore, loss of α9 subunit results in increased gene expression of GABAergic receptor subunits and glutamic acid decarboxylase (GAD), the synthetic enzyme for GABA. Using immunoprecipitation, immunohistochemistry and qPCR methods, we validate the microarray data, including altered GABAergic receptor expression in mice lacking the α9 subunit. Our data suggest an association between the well-studied nicotinic cholinergic OC system and the less thoroughly understood GABAergic OC system of the murine cochlea.

## Results

We used an Affymetrix gene chip approach to assess gene expression changes brought about as a consequence of constitutive loss of the *chrna9* gene, encoding the α9 nAChR subunit, in an attempt to better define the role of this gene and nAChR activity in cochlear processing. Briefly, our gene expression analysis took the form of a three-part approach. First, we defined individual genes that have undergone differential expression across age and genotype. This was followed by an analysis of our data at the level of gene sets (using gene set enrichment and bicluster analyses) in an attempt to discern potentially novel gene interactions that are impacted by loss of nAChR activity. Finally, this was followed by a dynamic analysis of the whole transcriptome (using Gene Expression Dynamics Inspector and cluster analysis tools) to decrease the high dimensionality of the data and annotate coordinated genome wide responses that occur over age and by genotype. A flowchart of microarray analysis methods is outlined in Fig. S1.

### Overview of Transcriptome Changes Induced by the Loss of α9 Nicotinic Receptor Subunit

**Figure 1:**
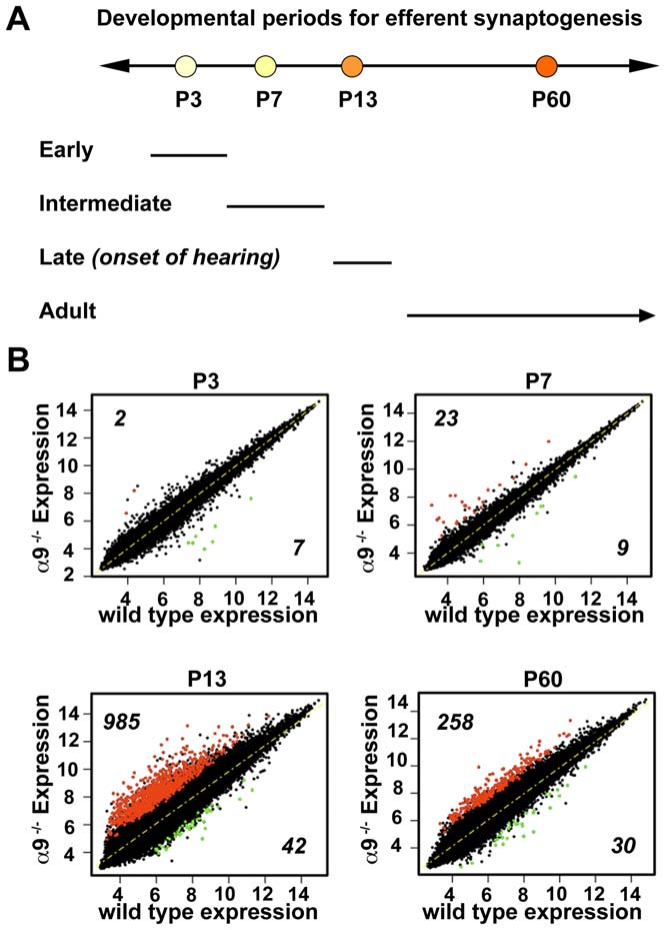
Overview of differentially expressed genes in the α9^−/−^ compared to wild type. (**A**) The ages indicate the postnatal (P) days at which cochlear RNA samples were prepared. Efferent innervation in the cochlea peaks around P7 when most of the efferent axons are contacting inner hair cells. Between P7 and P13, the axons are pruned and retargeted to outer hair cells. P13 also marks the initial phase of auditory function stabilization in rodents. Microarray analyses were based on comparative analyses of α9^−/−^ and wild type at P3, P7, P13 and P60. (**B**) Comparison of expression levels of all probes between α9^−/−^ samples and wild type controls at each age. Normalized gene expression data are plotted on a logarithmic scale (log_2_), and genes changing in the α9^−/−^ by at least 1.5 fold and differentially expressed using the *limma* package in R (FDR q-value <5%) are highlighted for each age. Red dots correspond to significant up-regulation and green to significant down-regulation in the knockout animals compared to wild type controls. Numbers of up- and down-regulated genes are also noted in the plots.

Whole-genome microarrays were used to sample the changes in the dynamic period of synapse formation, stabilization and maturation in the mouse cochlea upon loss of α9 nicotinic subunit. Cochlear tissue was collected for RNA isolation at a range of postnatal ages chosen to represent critical periods during the time course of efferent synaptogenesis. Specifically, cochleae were collected from α9^−/−^ and age-matched wild type animals during pre-hearing (P3, P7), hearing-onset (P13) and mature (P60) periods (Fig. 1A). The microarray data along with design parameters have been deposited in NCBI Gene Expression Omnibus Database (GEO; http://www.ncbi.nlm.nih.gov/geo/) under the accession number GSE18567.

**Figure 2:**
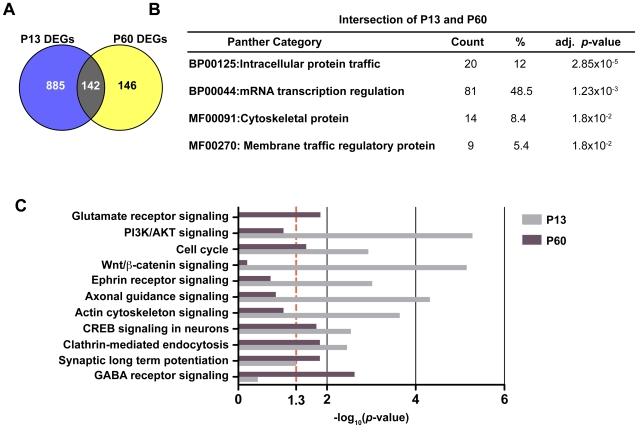
Functional categories of genes significantly over-represented in α9^−/−^. (**A**) Venn diagram depicting the overlap among differentially expressed genes (DEGs) between P13 and P60. The intersection of differentially expressed genes between wild type and α9^−/−^ consists of 142 genes. (B) Over-represented classes among these 142 genes are shown (DAVID, BH-adjusted *p*-value <0.05). (C) Comparative enrichment of functional categories (Ingenuity database; −log_10_ (enrichment *p*-value)) between P13 and P60 are indicated; larger values indicate higher significance. The dotted line at 1.3 corresponds to *p*-values of 0.05 on this scale.

Cochleae were pooled into three separate samples representing biological triplicates for each age and genotype. The mean Pearson correlation between biological triplicates for all samples were higher than 0.97. Raw data were quantile-normalized and filtered to remove probe sets with low variance across samples. Differential expression was calculated separately for each age using a moderated *t*-test implemented in the limma BioConductor library [19]. The *p*-values were adjusted for multiple testing using the false discovery rate (FDR) approach [20]. Transcripts were selected as significantly altered compared to wild type when the absolute average log_2_ ratio calculated from biological triplicates per time point was greater than 1.5 and the difference between the mean expression for the wild type and knockout animals was significant at a FDR-corrected *p*-value <0.05. The expression levels of gene transcripts were compared between wild type and α9^−/−^ mice at each age, yielding a total of 1,356 unique probes that were significantly modulated in the α9^−/−^ over any of the sampled periods (the complete list of significantly changing genes is reported in Table S1) (Fig. 1B). The total number of differentially expressed genes between wild type and knockout mice was the greatest at P13, the onset of hearing, comprising 75.7% of the total of differentially expressed genes. Furthermore, approximately 50% of those genes differentially expressed at P60 were already differentially expressed at P13 between genotypes, thus showing a convergence of gene expression across ages (Table S2) (Fig. 2A).

Fewer genes showed differential expression at P3 and P7, and analysis in DAVID showed no significantly over-represented ontological terms. Notably, at P3, down-regulated genes included molecules implicated in neuronal activities (*Msi2, Ndel1*) and up-regulated genes included molecules associated with axonal guidance (*Epha3*). At P7, several molecules involved in nucleic acid binding (e.g., *Trove2, Eif2s1, Nudt1, Ddx3y*) were differentially expressed.

Specifically, there were 142 genes that were in the overlap between P13 and P60 (Fig. 2A). Next, we examined whether the 142 common genes affected by the loss of α9 at P13 and P60 corresponded to specific biological functions. Towards this end, we used DAVID (Database for Annotation, Visualization and Integrated Discovery) [21] and Ingenuity Pathway Analysis (www.ingenuity.com) to group differentially expressed genes according to their respective biological and/or molecular processes at an adjusted *p*-value <0.05. The common differentially expressed genes at P13 and P60 were binned into several over-represented PANTHER categories [22] (BH adjusted *p*-val <0.05) that included intracellular protein traffic, mRNA transcription regulation, and processes involving cytoskeletal proteins (Fig. 2B).

At P13 and P60, certain biological processes, such as CREB signaling, clathrin-mediated endocytosis, and cell cycle were implicated at both time periods but by different genes, whereas others, such as ion channel signaling (glutamate and GABA receptors), were differentially expressed at one age or the other. Conversely, inhibitory, and to a lesser extent, excitatory neurotransmission is over-represented in α9^−/−^ at P60 compared to P13. CREB signaling was over-represented at both ages. This likely results from alterations in intracellular calcium levels due to the loss of a functional α9α10 nAChR, as evidenced in other systems where nicotinic signaling has been shown to activate CREB signaling pathways by altering intracellular calcium concentrations [23], [24].

**Figure 3:**
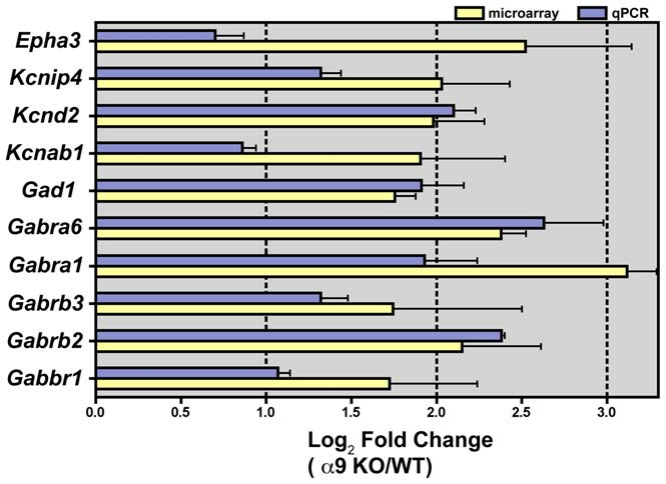
Confirmation of selected genes at the transcript level. (**A**) Log_2_ fold changes in expression levels of selected transcripts measured by qPCR are plotted alongside their fold changes obtained by microarray analysis. For the ten selected genes, expression values from microarray and qPCR showed similar relative changes (*p*<0.05, two-tailed *t*-test). Error bars indicate SEM.

Many gene sets involved in neuronal processes were up-regulated in the α9^−/−^ animals. At P13, these included ephrin receptor signaling, axonal guidance signaling, Wnt/β-catenin signaling, neuregulin signaling, as well as PI3K/AKT pathway (Fig. 2C). At P60, up-regulated genes included those encoding voltage-gated sodium channels (*Scn2a, Scn9a*), potassium channels and transporters (*Kcnd3, Kcnd2, Kcnab1, Kcnip4*), excitatory (*Gria4, Gria1*) and inhibitory neurotransmission (*Gabrb3, Gabrb2, Gabra6, Gabra1, Gabbr1 and Gad1*). qPCR analysis confirmed the up-regulation of several GABA receptor subunits and potassium channels in the α9^−/−^ animals (Fig. 3).

### Effect of Loss of α9^−/−^ on GABA Receptor Subunits

Because little is known of the function of the GABAergic system in cochlear processing, we subsequently analyzed the protein expression of GABA receptor subunits in adult (P60) animals to verify that gene expression changes led to alterations in protein expression. We selected GABA_A_α1 *(Gabra1)* and GABA_A_β2 *(Gabrb2)* for further examination. These two subunits exhibited a high fold change in α9^−/−^ mice in both microarray and qPCR experiments at P60 and are widely expressed in the central nervous system (CNS). GABA_A_ receptor subunit α1 is the most abundant of the GABA_A_ subunits and is often co-localized with β2 and γ2 subunits [25].

**Figure 4:**
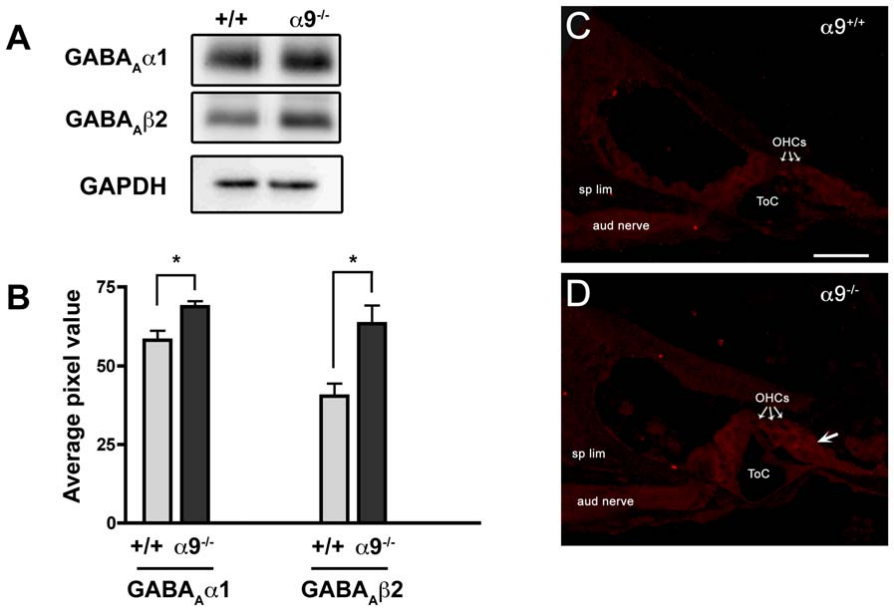
Quantitative protein expression analysis of select GABA receptor subunits and cellular localization of changes to GABA_A_β2 expression. (**A**) Protein expression of GABA_A_ subunits was assessed by immunoprecipitation (IP) to confirm changes observed at a transcriptional level. 200 µg cochlear lysates from wild type and α9^−/−^ were subjected to IP with antibodies against GABA_A_α1 and GABA_A_β2 subunits. The immunoprecipitates were analysed by western blotting with anti-GABA_A_α1 or anti- GABA_A_β2. A separate western blot was run in parallel using the same lysates used for IP experiments for wild type and α9^−/−^ samples. A representative western blot including GAPDH loading control is shown. (**B**) Quantitative western blot analyses for GABA_A_α1 and GABA_A_β2 are shown. Average pixel values averaged over biological triplicates from IP experiments are indicated for wild type and α9^−/−^. GABA_A_α1 protein expression increased by 18% in α9^−/−^ (*p* = 0.041) and GABA_A_β2 expression increased by 58% in α9^−/−^ (*p* = 0.029) when compared to wild type. Error bars indicate SEM, and a significant change is denoted with an asterisk (two-tailed *t*-test). (**C**) In adult wild type ears, GABA_A_β2 immunostaining is observed in the inner and outer hair cells. (**D**) In α9^−/−^ OHCs, GABA_A_β2 immunolabeling is particularly dense in the apical regions, as well as at the synaptic pole. The small arrows indicate outer hair cells and the larger arrow in (D) indicates the border between outer hair cells and Deiters' cells. *Sp lim*, spiral limbus; *ToC*, tunnel of Corti; *OHC*, outer hair cell. Scale bar, 25 µm.

Immunoprecipitation and western blotting indicated that ablation of the α9 gene increased expression levels of GABA_A_β2 (58%, *p*-value = 0.0287) and GABA_A_α1 (18%, *p*-value = 0.0406) (Fig. 4A–B). Using antibodies to the GABA_A_β2 subunit, we also performed immunohistochemical labeling to investigate the cellular source(s) of the altered GABA_A_β2 expression. Wild type and α9^−/−^ tissue sections were processed simultaneously and resultant labeling was examined by confocal microscopy, ensuring that the laser intensity and camera gain were held constant. Background, assessed along the auditory nerve and spiral limbus, was similar between wild type and α9^−/−^ mice. Confocal-based immunofluorescence labeling for GABA_A_β2 was localized specifically to the organ of Corti, occuring diffusely in the IHC and OHC cytoplasm and at the base of OHCs (Fig. 4C–D). In α9^−/−^ mice, GABA_A_β2 immunostain intensity in the sensory hair cells appeared higher than that of the age matched wild-type mice. Deiters cells, which reside below the OHCs and support their physical link to the basilar membrane, also appeared moderately more intensely labeled than in wild type. Although qualitative in nature, these results further support the finding that GABA_A_ receptor expression is increased in the α9^−/−^ mice, and that this increase occurs in the same cells previously shown to express GABA receptors under normal conditions [26].

### Gene Set Enrichment Analysis Shows Differential Expression of Several Signaling Pathways

Methods designed to reveal expression changes at the level of the single gene are powerful, but depend on that change being high with low variance across samples. As a complement to an analysis at the single gene level, it is possible to investigate whether specific pathways or processes represented by sets of genes are differentially expressed between samples. These sets of genes can elucidate higher order systems level cellular responses to changing conditions. Gene Set Enrichment Analysis (GSEA), a statistical tool that assesses differential expression of *a priori* defined sets, was employed. GSEA can be used to identify subtle but consistent pathway changes in microarray data when analysis at the individual transcript level cannot detect statistically significant variation. For example, this approach has been used to define coordinate gene expression changes that are subtle at the level of the individual gene, and yet highly relevant to diabetes [27]. The GSEA method calculates an enrichment measure called the enrichment score (ES), which is a Kolmogorov-Smirnov running sum, and a normalized enrichment score (NES) that accounts for the size of the gene sets. The magnitude of ES reflects the degree of enrichment for a given gene set, and the sign of ES indicates the correlation of gene expression with a particular phenotype. For our analyses, a negative ES indicates that the molecules in a given gene set are down-regulated in the α9^−/−^ mice, and a positive ES indicates up-regulation in the knockout animals compared to wild type.

**Figure 5:**
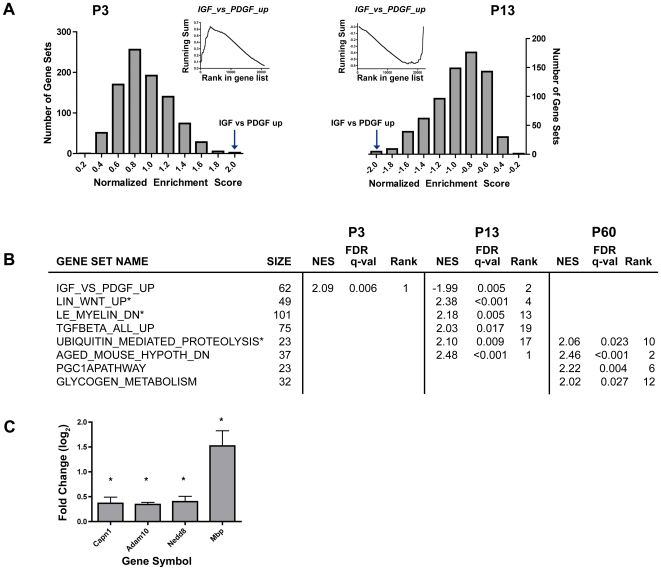
Gene set enrichment analysis of α9^−/−^ and wild type. (**A**) GSEA analysis of IGF1_vs_PDGF gene set. The inset depicts the running sum for the IGF1_vs_PDGF gene set at each gene in the rank-ordered list comparing α9^−/−^ and wild type at P60. The enrichment score is the maximum (up-regulated molecules) or minimum (down-regulated molecules) deviation from zero. The main plot shows the frequency distribution of normalized enrichment scores for all 1,390 gene sets. The arrow points to the bin that includes the IGF1_vs_PDGF gene set. (**B**) Several significantly differentially expressed gene sets with particular roles in synapse development or function are shown at P3, P13 and P60 (FDR q-value <0.05). Rank indicates the position of the gene set when compared to all others (a total of 1,390). (**C**) Log_2_ fold changes from qPCR validations are shown for four genes that were selected from three gene sets: *Capn1* (LIN_WNT_UP), *Adam10* and *Mbp* (LE_MYELIN_DN) and *Nedd8* (UBIQUITIN_MEDIATED_PROTEOLYSIS). A significant change is denoted with an asterisk (*p*<0.05, two-tailed *t*-test).

Sets of genes in the α9^−/−^ data were compared to wild type controls at specific developmental time points using GSEA. We considered 1,892 gene sets downloaded from the molecular signature database (MSigDB collection c2; Broad Institute, MIT). After filtering for gene set size (min = 15, max = 500), a total of 1,390 curated gene sets were obtained. One of the top enriched gene sets at P3 and P13 was the IGF-1 gene set, which corresponds to molecules differentially induced by IGF1 (when compared to PDGF) in myoblasts. Interestingly, the IGF1 gene set showed significant positive enrichment at P3 and negative enrichment at P13, suggesting a possible compensatory role at P3 upon loss of the α9 subunit (Fig. 5A). Other curated gene sets were preferentially enriched at either P13 or P60. At P13, the top enriched gene sets included myelination and several that are activated during synapse formation such as Wnt and TGF-β signaling, all significantly enriched at FDR q-val <0.05. The positive enrichment of these early development pathways in the α9^−/−^ animals at P13, at a time when the auditory function should become stabilized, may contribute to the formation of abnormal synaptic structures in α9^−/−^ adult cochleae since effectors of both Wnt and TGF-β pathways mediate cross talk between pre- and postsynaptic sites [28], [29]. However, at P60, modules related to energy metabolism such as PGC-1α pathway and glycogen metabolism were ranked at the top of enriched gene sets. Several gene sets were over-represented in the α9^−/−^ mice at both P13 and P60. For example, the *aged mouse cortex dn* set, which includes molecules that are down-regulated in the aging cortex, was up-regulated in the α9^−/−^ mice at both ages. Genes in this set include key genes involved in neuronal structure and signaling, such as Dynactin 1 (*Dctn1*) and Apolipoprotein E (*Apoe*). Additionally, ubiquitin-mediated proteolysis was significantly enriched at both P13 and P60, but was ranked in a different order (Fig. 5B). The misregulation of ubiquitin-mediated proteolysis parallels the over-representation of the CREB pathway at both ages. Additionally, we selected four genes in three of the GSEA categories for validation by qPCR at P13 (Fig. 5C). Out of four genes, *Mbp* was highly upregulated in α9^−/−^ mice and three (*Capn1*, *Adam10*, *Nedd8*) showed subtle yet statistically significant over-expression in α9^−/−^ mice.

### Neuronal Gene Set: A Subset of Neuronal Genes Differentially Expressed in the α9^−/−^

In addition to publicly available gene sets that span biological processes in a wide variety of tissues and experimental conditions, gene sets specifically enriched in neuronal tissues might also provide insight into the mechanisms directing the observed abnormal synaptic phenotype in the α9^−/−^ mice. Clustering methods have long been used to identify sets of co-regulated genes [30], [31]. However, these approaches have the potential to miss interesting patterns since they compute similarity over all experimental samples. Functionally related genes may be co-expressed only in a subset of conditions, and such gene sets would be missed by traditional clustering methods. Accordingly, biclustering methods [32]–[35], which cluster genes and conditions simultaneously, can be used to uncover sets of genes with coherent expression over a subset of samples. Biclustering has successfully been applied to microarray gene expression data from various systems to uncover abnormally expressed functional modules [36], [37].

We sought to identify biclusters that show meaningful patterns of coherent expression across data sets from different neuronal tissues and to test the hypothesis that these gene sets represent pathways generally relevant to neuronal function. We therefore compiled a collection of neuronal gene expression data to find biclusters that show coherent expression patterns over a subset of relevant samples. Specifically, single-channel gene expression data from three separate studies (GSE9803, GSE4040 and GSE4034) were downloaded from Gene Expression Omnibus (NCBI). All of the datasets were from mouse brain tissue samples hybridized to Affymetrix Mouse 430.2 chips. Twelve samples were from hippocampus, 9 from amygdala and 5 from striatum. Only data from wild type adult animals were considered, and data from specific mutants or treatments were excluded. The raw data were quantile-normalized as a group and filtered to remove probes with low expression variance across studies. Biclustering was performed on the resulting 22,550 probes and 29 samples using the Iterative Search Algorithm (ISA) as described [38] and as implemented by the BiCAT toolbox [39]. The ISA algorithm identified 33 “neuronal biclusters” across multiple brain tissues. The GSEA statistical framework was used as previously described above to evaluate differential expression of the biclusters on our whole-genome microarray data comparing wild type and α9^−/−^ cochlear samples (Table S3). We found 10 biclusters at P60, 17 biclusters at P13, 9 biclusters at P7 and 13 biclusters at P3 that showed significant differential expression in our data (FDR q-value <0.05).

**Figure 6:**
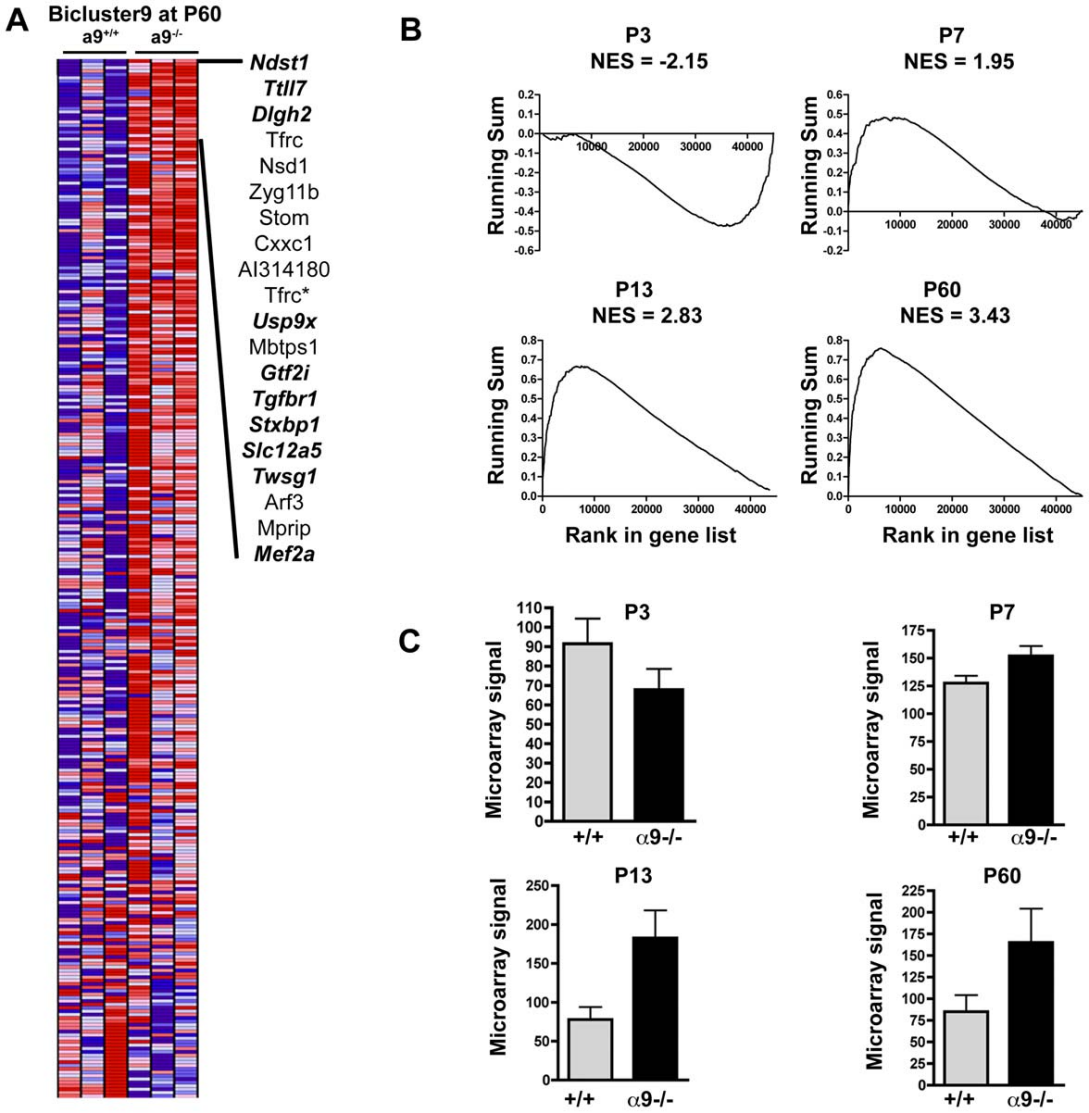
Gene set enrichment analysis of *bicluster9*. (**A**) All the genes included in bicluster9 are represented as a heatmap ordered by their ranking in the gene list comparing the expression between wild type and α9^−/−^ at P60. The top 20 genes that contribute the most to the enrichment score are shown. An asterisk next to the gene name indicates that it is repeated twice because of probe mappings. Italicized genes in bold have previously known functional roles in the nervous system. Red indicates high expression; blue indicates low expression. (**B**) The running sum enrichment scores for bicluster9 are shown for each age. *NES*, normalized enrichment score. (**C**) Leading edge subset analysis for bicluster9 at each age. Normalized microarray signal values for wild type and α9^−/−^ animals are averaged over all genes in the leading edge subset at each time point. Error bars indicate SEM.

Of the 33 biclusters, we selected bicluster9 (containing 306 probe sets) for further analysis because GSEA analysis ranked it as the most significantly differentially expressed bicluster between α9^−/−^ and wild type samples at P60, and because it displayed significant differential expression at all developmental ages. To interpret the biological importance of this bicluster, the PANTHER database was used to define significantly enriched functional categories among the 306 probe sets. Bicluster9 showed over-representation of several PANTHER categories known to be involved in neuronal processes, such as neurogenesis, SNARE protein-related processes, and expression of actin binding cytoskeletal proteins. Gene-by-gene inspection of the bicluster's 20 most differentially-expressed genes (according to GSEA) at P60 indicated that while a fraction of the genes have previously assigned roles in neuronal and synaptic development, notably *Mef2a*, *Dlgh2 (PSD93)* and *Stxbp1*, many have not previously been implicated in neural processes (Fig. 6A). Our results suggest a possible novel role for these genes in α9-mediated activity in the cochlea.

Bicluster9 also showed significant differential expression at all sampled postnatal ages. Of interest, this bicluster was significantly down-regulated in the knockout animals at P3, and up-regulated for the subsequent ages, suggesting its possible involvement in activity-induced changes (Fig. 6B). To quantitatively determine which members of bicluster9 contribute the most to ES, we extracted the core members that account for the enrichment score from the “leading edge” subsets [40]. The leading edge subset includes all genes from the set that increase the magnitude of that set's enrichment score; these are generally the most strongly up- or down-regulated genes in the gene set. The results indicated that at P3, 118 genes (out of 306) contribute to the enrichment score. This number decreased slightly at P7 (98), but increased at P13 (153) and at P60 (201) (Table S4). The average difference in normalized microarray signals of the leading-edge genes in bicluster9 between wild type and α9^−/−^ mice was also greatest at P13 and P60 (Fig. 6C).

We selected two genes from bicluster9 for validation by qPCR at P13. As described in the GSEA validation, *Capn1* (which was also a member of bicluster9) showed subtle yet statistically significant over-expression in α9^−/−^ mice (*p*<0.05, Fig. 5C). On the other hand, qPCR analysis of *Stom* expression revealed a slight increase in the knockout, but did not meet our threshold for significance (data not shown).

### Cluster Analysis of Global Expression Patterns

**Figure 7:**
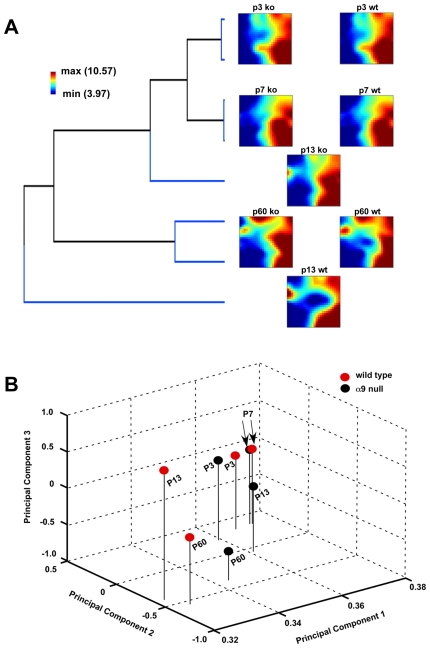
Comparison of dynamic gene expression levels of wild type and α9^−/−^. (**A**) Hierarchical clustering of centroids indicates similarity between genotypes and across ages. Mosaic patterns are pseudo-colored SOMs of the 650 metagene profiles from developing wild type and α9^−/−^ cochleae. Each tile contains genes with similar expression patterns and the spatial location of each tile is preserved across maps. Tile colors indicate the expression level of the centroids, with red representing high expression and blue representing low expression. (**B**) Principal component analysis (PCA) comparing SOM centroids. Each sample is projected onto a three dimensional grid comprised of the first three (strongest) principal components. PCA indicates the differences between wild type and α9^−/−^ cochlea at P13 and P60.

Following the single transcript and gene set level analyses, we assessed the dynamic transcriptome from a systems-level point of view. We reduced the dimensionality of the entire transcriptome using the gene expression dynamics inspector (GEDI) program [41]. GEDI enables the visualization of time series data by mapping similar temporal profiles into a mosaic pattern of genes using self-organizing maps (SOMs). Specifically, the gene expression profiles of 4 time points (averaged over biological triplicates) were visualized using 4 SOMs per genotype. In our time-series analysis, each SOM represents a mosaic of 26×25 tiles containing 650 clusters (Fig. 7A, Video S1, Video S2). The address of each tile is exactly repeated in each mosaic, and thus each address contains the same genes, allowing for direct comparison between SOMs of different ages.

We further subjected SOM clusters to a second level of clustering. Specifically, the centroids for each cluster in the SOM mosaic were hierarchically clustered using average linkage with Pearson correlation as the distance metric (Fig. 7A). Clustering of the centroids revealed the underlying similarities in gene expression between genotypes across ages. Since dendrogram structure can depend on the specific aggregation method chosen, the stability of the hierarchical clusters was evaluated by varying the methods used to calculate the dissimilarity matrix and to build dendrograms [42]. Seven aggregation methods were used to separately build the dendrogram (Ward, Mcquitty, centroid, median, single, average or complete linkage), and 3 distance metrics were examined in calculating the dissimilarity matrix (Spearman, Kendall and Pearson). Overall, the methods used for distance calculations or dendrograms did not affect the clustering pattern. The clustering pattern was further corroborated with principal component analysis (PCA) of the centroid data obtained from GEDI analysis (Fig. 7B). Consistent with hierarchical clustering, the distance between wild type and α9^−/−^ mice at P13 was evident in the PCA sample plane.

Coupling the GEDI clustering with the second-level analyses described revealed the disparity between wild type and α9^−/−^ cochleae at P13. At P3 and P7, genome-wide expression patterns of wild type and α9^−/−^ samples clustered together. By P13, the wild type transcriptome was distinctly separated from the earlier postnatal ages, whereas the α9^−/−^ data were more similar to, and clustered with, data from earlier postnatal ages. At P60, the wild type and α9^−/−^ cochleae clustered together, albeit not as tightly as at P3 and P7.

## Discussion

We have analyzed nicotinic cholinergic synapse stabilization and maintenance in the mouse cochlea using an approach based on comparative gene expression profiling of α9^−/−^ animals and age-matched wild type controls. The α9^−/−^ mice possess dramatically altered synaptic bouton structure and hair cell efferent innervation. Our goal was to define genes and pathways that were altered in α9^−/−^ mice exhibiting abnormal innervation patterns during development as a consequence of gene ablation restricted in space (in the cochlea) to the postsynaptic hair cells, which represent the target of the descending nicotinic efferent innervation. Cochleae were sampled at various postnatal ages spanning the critical period of structural and functional synaptic maturation (prior to efferent synapse formation- P3; during the height of efferent synaptic formation- P7), the onset of hearing in mouse (first stages of stabilization of efferent innervation and afferent function - P13), and finally the adult-like maintenance of these synapses (P60). Using microarray analyses, we have defined global gene expression alterations with respect to the wild type condition brought about as a consequence of ablation of the key nicotinic subunit responsible for nAChR activity in the inner ear. We have confirmed gene expression level changes using qPCR analyses, and also observed concordant changes for a number of the differentially expressed genes at the protein expression level for select molecules of particular interest for cochlear processing.

### Changes Induced by Loss of the α9 Subunit Are Most Pronounced after Onset of Hearing

Our analyses of individual transcripts revealed that consequences following ablation of α9 subunit expression are greatest at post-hearing ages (P13 and P60). This was an unexpected finding, given that structural alterations to the efferent synapse as a consequence of α9 gene ablation have been described as early as P3 [13]. Quantitatively, the number of differentially expressed molecules was the highest at the onset of hearing compared to all other ages, suggesting that changes in the transcriptome parallel the onset of the more coordinated, sensitive auditory function characteristic of adult-like hearing. Additionally, SOM analyses coupled with hierarchical clustering and PCA revealed a striking difference in the transcriptomes of wild type and α9^−/−^ samples at the onset of hearing (P13), which was marked by sharp divergence of α9^−/−^ transcriptome from the wild type state, and its similarity to earlier, immature postnatal ages. Prior evidence points to a link between auditory maturation and intact efferent innervation. Efferent feedback can alter cochlear thresholds and is required for development of normal cochlear function and morphology as suggested in studies of de-efferented neonatal cats [43]. In general, spontaneous waves of endogenous nicotinic activity are common in the developing nervous system and participate in development of synaptic circuitry [44]. Efferents likely play a role in regulating immature IHC spiking activity, which in turn could be important in establishing the mature pattern of innervation and function in the developing auditory pathway [45]–[47]. Therefore, loss of efferent, nicotinic receptor-based cholinergic activity may alter the critical period of functional maturation of the cochlea, as has been suggested upon examination of the spatiotemporal innervation characteristics of the α9 null mice [13]. As has been shown for the visual system [48], [49], peripheral activity can play a large role in establishing correct CNS innervation patterns, and thus a misregulated efferent system could potentially result in abnormal central nervous system representations of the auditory periphery. However, it should be noted that no description of cellular loss or deficiency in cell number or type has ever been described for the α9^−/−^ mice. Given the lack of ABR threshold changes in these mice, it is apparent that the adult afferent system is functionally intact, further suggesting that within the cochlea, changes induced by loss of α9 gene expression as described here are most likely limited to the efferent cholinergic system. How this may have altered early developmental programs in the cochlea that are important to CNS auditory pathway development is currently unknown.

### Loss of α9 Receptor Subunit Activates a Variety of Pathways and Gene Sets

**Figure 8:**
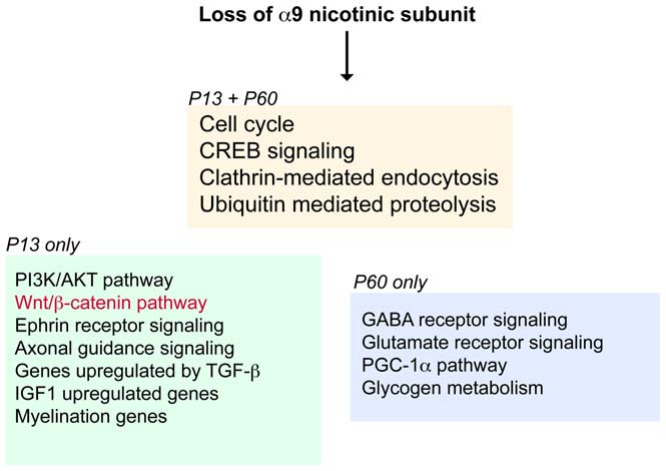
Summary of over-represented gene categories. Several functional categories, based on PANTHER annotations and GSEA results, are significantly perturbed upon loss of α9 nicotinic subunit at postnatal time periods P13 and P60. While several processes are common to both ages (orange box), others are preferentially expressed at P13 (green box) or P60 (blue box). Only Wnt/β-catenin pathway (highlighted in red) is identified as over-represented in α9^−/−^ by both PANTHER and GSEA analyses.

Several pathways known to be involved in synapse formation in the central nervous system also show differential enrichment upon loss of α9 subunits (Fig. 8). As suggested by ontology classifications of individual genes and enrichment of specific gene sets, ephrin receptor signaling and various signaling pathways such as PI3K/AKT and Wnt/β-catenin are perturbed at P13. Additionally we have used qPCR techniques to confirm the up-regulation of *Epha3 (Eph receptor A3*) the putative receptor for ephrin-A5, at P60. Ephrins are involved in axonal guidance during the formation of the connections of the CNS. Recent evidence indicates that Ephrin/Eph signaling may participate in the formation of synapses and dendritic spines, and the ephrin family has been implicated in cochlear development [50]–[52]. Genes involved in the PI3K signaling pathway were also found to be differentially expressed, and have been shown to be involved in nerve growth cone dynamics [53], neurite growth [54] and synapse function [55].

Together with GO classifications, statistical analyses of gene sets indicate involvement of several pathways and molecules that may mediate the effects of cholinergic activity. A curated public gene set that involves molecules selectively up-regulated by IGF-1 shows a switch in the sign of the enrichment score over postnatal development, indicating up-regulation in the α9^−/−^ animals at P3 and down-regulation at P13. Particularly, IGF-1 is a polypeptide hormone with pleiotropic systemic effects that has also been implicated in neuro-protection, synaptic plasticity and synapse formation [56]–[58]. In the cochlea, lack of IGF-1 results in abnormal synaptogenesis, decreased myelination and deficient innervation of the sensory cells in mice [59], [60]. While IGF itself was not differentially regulated following loss of α9 expression, the genes associated with its signaling pathways were changing coherently in our analysis, indicating that activity profiles similar to those induced by IGF activation could be perturbed in the α9^−/−^ mice. Equally important, the TGF-β and Wnt signaling pathways also show significant differential expression at P13. Both TGF-β and Wnt signaling have been characterized at the NMJ where they play key roles in synaptic assembly and growth [61], [62]. An over-representation of genes associated with the PGC-1α pathway is also complementary to findings involving the NMJ, where PGC-1α is a key regulator of NMJ structure and activity [63]. Additionally, enrichment of the CREB pathway at both P13 and P60 is important since nicotinic signaling has been shown to lead to activation of CREB [23], [64], [65]. Further, the ubiquitin-proteasome system exhibits functional enrichment at both P13 and P60. Protein degradation is known to have important roles in neuronal development and long-term synaptic plasticity [66], and proteasome inhibition can alter plasticity at the synapse by leading to both presynaptic and postsynaptic changes controlling synaptic strength and growth [67]. Taken together, abnormal synaptic and innervation phenotypes seen in the cochleae of α9 null animals are likely due to misregulation of several, not single, pathways that converge to alter transcriptional mechanisms (such as CREB, Wnt and TGF-β signaling).

### Loss of α9 Subunit Expression Disturbs Ionic Homeostasis at P60, and Results in Abnormal GABA Receptor Subunit Expression

Ontological classifications of differentially expressed genes at P60 show up-regulation of several ion channel families and related molecules, suggesting that lack of cholinergic activity perturbs expression levels of various ion channels. Mature hair cells express a rich variety and abundance of ion channels that serve to modulate the receptor potential, and altered expression of any may have functional consequences. Several molecules involved in voltage-gated K^+^ channels, voltage-gated Na^+^ channels, glutamatergic and GABAergic receptors, along with genes encoding a number of downstream signaling and auxiliary molecules such as *CamkIV* and *Kcnip4*, are up-regulated in the α9^−/−^ mouse. One of the voltage-gated Na^+^ channels, *Nav1.2*, is detected in the unmyelinated portions of efferent axons and their endings beneath inner and outer hair cells, and is presumably responsible for refining axonal firing rates [68]. The altered transcriptional expression of voltage-gated Na^+^ channels observed in the α9^−/−^ mice suggests that lack of a functional postsynaptic nAChR may lead to changes along the efferent axons that innervate both inner and outer hair cells. Because these synaptic terminals have been shown to be neurochemically complex, and may express more than one neurotransmitter [69], altered postsynaptic nAChR activity may play a significant role in regulating pre-synaptic release of other neuroactive molecules from the olivocochlear terminal via regulation of efferent axonal activity.

Equally important, lack of cholinergic activity alters transcriptional levels of major components of A-type K^+^ currents. These types of currents regulate the frequency and amplitude of action potentials. In the CNS, immunohistochemical analysis shows that *Kcnd2* (Kv4.2) is expressed in rat striatal cholinergic interneurons [70] and is clustered on the postsynaptic membrane [71]. In chick cochleae, Kv4.2 is expressed post-synaptically by ganglion cells and at the basolateral aspects of the intermediate and short hair cells (analogs of mammalian outer hair cells), but is absent in tall hair cells (analogs of mammalian inner hair cells) [72], [73]. Thus, spatial expression patterns of Kv4.2 expression suggest that it modulates the electrical signals originating from efferent synaptic activity. The second identified component of A-type potassium currents from our microarray screens is *Kcnip4*, a member of the calcium-binding protein family of KChIPs (Kv channel interacting proteins), all of which bind to cytoplasmic Kv4 channel N*-*termini. Thus, KChIPs are likely to play a major role in coupling A-current density to activity-dependent fluctuations in intracellular calcium levels [74], [75]. Taken together, these data suggest that loss of nicotinic cholinergic activity, and by extension, the associated calcium influx, may alter the expression of various ion channels, and potentially alter basic hair cell physiological profiles. Given the array data indicating a delayed maturation profile following loss of α9 gene expression, one may postulate a delayed maturation of such profiles.

GABA_A_ receptors are ligand-gated Cl^−^ ion channels that mediate fast synaptic transmission at the vast majority of inhibitory synapses in the mammalian brain. In addition to cholinergic markers, the olivocochlear terminals also have a significant representation of GABAergic elements [69]. It has been suggested that the GABA system plays a trophic role in the mammalian cochlea by maintaining the stability of innervation such that loss of several GABA_A_ receptor subunits results in auditory dysfunction and loss of innervation [26]. Of particular interest is the current finding that α9 subunit expression/nAChR activity modulates expression of a wide range of GABA receptor subunits. In the present microarray screens, an increased expression of various GABA receptor subunits was observed, and qPCR analyses have confirmed the changes at the transcript level. We have further validated the changes at the protein expression level for GABA_A_α1 and GABA_A_β2. These subunits were chosen based on the magnitude of their differential expression, and their abundance in the CNS. Although loss of the α1 subunit has thus far not been shown to lead to an auditory phenotype, loss of β2 subunit results in neural dysfunction in the cochlea, decreased density of efferent terminals on OHCs, as well as degeneration of efferent innervation after 6 weeks [26]. We have attempted to further define the identity of the cells giving rise to the increased GABA_A_β2 expression by performing immunohistochemical labeling. While OHCs were most prominently labeled in the α9^−/−^ cochlea, Deiters' cells were also more intensely immunoreactive than wild type, although not as intense as the OHCs. Interestingly, Deiters' cells have been shown to receive some neural input [76], [77] and have been suggested to express functional α9-like ACh receptors [78]. Final determination of whether the increased imunolabeling corresponds to an increase in expression awaits single cell qPCR analysis in the future. In addition to changes in the ionotropic GABA_A_ subunits, we have also observed a change in the G protein-coupled receptor GABA_B_β1 subunit at the transcript level. Loss of GABA_B_β1 has been shown to lead to outer hair cell dysfunction [79]. The increased gene expression of GABA receptor subunits following loss of the α9 subunit (and therefore silencing of the nAChR) suggests a relationship between the nicotinic and GABAergic systems at the level of gene expression, but a causal link has yet to be demonstrated. However, interactions between the cholinergic and GABAergic systems have been previously described. In rodent hippocampal interneurons and chick ciliary ganglion neurons, α7 nicotinic receptors (a member of the α-bungarotoxin sensitive family of nAChR subunits to which α9 also belongs) are juxtaposed to GABA_A_ receptors. Activation of nAChRs composed at least in part by α7 subunits down-regulates GABA-induced currents, suggesting modulatory interactions between cholinergic signaling and consequences of inhibitory neurotransmission [80], [81]. Furthermore, nicotinic activity determines the time during development when GABAergic signaling converts from excitation to inhibition by modulating the expression of chloride transporters in chick ciliary ganglion and rodent hippocampal neurons [82]. Similarly, in the mammalian cochlea, GABAergic signaling may act synergistically with nicotinic cholinergic signaling in shaping peripheral output and multi-tiered stages of postnatal development dependent on precise aspects of neural activity. However, future work is needed to evaluate whether such changes in the GABA system can also be attributed to structural interactions that might be lost when the α9 gene is ablated.

### Biclustering Identifies a Neuronal Module Altered at All Developmental Ages Following Loss of α9 Gene Expression

The use of gene-set analytic methods for detecting differential expression is limited by the availability of relevant, pre-defined gene sets appropriate for particular studies. Many novel areas of research may involve physiologically important molecular interactions or functions that are still not well known or characterized, meaning that the available gene set collections may not be optimal for the interpretation of data from such studies. To address this problem, we identified a context-dependent gene cluster representing groups of molecules with specific functions in the nervous system. We utilized a biclustering technique (ISA) to find genes with coherent expression patterns in the hippocampus, striatum and amygdala of adult wild type mice. Of the 33 biclusters thus identified, bicluster9 was chosen for further analysis based on its significant enrichment at all the developmental ages.

Functional annotation of this bicluster suggested its role in several pathways studied in neuronal systems. Additionally, several molecules that contribute highly to the enrichment score are known to be involved in either synapse formation, or are linked to cholinergic synapses, and may serve as candidates for further studies. Specifically, *PSD93* is known to play a role in stabilization of receptor clusters at neuronal cholinergic synapses, and PSD scaffolding molecules are linked to cytoskeletal processes, transcriptional regulation and protein phosphorylation, and can relay the intracellular effects of synaptic activation [83]. Another gene, *Mef2a*, also contributes to the leading edge set at all ages. The MEF2 family of transcription factors is highly expressed in the brain and play specific roles in dendritic maturation and synapse formation [84]. Furthermore, activity-dependent calcium signaling induces dephosphorylation of *Mef2a*, which leads to postsynaptic differentiation in the cerebellar cortex [85]. Overall, it is of interest that a bicluster derived from CNS structures has also generalized well in peripheral tissues such as the cochlea, further emphasizing that cholinergic synapses in the cochlea containing α9α10 nicotinic cholinergic receptors may be used as a model system to study general nicotinic cholinergic synaptic development, stabilization, and neurotransmission. Our validation attempt on two genes that were elements of bicluster9 revealed that one was validated by qPCR, while a second, stomatin, could not be validated. However, gene set enrichment algorithms are designed to pull out lower level expression level changes coordinated across genes that may share common functions. Thus, while stomatin expression change was low and not statistically different from wild type levels, further analysis of this and other members of the gene sets may still be warranted based on unknown biological significance of these small changes to expression. This highlights issues concerning overlap (or lack thereof) between statistical and biological significance.

### Conclusions

Our findings suggest coordinated or sequential involvement of molecules and pathways during nicotinic cholinergic synapse development in the cochlea. The results have defined a number of genes that undergo changes in expression following loss of the α9 nicotinic subunit. The pattern of altered gene expression also suggests a depressed maturation of gene expression during onset of auditory function. This is indicated by differential expression of functional gene sets normally active during early stages of synapse formation, including Wnt/β-catenin signaling and axonal guidance signaling, that remain up-regulated during a time that in the wild type, expression levels normally fall to stable adult levels. Furthermore, lack of α9 receptor subunit expression may result in perturbed ionic homeostasis in adult cochlear hair cells, as indicated by altered expression of numerous voltage-gated ion channels. However, given the large number of channels serving similar roles in hair cell physiology, it is also possible that compensation could occur, thus minimizing basic homeostatic dysfunction occurring as a consequence of abnormal nAChR function. Finally, there is a significant increase in several GABA receptor subunits, suggesting co-varying expression between the nicotinic cholinergic system and the GABAergic system in the cochlea. GABAergic activity is known to play a role in normal CNS development [86], and loss of GABAergic receptor subunits have functional consequences for cochlear processing [26]. Currently, our understanding of the significance of the GABAergic system in cochlear development and function is rudimentary, and thus the finding that the α9^−/−^ mice exhibit altered GABAergic system expression may indicate that this mouse line could be useful in the future to further explore the role(s) of the GABA system in auditory periphery. In summary, our data highlight a number of novel avenues to begin exploring to determine the full set of functional consequences that ensue with the loss of the α9 gene.

## Methods

### Vertebrate Animal Use

This study uses genetically manipulated and wild type mice. All procedures presented herein involving these animals have been conducted in accord with NIH established guidelines published by the Office of Laboratory Animal Welfare (http://grants.nih.gov/grants/olaw/references/phspol.htm) of the NIH Office of Extramural Research.

All animal work was performed at Tufts Univ. School of Medicine. Procedures and facilities in place to ensure humane and proper use and welfare of the mice include Tufts University IACUC approval of all procedures used during the investigation, and proper husbandry of the mice through Tufts University's Division of Lab Animal Management. Mice were maintained in the Tufts vivarium, an AAALAC approved facility, with ad lib access to food and water.

### RNA Isolation and Microarray Hybridization

All animals at each age were sacrificed by cervical dislocation. Cochleae from α9^−/−^ and age-matched wild type mice were harvested at several developmental time periods (P3, P7, P13 and P60), cerebellar fragments, semi-circular canals, and auditory nerve were removed, and the remaining cochlear tissue immediately frozen and stored in liquid nitrogen. All dissections, no matter the age, were identical. Only successfully isolated whole cochleae were retained for analysis (any cochleae fractured during dissection were discarded). Triplicates of pools of 8–10 cochleae or 4–6 cochleae were used for P3/P7 and P13/P60 respectively. Total RNA was isolated using TRIzol reagent (Invitrogen, Carlsbad, CA) according to the manufacturer's protocol. Total RNA concentration was quantified using NanoDrop spectrophotometer (Thermo Scientific, Wilmington, DE), and integrity was checked with the Bioanalyzer 2100 (Agilent, Palo Alto, CA). For the whole-transcriptome profiling, RNA were reverse transcribed, labeled and hybridized to Affymetrix Mouse Genome 430.2 GeneChips by using One-Cycle Labeling Kit (Affymetrix, Santa Clara, CA). Fluidics Station 450 and GCS3000 were used for subsequent washing and scanning steps.

All microarray data are MIAME compliant, and along with design parameters have been deposited in NCBI Gene Expression Omnibus Database (GEO; http://www.ncbi.nlm.nih.gov/geo/) under the accession number GSE18567.

### Statistical Analysis

Affymetrix CEL files were imported into the R statistical software (v2.8.1; http://www.R-project.org). Normalization was performed with the AffyPLM package in BioConductor (v2.4), using RMA background correction, quantile normalization, and the Tukey biweight summary method. Data were filtered by variance (variance quantile <0.60) to remove noise and to increase detection power [87]. Differential expression was detected using the limma package [19], which essentially computes a modified *t* test evaluating the significance of the difference between a gene's mean expression levels for the two genotypes. The *p*-values were adjusted for multiple testing using the false discovery rate (FDR) approach [20]. A probe set is considered differentially expressed if the FDR adjusted *p*-value is <0.05 and the absolute log fold change ≥1.5.

Functional analysis of gene lists was performed in DAVID [21], [88], using the PANTHER functional annotation classes. PANTHER categories with adjusted *p*-values (Benjamini-Hochberg) <0.05 were considered as significantly over-represented in our gene lists. Additionally, the Ingenuity database (http://www.ingenuity.com) was used to assess over-representation of functional categories; categories with Bonferroni adjusted *p*-value <0.05 were considered as significant. Cluster analysis was based on self-organizing maps and visualized using the Gene Expression Dynamics Inspector (GEDI; v2.1) [41]. For GEDI analysis, raw expression data were quantile-normalized as a group across all ages and genotypes. Further hierarchical clustering (average-linkage) of GEDI map centroids was performed in R using the *hclust* library in the stats package. GEDI map centroids were also subjected to principal component analysis (PCA) using MATLAB (MathWorks Inc., Natick, MA). Gene Set Enrichment Analysis was performed using GSEA software v2.0 and MSigDB database v2.5. We assessed the significance of the curated gene sets (MSigDB collection c2) with the following parameters; number of permutations = 1000 and permutation_type  =  gene_set (as suggested for sample sizes with n<7 by GSEA authors), with an FDR q-value cut-off of 5%.

### Biclustering of Microarray Data

Data for biclustering analysis were downloaded from Gene Expression Omnibus (GEO) with the series accession numbers GSE9803, GSE4040 and GSE4034. Raw data were quantile-normalized and filtered to remove probes with low variance (variance quantile <0.50) across all samples. Biclustering was performed using the ISA algorithm as described and as implemented in the BiCAT toolbox. Biclustering was performed using the following parameters: *t_g* (gene threshold) = 2, *t_c* (condition threshold) = 1. Biclusters were selected based on the statistical significance of their enrichment score as calculated by the JAVA implementation of the GSEA program.

### Quantitative PCR

Primers for genes of interest were purchased from Qiagen (QuantiTect PCR primer sets, Qiagen, Inc., Valencia, CA). Quantitative reverse transcription-PCR (qRT-PCR) analysis was performed using a one step fast RT-PCR procedure using the QuantiFast SYBR Green RT-PCR kit (Qiagen, Inc.), HotStarTaq DNA polymerase (Qiagen, Inc.), and the QuantiTect primers of choice following manufacturer's recommendations. Triplicate reactions (technical replicates) using the same cochlear RNA used for microarray experiments were performed over biological triplicates for each primer set as separate reactions. Cycle-by-cycle and dissociation curve fluorescence data were collected as a SYBR Green fluorescence assay. PCR reactions were performed on a Stratagene MX3000 instrument (Stratagene, Inc., La Jolla, California). Analysis of relative fold change in gene expression was calculated via the ΔΔCT method [89] using myosin VIIa as a normalizing standard.

### Immunoprecipitation and Western Blotting

Cochleae were isolated from 8-week-old α9^−/−^ and wild type mice. Cerebellum fragments, semi-circular canals, and auditory nerve were removed and whole cochlear lysates were prepared by manual homogenization in lysis buffer containing T-PER Reagent (Pierce), and Protease inhibitor cocktail (Thermo Scientific, Rockford, IL). Samples were centrifuged at 13,000 *g* for 20 min at 4°C to pellet cellular debris. Protein concentration was estimated using a micro-BCA kit (Thermo Scientific, Rockford, IL) with a BSA standard. Immunoprecipitation reactions used either polyclonal anti-GABA_A_ receptor α1 subunit (Chemicon) or anti-GABA_A_ receptor β2 subunit (Millipore) antibodies. Equal amounts of protein extracts (200 µg) from wild type and α9^−/−^ animals were incubated with antibodies against either GABA_A_ receptor β2 (1∶100) or GABA_A_ receptor α1 subunit (1∶100) overnight at 4°C on a rotating shaker. The samples were then mixed with 100 µl of protein A/G agarose beads (Thermo Scientific, Rockford, IL) and incubated with rotation overnight at 4°C on a shaker. The samples were centrifuged to precipitate the agarose bead immune complexes and washed five times with 500 µl of immunoprecipitation buffer (25 mM Tris, 150 mM NaCl; pH 7.2). The pellets were eluted in protein loading buffer, and samples were vortexed and boiled for 5 mins at 95°C. All proteins were resolved in 8% SDS-polyacrylamide gel at 90 V. Proteins were transferred to PVDF membrane (Bio-Rad, Hercules, CA) and membranes were blocked with 5% nonfat dry milk in Tris-buffered saline-Tween-20 for 1 h. Membranes were incubated overnight in primary antibody (1∶500). The primary antibodies were visualized using horseradish peroxidase conjugated anti-rabbit secondary antibodies (1∶2000, Jackson Immunoresearch) and detected using enhanced chemiluminescence system (SuperSignal West Dura, Pierce) and a Kodak Image Station 440cf. Protein expression levels were quantified using densitometric software (UN-SCAN-IT gel; Silk Scientific, Orem, UT).

### Immunostaining

Cochleae from 2 month old wild type and α9^−/−^ mice were perfused through the round and oval windows with 4% paraformaldehyde in 0.1 M sodium phosphate buffer, isolated, post-fixed in the same fixative for 1 h at room temperature and decalcified overnight in 8% EDTA in PBS at room temperature on a rotator. After decalcification, the cochleae were cryoprotected in 30% sucrose/0.9% NaCl overnight at 4°C, and subsequently embedded in a peel away mold in OCT. Frozen sections were cut at 10 µm on a Leica cryostat, mounted on SuperFrost Plus glass microscope slides, and dried on a slide warmer at 37°C for 1–3 hours. Before immunostaining, cochlear sections were blocked with 5% normal donkey serum, 0.5% Triton X-100 for 1 h at room temperature. This was followed by incubation in primary antibody overnight at room temperature, followed by secondary antibody (Alexa Fluor-594 anti-rabbit, Invitrogen, Carlsbad, CA) for 1 h. Anti-GABA_A_β2 antibody (a gift from Dr. Stephen Moss, Tufts Univ. Sch. Med.) was used at a final concentration of 1∶500. Slides were cover slipped with ProLong Gold (Invitrogen, Carlsbad, CA). Samples were examined by using a Leica TCS SP2 AOBS confocal microscope.

## Supporting Information

Figure S1 Flowchart for microarray analysis methods. Following the pre-processing step, three main methods of analyses were carried out in parallel. For each step, the rationale behind the method is described and the algorithm/software used is given in bold. Step 1 details individual gene level analysis that also includes functional annotation of differentially expressed genes using DAVID and Ingenuity Pathway Analysis tools. DAVID is a web-based tool that was used to query for enriched PANTHER terms in our differentially expressed gene lists. PANTHER and Ingenuity Knowledge Base generally assigned differentially expressed genes with distinct molecular functions. Step 2 of the flowchart outlines analysis that was done at the gene set level to identify novel interactions between genes. Finally, in step 3, whole-transcriptome patterns between ages and genotypes are compared using SOMs.
(0.20 MB TIF)

Table S1 Complete list of genes up- and down-regulated at P3, P7, P13 and P60 in cochleae of wild type and α9−/− mice. Differential expression was obtained using linear models (see text) by analyses of gene expression from α9^−/−^ and age-matched wild type controls for each developmental age over biological triplicates. Up- and down-regulated genes with adjusted p-values<0.05 and absolute log fold changes >1.5 are reported at P3 (Table S1A), P7 (Table S1B), P13 (Table S1C), P60(Table S1D).
(0.16 MB PDF)

Table S2 Intersection of differentially expressed genes at P13 and P60. Intersection of differentially expressed genes at P13 and P60 along with their fold changes. Adjusted p-values<0.05, absolute fold changes >1.5.
(0.06 MB PDF)

Table S3 Functional annotations for biclusters obtained with ISA and details for bicluster9. Table S3A contains statistically over-represented Panther categories as identified by DAVID for all 33 biclusters. Table S3B includes the gene content of bicluster9, along with Affymetrix probe identifiers.
(0.40 MB DOC)

Table S4 Leading edge subsets for Bicluster9 for all ages. Leading edge subset of GSEA results for Bicluster9 for P3, P7, P13 and P60. Both Affymetrix Probe IDs and their corresponding gene symbols are given. The rank order of genes in each age indicates their contribution to the running sum score (with genes listed at the top contributing highly).
(0.05 MB PDF)

Video S1 GEDI Self Organizing Maps- WT. GEDI SOMs for wild type cochlear samples at P3, P7, P13 and P60 are represented as a movie to visualize the dynamic pattern of gene expression.
(0.99 MB MOV)

Video S2 GEDI Self Organizing Maps- α9 KO. GEDI SOMs for knockout cochlear samples at P3, P7, P13 and P60 are represented as a movie to visualize the dynamic pattern of gene expression.
(0.97 MB MOV)
