# Supplementary figures and images for: Lack of nAChR Activity Depresses Cochlear Maturation and Up-Regulates GABA System Components: Temporal Profiling of Gene Expression in α9 Null Mice
Source: PLoS One. 2010 Feb 4;5(2):e9058. doi: 10.1371/journal.pone.0009058 (PMC2816210; doi:10.1371/journal.pone.0009058)

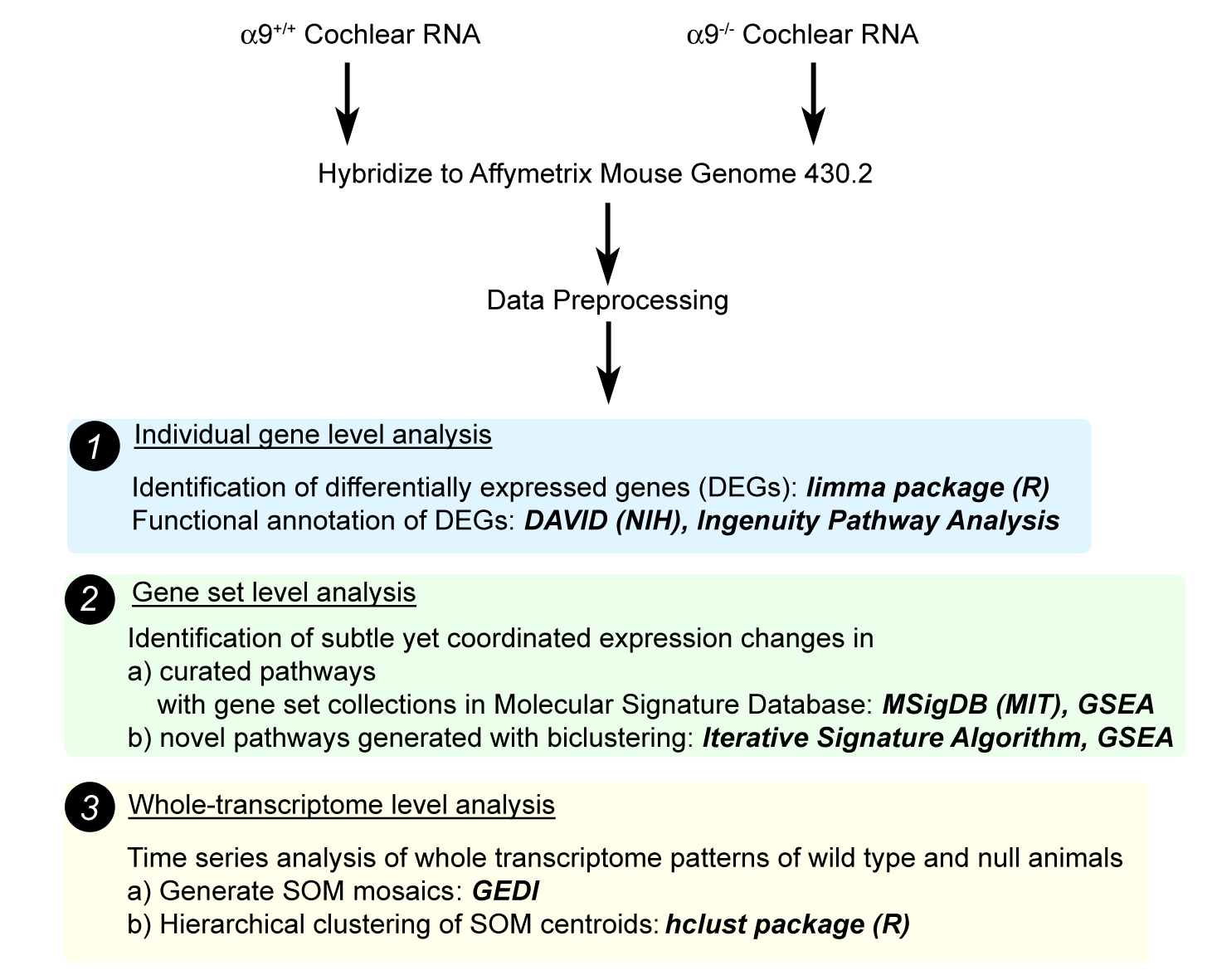

Supplement: Figure S1 — Flowchart for microarray analysis methods. Following the pre-processing step, three main methods of analyses were carried out in parallel. For each step, the rationale behind the method is described and the algorithm/software used is given in bold. Step 1 details individual gene level analysis that also includes functional annotation of differentially expressed genes using DAVID and Ingenuity Pathway Analysis tools. DAVID is a web-based tool that was used to query for enriched PANTHER terms in our differentially expressed gene lists. PANTHER and Ingenuity Knowledge Base generally assigned differentially expressed genes with distinct molecular functions. Step 2 of the flowchart outlines analysis that was done at the gene set level to identify novel interactions between genes. Finally, in step 3, whole-transcriptome patterns between ages and genotypes are compared using SOMs. (0.20 MB TIF) [file pone.0009058.s001.tif]
