# Supplementary material for: Lack of nAChR Activity Depresses Cochlear Maturation and Up-Regulates GABA System Components: Temporal Profiling of Gene Expression in α9 Null Mice
Source: PLoS One. 2010 Feb 4;5(2):e9058. doi: 10.1371/journal.pone.0009058 (PMC2816210; doi:10.1371/journal.pone.0009058)
Supplement: Table S1 — Complete list of genes up- and down-regulated at P3, P7, P13 and P60 in cochleae of wild type and α9−/− mice. Differential expression was obtained using linear models (see text) by analyses of gene expression from α9−/− and age-matched wild type controls for each developmental age over biological triplicates. Up- and down-regulated genes with adjusted p-values<0.05 and absolute log fold changes >1.5 are reported at P3 (Table S1A), P7 (Table S1B), P13 (Table S1C), P60(Table S1D). (0.16 MB PDF) [file pone.0009058.s002.pdf]

**Table S1A - Genes that are differentially regulated in a9-/- cochleas compared to wild-type controls at P3**

| Probe ID | Fold Change (log2) | Gene Symbol | Gene Description |
|---|---|---|---|
| 1441373_at | -3.94 | Msi2 | Musashi homolog 2 (Drosophila) |
| 1435521_at | -3.52 | Msi2 | Musashi homolog 2 (Drosophila) |
| 1448793_a_at | -3.33 | Sdc4 | syndecan 4 |
| 1453540_at | 3.61 | 5430404G13Rik | RIKEN cDNA 5430404G13 gene |
| 1438130_at | -3.17 | Taf15 | TAF15 RNA polymerase II, TATA box binding protein (TBP)-associated factor |
| 1452907_at | -3.10 | Galc | galactosylceramidase |
| 1424893_at | -2.77 | Ndel1 | nuclear distribution gene E-like homolog 1 (A. nidulans) |
| 1458524_at | -2.69 | Fndc3a | fibronectin type III domain containing 3a |
| 1443273_at | 2.40 | Epha3 | Eph receptor A3 |

**Table S1B - Genes that are differentially regulated in a9-/- cochleas compared to wild-type controls at P7**

| Probe ID | Fold Change (log2) | Gene Symbol | Gene Description |
|---|---|---|---|
| 1431708_a_at | 3.73 | Tia1 | cytotoxic granule-associated RNA binding protein 1 |
| 1458934_at | -3.62 | D5Ertd505e | DNA segment, Chr 5, ERATO Doi 505 , expressed |
| 1441172_at | 3.59 | Aff3 | |
| 1435514_at | 2.74 | Lztfl1 | leucine zipper transcription factor-like 1 |
| 1447096_at | 2.51 | | |
| 1452426_x_at | 2.47 | Zfp236 | |
| 1443100_at | -2.23 | Thrb | |
| 1443115_at | 2.18 | Tgfbr2 | |
| 1443772_at | -2.16 | Dzip1 | DAZ interacting protein 1 |
| 1435046_at | 2.14 | | |
| 1436533_at | -2.02 | Trove2 | TROVE domain family, member 2 |
| 1426438_at | -2.01 | Ddx3y | DEAD (Asp-Glu-Ala-Asp) box polypeptide 3, Y-linked |
| 1436936_s_at | 1.97 | Tsix | X (inactive)-specific transcript, antisense |
| 1453540_at | 1.93 | 5430404G13Rik | RIKEN cDNA 5430404G13 gene |
| 1427262_at | 1.86 | Xist | inactive X specific transcripts |
| 1416123_at | -1.85 | Ccnd2 | cyclin D2 |
| 1459725_s_at | 1.83 | Dcpp3 | demilune cell and parotid protein 3 |
| 1448229_s_at | -1.77 | Ccnd2 | cyclin D2 |
| 1455215_at | 1.76 | C530028O21Rik | RIKEN cDNA C530028O21 gene |
| 1420347_at | 1.76 | Plunc | palate, lung, and nasal epithelium associated |
| 1445014_at | 1.73 | AU019157 | expressed sequence AU019157 |
| 1457936_at | 1.72 | Mapk8 | mitogen-activated protein kinase 8 |
| 1420491_at | 1.70 | Eif2s1 | eukaryotic translation initiation factor 2, subunit 1 alpha |
| 1446700_at | 1.68 | | |
| 1423935_x_at | 1.63 | Krt14 | keratin 14 |
| 1422253_at | -1.61 | Col10a1 | collagen, type X, alpha 1 |
| 1430030_at | 1.61 | 5330426P16Rik | RIKEN cDNA 5330426P16 gene |
| 1439300_at | 1.59 | Chic1 | cysteine-rich hydrophobic domain 1 |
| 1455966_s_at | 1.53 | Nudt21 | nudix (nucleoside diphosphate linked moiety X)-type motif 21 |
| 1435462_at | 1.52 | Plcxd2 | phosphatidylinositol-specific phospholipase C, X domain containing 2 |
| 1440278_at | 1.51 | Dynll1 | dynein light chain LC8-type 1 |
| 1458235_at | -1.51 | Itk | |

**Table S1C - Genes that are differentially regulated in a9-/- cochleas compared to wild-type controls at P13**

| Probe ID | Fold Change (log2) | Gene Symbol | Gene Description |
|---|---|---|---|
| 1425711_a_at | 4.01 | Akt1 | thymoma viral proto-oncogene 1 |
| 1451961_a_at | 5.84 | Mbp | myelin basic protein |
| 1431030_a_at | 4.43 | Rnf14 | ring finger protein 14 |
| 1416572_at | 3.95 | Mmp14 | matrix metallopeptidase 14 (membrane-inserted) |
| 1450392_at | 3.48 | Abca1 | ATP-binding cassette, sub-family A (ABC1), member 1 |
| 1431686_a_at | 3.92 | Gmfb | glia maturation factor, beta |
| 1425329_a_at | 3.35 | Cyb5r3 | cytochrome b5 reductase 3 |
| 1448541_at | 3.87 | Klc1 | kinesin light chain 1 |
| 1416191_at | 3.51 | Sec61a1 | Sec61 alpha 1 subunit (S. cerevisiae) |
| 1460661_at | 2.92 | Edg3 | sphingosine-1-phosphate receptor 3 |
| 1420924_at | 3.58 | Timp2 | tissue inhibitor of metalloproteinase 2 |
| 1442019_at | 3.93 | Rcvrn | |
| 1422009_at | 3.29 | Atp1b2 | ATPase, Na+/K+ transporting, beta 2 polypeptide |
| 1419098_at | 3.43 | Stom | stomatin |
| 1415893_at | 2.82 | Sgpl1 | sphingosine phosphate lyase 1 |
| 1428127_at | 2.95 | 4921506J03Rik | predicted gene, ENSMUSG00000074747 |
| 1421857_at | 3.03 | Adam17 | a disintegrin and metallopeptidase domain 17 |
| 1424484_at | 2.74 | Mobkl1b | MOB1, Mps One Binder kinase activator-like 1B (yeast) |
| 1420901_a_at | 3.98 | Hk1 | hexokinase 1 |
| 1433492_at | 2.90 | Epb4.1l2 | erythrocyte protein band 4.1-like 2 |
| 1438661_a_at | 2.73 | Arf2 | ADP-ribosylation factor 2 |
| 1448458_at | 3.19 | Top2b | topoisomerase (DNA) II beta |
| 1434357_a_at | 3.25 | Kpnb1 | karyopherin (importin) beta 1 |
| 1432004_a_at | 2.44 | Dnm2 | dynamin 2 |
| 1439151_at | 2.39 | Msrb3 | methionine sulfoxide reductase B3 |
| 1418018_at | 3.36 | Cpd | carboxypeptidase D |
| 1448538_a_at | 4.25 | D4Wsu53e | DNA segment, Chr 4, Wayne State University 53, expressed |
| 1423325_at | 2.92 | Pnn | pinin |
| 1416568_a_at | 2.74 | Acin1 | apoptotic chromatin condensation inducer 1 |
| 1450311_at | 2.52 | Slc8a3 | solute carrier family 8 (sodium/calcium exchanger), member 3 |
| 1444749_at | 3.59 | | |
| 1416190_a_at | 3.90 | Sec61a1 | Sec61 alpha 1 subunit (S. cerevisiae) |
| 1421606_a_at | 2.41 | Sult4a1 | sulfotransferase family 4A, member 1 |
| 1420975_at | 3.21 | Baz1b | bromodomain adjacent to zinc finger domain, 1B |
| 1450915_at | 2.72 | Ap3b1 | adaptor-related protein complex 3, beta 1 subunit |
| 1421955_a_at | 3.32 | Nedd4 | neural precursor cell expressed, developmentally down-regulated 4 |
| 1429859_a_at | 2.58 | Arl2bp | ADP-ribosylation factor-like 2 binding protein |
| 1443017_at | 2.90 | Cpeb2 | cytoplasmic polyadenylation element binding protein 2 |
| 1433804_at | 3.06 | Jak1 | Janus kinase 1 |
| 1420610_at | 3.02 | Prkacb | protein kinase, cAMP dependent, catalytic, beta |
| 1415784_at | 3.43 | Vps35 | vacuolar protein sorting 35 |
| 1453623_a_at | 3.04 | Rad23a | RAD23a homolog (S. cerevisiae) |
| 1424791_a_at | 2.54 | Bcam | basal cell adhesion molecule |
| 1438683_at | 2.57 | Wasf2 | WAS protein family, member 2 |
| 1458676_at | 3.84 | Nktr | natural killer tumor recognition sequence |
| 1416661_at | 2.58 | Eif3s10 | eukaryotic translation initiation factor 3, subunit A |
| 1421313_s_at | 3.15 | Cttn | cortactin |
| 1427262_at | 2.55 | Xist | inactive X specific transcripts |
| 1423446_at | 2.52 | Dapk3 | death-associated protein kinase 3 |
| 1418170_a_at | 2.51 | Zcchc14 | zinc finger, CCHC domain containing 14 |
| 1422842_at | 2.86 | Xrn2 | 5'-3' exoribonuclease 2 |
| 1422017_s_at | 2.25 | 4833439L19Rik | RIKEN cDNA 4833439L19 gene |

| | | | |
|---|---|---|---|
| 1447869_x_at | 2.54 | Rhobtb3 | Rho-related BTB domain containing 3 |
| 1426975_at | 2.60 | Os9 | amplified in osteosarcoma |
| 1421860_at | 2.23 | Clstn1 | calsyntenin 1 |
| 1427565_a_at | 2.98 | Abcc5 | ATP-binding cassette, sub-family C (CFTR/MRP), member 5 |
| 1422249_s_at | 2.46 | Zfa | zinc finger protein, autosomal |
| 1441108_at | 2.81 | Papola | |
| 1419099_x_at | 3.30 | Stom | stomatin |
| 1426764_at | 2.54 | Oaz2 | ornithine decarboxylase antizyme 2 |
| 1415824_at | 4.00 | Scd2 | stearoyl-Coenzyme A desaturase 2 |
| 1439127_at | 2.79 | AI314180 | expressed sequence AI314180 |
| 1438677_at | 2.48 | Pkp4 | plakophilin 4 |
| 1420816_at | 2.27 | Ywhag | tyrosine 3-monooxygenase/tryptophan 5-monooxygenase activation protein, gamma polypeptide |
| 1442368_at | 2.55 | Kctd12b | potassium channel tetramerisation domain containing 12b |
| 1453740_a_at | 4.21 | Ccnl2 | cyclin L2 |
| 1416157_at | 2.61 | Vcl | vinculin |
| 1420893_a_at | 2.07 | Tgfbr1 | transforming growth factor, beta receptor I |
| 1444001_at | 3.28 | Strbp | |
| 1449292_at | 3.09 | Rb1cc1 | RB1-inducible coiled-coil 1 |
| 1431233_at | 2.93 | Cnnm4 | cyclin M4 |
| 1421198_at | 2.26 | Itgav | integrin alpha V |
| 1435984_at | 2.67 | 1110033F14Rik | zinc finger protein 40 |
| 1453307_a_at | 3.82 | Anapc5 | anaphase-promoting complex subunit 5 |
| 1417033_at | 2.07 | Ube2g2 | ubiquitin-conjugating enzyme E2G 2 |
| 1455986_at | 2.15 | Zdhhc17 | zinc finger, DHHC domain containing 17 |
| 1450977_s_at | 2.62 | Ndrg1 | N-myc downstream regulated gene 1 |
| 1429772_at | 2.14 | Plxna2 | plexin A2 |
| 1426051_a_at | 2.43 | Cenpb | centromere protein B |
| 1422959_s_at | 2.41 | Zfp313 | ring finger protein 114 |
| 1442277_at | 2.54 | Chka | choline kinase alpha |
| 1425461_at | 2.58 | Fbxw11 | F-box and WD-40 domain protein 11 |
| 1421324_a_at | 2.22 | Akt2 | thymoma viral proto-oncogene 2 |
| 1420954_a_at | 2.27 | Add1 | adducin 1 (alpha) |
| 1417496_at | 3.39 | Cp | ceruloplasmin |
| 1420946_at | 2.66 | Atrx | alpha thalassemia/mental retardation syndrome X-linked homolog (human) |
| 1425834_a_at | 2.18 | Gpam | glycerol-3-phosphate acyltransferase, mitochondrial |
| 1425810_a_at | 3.57 | Csrp1 | cysteine and glycine-rich protein 1 |
| 1420941_at | 3.04 | Rgs5 | regulator of G-protein signaling 5 |
| 1427408_a_at | 2.05 | Thrap3 | thyroid hormone receptor associated protein 3 |
| 1451846_at | 3.18 | Nebl | nebulette |
| 1417752_at | 1.98 | Coro1c | coronin, actin binding protein 1C |
| 1425701_a_at | 3.73 | Rgs3 | regulator of G-protein signaling 3 |
| 1418632_at | 2.52 | Ube2h | ubiquitin-conjugating enzyme E2H |
| 1425597_a_at | 2.17 | Qk | quaking |
| 1426165_a_at | 2.64 | Casp3 | caspase 3 |
| 1449578_at | 2.00 | Supt16h | suppressor of Ty 16 homolog (S. cerevisiae) |
| 1446972_at | 2.07 | D15Wsu126e | DNA segment, Chr 15, Wayne State University 126, expressed |
| 1426542_at | 2.29 | Endod1 | endonuclease domain containing 1 |
| 1460304_a_at | 2.04 | Ubtf | upstream binding transcription factor, RNA polymerase I |
| 1448348_at | 2.97 | Caprin1 | cell cycle associated protein 1 |
| 1417831_at | 2.68 | Smc1a | structural maintenance of chromosomes 1A |
| 1452837_at | 2.26 | Lpin2 | lipin 2 |
| 1450097_s_at | 2.26 | Gna12 | guanine nucleotide binding protein, alpha 12 |
| 1438069_a_at | 2.78 | Rbm5 | RNA binding motif protein 5 |
| 1452427_s_at | 2.86 | Ptplad1 | protein tyrosine phosphatase-like A domain containing 1 |
| 1426360_at | 2.79 | Zc3h11a | zinc finger CCCH type containing 11A |

| | | | |
|---|---|---|---|
| 1420831_at | 1.98 | Qsox1 | quiescin Q6 sulfhydryl oxidase 1 |
| 1453604_a_at | 2.29 | Hbs1l | Hbs1-like (S. cerevisiae) |
| 1422967_a_at | 3.44 | Tfrc | transferrin receptor |
| 1415921_a_at | 2.61 | Tnfrsf19 | tumor necrosis factor receptor superfamily, member 19 |
| 1420894_at | 2.16 | Tgfbr1 | transforming growth factor, beta receptor I |
| 1438700_at | 2.65 | Fnbp4 | formin binding protein 4 |
| 1426849_at | 2.07 | Sec24b | Sec24 related gene family, member B (S. cerevisiae) |
| 1452370_s_at | 2.11 | B230208H17Rik | RIKEN cDNA B230208H17 gene |
| 1460460_a_at | 2.05 | Gorasp2 | golgi reassembly stacking protein 2 |
| 1458508_at | 2.43 | Matr3 | matrin 3 |
| 1419049_at | 2.15 | Pcnx | pecanex homolog (Drosophila) |
| 1425797_a_at | 2.05 | Syk | spleen tyrosine kinase |
| 1442309_at | 2.05 | Dnmt3a | |
| 1421895_at | 1.94 | Eif2s3x | eukaryotic translation initiation factor 2, subunit 3, structural gene X-linked |
| 1432344_a_at | 2.20 | Aplp2 | amyloid beta (A4) precursor-like protein 2 |
| 1456473_x_at | 2.24 | Arf2 | ADP-ribosylation factor 2 |
| 1437001_at | 3.56 | Gsk3b | glycogen synthase kinase 3 beta |
| 1421991_a_at | 2.23 | Igfbp4 | insulin-like growth factor binding protein 4 |
| 1442150_at | 2.38 | | |
| 1416601_a_at | 2.38 | Rcan1 | regulator of calcineurin 1 |
| 1437066_at | 2.96 | Zbtb20 | zinc finger and BTB domain containing 20 |
| 1430634_a_at | 2.14 | Pfkp | phosphofructokinase, platelet |
| 1426993_at | 1.97 | Xpr1 | xenotropic and polytropic retrovirus receptor 1 |
| 1458376_at | 2.03 | B930025B16Rik | RIKEN cDNA B930025B16 gene |
| 1419256_at | 2.65 | Spnb2 | spectrin beta 2 |
| 1451048_at | 2.09 | Metap2 | methionine aminopeptidase 2 |
| 1416437_a_at | 2.18 | Mapk8ip3 | mitogen-activated protein kinase 8 interacting protein 3 |
| 1416644_a_at | 1.80 | Sema3b | sema domain, immunoglobulin domain (Ig), short basic domain, secreted, (semaphorin) 3B |
| 1419451_at | 1.93 | Fzr1 | fizzy/cell division cycle 20 related 1 (Drosophila) |
| 1429331_at | 3.04 | 4632427E13Rik | RIKEN cDNA 4632427E13 gene |
| 1429533_at | 2.18 | Immt | inner membrane protein, mitochondrial |
| 1460717_at | 2.46 | Tspyl1 | testis-specific protein, Y-encoded-like 1 |
| 1447176_at | 2.40 | A930008G19Rik | |
| 1426179_a_at | 2.17 | Twsg1 | twisted gastrulation homolog 1 (Drosophila) |
| 1446529_at | 2.70 | 1200003I07Rik | |
| 1452483_a_at | 1.95 | Cd44 | CD44 antigen |
| 1418188_a_at | 3.47 | Ramp2 | receptor (calcitonin) activity modifying protein 2 |
| 1428820_at | 2.64 | Mapre1 | microtubule-associated protein, RP/EB family, member 1 |
| 1417069_a_at | 4.15 | Gmfb | glia maturation factor, beta |
| 1431232_a_at | 2.40 | Mga | MAX gene associated |
| 1439517_at | 2.67 | Mysm1 | |
| 1448285_at | 2.49 | Rgs4 | regulator of G-protein signaling 4 |
| 1448183_a_at | 2.86 | Hif1a | hypoxia inducible factor 1, alpha subunit |
| 1451563_at | -1.94 | Emr4 | EGF-like module containing, mucin-like, hormone receptor-like sequence 4 |
| 1440227_at | 2.95 | Slc5a3 | solute carrier family 5 (inositol transporters), member 3 |
| 1436372_a_at | 2.23 | Pdxdc1 | pyridoxal-dependent decarboxylase domain containing 1 |
| 1450291_s_at | -1.81 | Ms4a4c | membrane-spanning 4-domains, subfamily A, member 4C |
| 1420867_at | 3.31 | Tmed2 | transmembrane emp24 domain trafficking protein 2 |
| 1418019_at | 1.98 | Cpd | carboxypeptidase D |
| 1418175_at | 1.86 | Vdr | vitamin D receptor |
| 1456975_at | 2.11 | Taok1 | TAO kinase 1 |
| 1449011_at | 1.86 | Slc12a7 | solute carrier family 12, member 7 |
| 1452629_at | 2.14 | Safb2 | scaffold attachment factor B2 |
| 1424332_at | 1.81 | Rab40c | Rab40c, member RAS oncogene family |
| 1425576_at | 2.98 | Ahcyl1 | S-adenosylhomocysteine hydrolase-like 1 |

| | | | |
|---|---|---|---|
| 1422862_at | 1.98 | Pdlim5 | PDZ and LIM domain 5 |
| 1452024_a_at | 2.29 | Ldb1 | LIM domain binding 1 |
| 1429003_at | 2.48 | Snw1 | SNW domain containing 1 |
| 1432436_a_at | 2.22 | Ak3 | adenylate kinase 3 |
| 1416691_at | 2.97 | Gtpbp2 | GTP binding protein 2 |
| 1427456_at | 1.76 | Wdfy3 | WD repeat and FYVE domain containing 3 |
| 1459916_at | -2.02 | Pax5 | |
| 1429765_at | 2.18 | 1500005K14Rik | RIKEN cDNA 1500005K14 gene |
| 1450733_at | 1.77 | Bicd2 | bicaudal D homolog 2 (Drosophila) |
| 1429680_at | 1.93 | Tra2a | transformer 2 alpha homolog (Drosophila) |
| 1452815_at | -1.79 | P2ry10 | purinergic receptor P2Y, G-protein coupled 10 |
| 1426519_at | 3.40 | P4ha1 | procollagen-proline, 2-oxoglutarate 4-dioxygenase (proline 4-hydroxylase), alpha 1 polypeptide |
| 1447771_at | 2.32 | | |
| 1456088_at | 2.46 | Xiap | X-linked inhibitor of apoptosis |
| 1433515_s_at | 3.44 | Etnk1 | ethanolamine kinase 1 |
| 1415859_at | 2.61 | Eif3c | eukaryotic translation initiation factor 3, subunit C |
| 1419097_a_at | 1.78 | Stom | stomatin |
| 1415997_at | 3.24 | Txnip | thioredoxin interacting protein |
| 1459668_at | 2.41 | Tank | |
| 1428888_at | 2.04 | Tmem33 | transmembrane protein 33 |
| 1448766_at | 2.04 | Gjb1 | gap junction protein, beta 1 |
| 1435635_at | 2.99 | Pcmtd1 | protein-L-isoaspartate (D-aspartate) O-methyltransferase domain containing 1 |
| 1442312_at | 2.15 | Tbl1xr1 | transducin (beta)-like 1X-linked receptor 1 |
| 1425444_a_at | 2.26 | Tgfbr2 | transforming growth factor, beta receptor II |
| 1449931_at | 2.24 | Cpeb4 | cytoplasmic polyadenylation element binding protein 4 |
| 1430552_a_at | 1.73 | Sbf1 | SET binding factor 1 |
| 1450089_a_at | 2.24 | Srprb | signal recognition particle receptor, B subunit |
| 1450108_at | 2.24 | Kif1a | kinesin family member 1A |
| 1452391_at | 2.16 | Cxadr | coxsackievirus and adenovirus receptor |
| 1424598_at | 3.00 | Ddx6 | DEAD (Asp-Glu-Ala-Asp) box polypeptide 6 |
| 1437372_at | 2.37 | Cpsf6 | cleavage and polyadenylation specific factor 6 |
| 1457492_at | 1.64 | Trio | triple functional domain (PTPRF interacting) |
| 1459133_at | 2.33 | Edem3 | ER degradation enhancer, mannosidase alpha-like 3 |
| 1416365_at | 3.68 | Hsp90ab1 | heat shock protein 90kDa alpha (cytosolic), class B member 1 |
| 1449089_at | 1.73 | Nrip1 | nuclear receptor interacting protein 1 |
| 1436898_at | 2.88 | Sfpq | splicing factor proline/glutamine rich (polypyrimidine tract binding protein associated) |
| 1417338_at | 2.22 | Epb4.2 | erythrocyte protein band 4.2 |
| 1457989_at | 2.43 | Slc4a11 | solute carrier family 4, sodium bicarbonate transporter-like, member 11 |
| 1416237_at | 3.46 | Mpzl2 | myelin protein zero-like 2 |
| 1450747_at | 1.66 | Keap1 | kelch-like ECH-associated protein 1 |
| 1421026_at | 2.31 | Gna12 | guanine nucleotide binding protein, alpha 12 |
| 1448851_a_at | 2.11 | Dnajc5 | DnaJ (Hsp40) homolog, subfamily C, member 5 |
| 1454174_a_at | 1.82 | C330007P06Rik | RIKEN cDNA C330007P06 gene |
| 1450650_at | 2.02 | Myo10 | myosin X |
| 1419156_at | 1.77 | Sox4 | SRY-box containing gene 4 |
| 1430529_at | 1.78 | Csnk1a1 | casein kinase 1, alpha 1 |
| 1450007_at | 2.76 | 1500003O03Rik | RIKEN cDNA 1500003O03 gene |
| 1429660_s_at | 2.54 | Smc2 | structural maintenance of chromosomes 2 |
| 1460486_at | 1.91 | Rabgap1 | RAB GTPase activating protein 1 |
| AFFX-TransRecMur/X57349_M_at | 2.62 | Tfrc | transferrin receptor |
| 1438236_at | 1.70 | Nfia | nuclear factor I/A |
| 1423308_at | 2.03 | Tgoln1 | trans-golgi network protein |
| 1416704_at | 2.16 | Mapk14 | mitogen-activated protein kinase 14 |
| 1425725_s_at | 1.95 | Ppp2r5c | protein phosphatase 2, regulatory subunit B (B56), gamma isoform |
| 1426963_at | 2.85 | Pacs2 | phosphofurin acidic cluster sorting protein 2 |

| | | | |
|---|---|---|---|
| 1416959_at | 2.55 | Nr1d2 | nuclear receptor subfamily 1, group D, member 2 |
| 1424913_at | 1.76 | 2310044G17Rik | RIKEN cDNA 2310044G17 gene |
| 1423603_at | 1.70 | Zfpm1 | zinc finger protein, multitype 1 |
| 1452429_s_at | 2.30 | Abcf1 | ATP-binding cassette, sub-family F (GCN20), member 1 |
| 1451956_a_at | 1.95 | Oprs1 | opioid receptor, sigma 1 |
| 1429362_a_at | 2.19 | Sf3b2 | splicing factor 3b, subunit 2 |
| 1434374_at | 2.23 | B930006L02Rik | RIKEN cDNA B930006L02 gene |
| 1452638_s_at | 3.12 | Dnm1l | dynamin 1-like |
| 1437536_at | 1.74 | Fkrp | fukutin related protein |
| 1450104_at | 2.34 | Adam10 | a disintegrin and metallopeptidase domain 10 |
| 1451927_a_at | 2.46 | Mapk14 | mitogen-activated protein kinase 14 |
| 1440847_at | 1.62 | Mtss1 | metastasis suppressor 1 |
| 1429764_at | 2.22 | 1500005K14Rik | RIKEN cDNA 1500005K14 gene |
| 1434046_at | -1.80 | AA467197 | expressed sequence AA467197 |
| 1444141_at | 2.34 | Snx13 | sorting nexin 13 |
| 1440417_at | 2.45 | D19Ertd409e | DNA segment, Chr 19, ERATO Doi 409, expressed |
| 1425206_a_at | 1.83 | Ube3a | ubiquitin protein ligase E3A |
| 1420523_at | 1.84 | Ccdc50 | coiled-coil domain containing 50 |
| 1419234_at | 2.29 | Helb | helicase (DNA) B |
| 1422526_at | 2.02 | Acsl1 | acyl-CoA synthetase long-chain family member 1 |
| 1416504_at | 1.64 | Ulk1 | Unc-51 like kinase 1 (C. elegans) |
| 1420841_at | 2.29 | Ptprf | protein tyrosine phosphatase, receptor type, F |
| 1435031_at | 2.15 | Tmem120a | transmembrane protein 120A |
| 1449296_a_at | 1.96 | Cnp | 2',3'-cyclic nucleotide 3' phosphodiesterase |
| 1417229_at | 1.90 | Capn1 | calpain 1 |
| 1427414_at | 1.61 | Prkar2a | protein kinase, cAMP dependent regulatory, type II alpha |
| 1423966_at | 2.07 | Cd99l2 | Cd99 antigen-like 2 |
| 1452333_at | 2.01 | Smarca2 | SWI/SNF related, matrix associated, actin dependent regulator of chromatin, subfamily a, member 2 |
| 1424504_at | 1.63 | Rab22a | RAB22A, member RAS oncogene family |
| 1445850_at | 1.53 | Ppp1r12b | protein phosphatase 1, regulatory (inhibitor) subunit 12B |
| 1427754_a_at | 1.66 | Dnm1 | dynamin 1 |
| 1430671_a_at | 1.70 | 0610010K06Rik | RIKEN cDNA 0610010K06 gene |
| 1427285_s_at | 2.12 | Malat1 | metastasis associated lung adenocarcinoma transcript 1 (non-coding RNA) |
| 1418809_at | -1.81 | Pira1 | paired-Ig-like receptor A1 |
| 1430878_at | -1.57 | 2210406H18Rik | RIKEN cDNA 2210406H18 gene |
| 1428130_at | 2.23 | Lman1 | lectin, mannose-binding, 1 |
| 1431655_a_at | 1.68 | Agk | acylglycerol kinase |
| 1448375_at | 1.76 | Tm9sf3 | transmembrane 9 superfamily member 3 |
| 1417209_at | 1.55 | Sertad2 | SERTA domain containing 2 |
| 1452161_at | 2.90 | Tiparp | TCDD-inducible poly(ADP-ribose) polymerase |
| 1435163_at | 2.79 | 9030612M13Rik | RIKEN cDNA 9030612M13 gene |
| 1451743_at | 1.68 | D19Wsu162e | DNA segment, Chr 19, Wayne State University 162, expressed |
| 1421645_at | 2.13 | 9930013L23Rik | RIKEN cDNA 9930013L23 gene |
| 1449325_at | 1.54 | Fads2 | fatty acid desaturase 2 |
| AFFX-TransRecMur/X57349_5_at | 2.70 | Tfrc | transferrin receptor |
| 1420873_at | 2.10 | Twf1 | twinfilin, actin-binding protein, homolog 1 (Drosophila) |
| 1418245_a_at | 2.08 | Rbm9 | RNA binding motif protein 9 |
| 1422966_a_at | 2.01 | Tfrc | transferrin receptor |
| 1457275_at | 2.62 | Dmn | desmuslin |
| 1419288_at | 1.77 | Jam2 | junction adhesion molecule 2 |
| 1428150_at | 1.62 | Coro7 | coronin 7 |
| 1419568_at | -1.99 | Mapk1 | mitogen-activated protein kinase 1 |
| 1422853_at | 1.54 | Shc1 | src homology 2 domain-containing transforming protein C1 |
| 1418612_at | -1.56 | Slfn1 | schlafen 1 |
| 1457930_at | 2.25 | Jarid1c | jumonji, AT rich interactive domain 1C (Rbp2 like) |

| | | | |
|---|---|---|---|
| 1438271_at | 2.25 | Lpp | LIM domain containing preferred translocation partner in lipoma |
| 1417029_a_at | 2.51 | Trim2 | tripartite motif-containing 2 |
| 1452448_at | 1.71 | Aqr | aquarius |
| 1432419_a_at | 1.86 | 2700078K21Rik | RIKEN cDNA 2700078K21 gene |
| 1455857_a_at | 1.66 | Rab2b | RAB2B, member RAS oncogene family |
| 1421102_a_at | 2.69 | Vamp3 | vesicle-associated membrane protein 3 |
| 1454655_at | 2.23 | Dgkd | diacylglycerol kinase, delta |
| 1450076_at | 2.21 | 4933411K20Rik | RIKEN cDNA 4933411K20 gene |
| 1447360_at | 4.97 | Tsc22d1 | TSC22 domain family, member 1 |
| 1441435_at | 1.70 | Tbl1x | |
| 1423433_at | 1.67 | Trove2 | TROVE domain family, member 2 |
| 1440841_at | 2.69 | Ywhae | |
| 1450524_at | 2.02 | Cldn9 | claudin 9 |
| 1422793_at | 1.62 | Pafah1b2 | platelet-activating factor acetylhydrolase, isoform 1b, alpha2 subunit |
| 1453760_at | 2.62 | Mier1 | mesoderm induction early response 1 homolog (Xenopus laevis |
| 1417381_at | 2.25 | C1qa | complement component 1, q subcomponent, alpha polypeptide |
| AFFX-TransRecMur/X57349_3_at | 1.99 | Tfrc | transferrin receptor |
| 1423495_at | 1.79 | Decr2 | 2-4-dienoyl-Coenzyme A reductase 2, peroxisomal |
| 1458125_at | 1.67 | C80913 | |
| 1419155_a_at | 1.68 | Sox4 | SRY-box containing gene 4 |
| 1460279_a_at | 3.99 | Gtf2i | general transcription factor II I |
| 1423184_at | 1.63 | Itsn2 | intersectin 2 |
| 1417637_a_at | 2.21 | Hmg20b | high mobility group 20 B |
| 1442761_at | 1.79 | Tpd52l2 | |
| 1435053_s_at | 1.78 | Plekhh1 | pleckstrin homology domain containing, family H (with MyTH4 domain) member 1 |
| 1431464_a_at | 1.87 | Pmm2 | phosphomannomutase 2 |
| 1431374_at | 1.90 | 6330407A03Rik | RIKEN cDNA 6330407A03 gene |
| 1434020_at | 2.50 | Pdap1 | PDGFA associated protein 1 |
| 1439672_at | 1.71 | Synj1 | |
| 1456610_at | 2.89 | Jmjd3 | jumonji domain containing 3 |
| 1431680_a_at | 1.78 | Ptprk | protein tyrosine phosphatase, receptor type, K |
| 1434101_at | 2.07 | Nfib | nuclear factor I/B |
| 1456498_at | 1.86 | Itga4 | integrin alpha 4 |
| 1454633_at | 2.27 | Etnk1 | ethanolamine kinase 1 |
| 1437859_x_at | 3.47 | Eif5a | eukaryotic translation initiation factor 5A |
| 1418231_at | 1.68 | Lims1 | LIM and senescent cell antigen-like domains 1 |
| 1421851_at | 3.88 | Mtap1b | microtubule-associated protein 1B |
| 1448868_at | 2.03 | Scand1 | SCAN domain-containing 1 |
| 1437870_at | -1.88 | Slco4c1 | solute carrier organic anion transporter family, member 4C1 |
| 1454665_at | 2.39 | Irf2bp2 | interferon regulatory factor 2 binding protein 2 |
| 1445991_at | 1.64 | Lhfp | |
| 1439195_at | 1.84 | | |
| 1445204_at | 1.73 | Ccdc85a | |
| 1460650_at | 1.99 | Atp6v0a1 | ATPase, H+ transporting, lysosomal V0 subunit A1 |
| 1425904_at | 2.23 | Satb2 | special AT-rich sequence binding protein 2 |
| 1450037_at | 2.96 | Usp9x | ubiquitin specific peptidase 9, X chromosome |
| 1440346_at | 1.79 | Jmjd3 | jumonji domain containing 3 |
| 1443053_at | 2.56 | | |
| 1450382_at | 1.63 | Nf2 | neurofibromatosis 2 |
| 1449932_at | 2.38 | Csnk1d | casein kinase 1, delta |
| 1453612_at | 1.91 | Nek1 | NIMA (never in mitosis gene a)-related expressed kinase 1 |
| 1457297_at | 2.56 | Mef2a | |
| 1449551_at | 3.02 | Myo1c | myosin IC |
| 1437772_s_at | 2.53 | Hmgcl | fucosidase, alpha-L- 1, tissue |
| 1438235_at | 1.84 | | |

| | | | |
|---|---|---|---|
| 1418532_at | 2.26 | Fzd2 | frizzled homolog 2 (Drosophila) |
| 1449262_s_at | 2.10 | Lin7c | lin-7 homolog C (C. elegans) |
| 1416501_at | 2.95 | Pdpk1 | 3-phosphoinositide dependent protein kinase-1 |
| 1439950_at | 2.10 | Dync1h1 | dynein cytoplasmic 1 heavy chain 1 |
| 1456827_at | 2.29 | Zfp87 | zinc finger protein 87 |
| 1427764_a_at | 2.88 | Tcfe2a | transcription factor E2a |
| 1422082_a_at | 1.84 | Nfya | nuclear transcription factor-Y alpha |
| 1422864_at | 1.56 | Runx1 | runt related transcription factor 1 |
| 1440248_at | 2.03 | Casc4 | cancer susceptibility candidate 4 |
| 1440104_at | 2.23 | Ranbp2 | RAN binding protein 2 |
| 1439651_at | 1.77 | Lmo4 | |
| 1458176_at | 2.57 | Per3 | period homolog 3 (Drosophila) |
| 1450259_a_at | 2.51 | Stat5a | signal transducer and activator of transcription 5A |
| 1454592_at | 2.48 | 9430012M22Rik | RIKEN cDNA 9430012M22 gene |
| 1453164_a_at | 1.58 | Ptdss2 | phosphatidylserine synthase 2 |
| 1451054_at | -1.56 | Orm1 | orosomucoid 1 |
| 1460394_a_at | 1.60 | Inppl1 | inositol polyphosphate phosphatase-like 1 |
| 1432304_a_at | 1.55 | 9030624J02Rik | RIKEN cDNA 9030624J02 gene |
| 1456080_a_at | 4.94 | Serinc3 | serine incorporator 3 |
| 1429327_at | 2.23 | Sdccag1 | serologically defined colon cancer antigen 1 |
| 1427742_a_at | 2.04 | Klf6 | Kruppel-like factor 6 |
| 1420927_at | 1.86 | St6gal1 | beta galactoside alpha 2,6 sialyltransferase 1 |
| 1453742_at | 2.04 | Vps33a | vacuolar protein sorting 33A (yeast) |
| 1417623_at | 2.63 | Slc12a2 | solute carrier family 12, member 2 |
| 1436654_at | -1.71 | Gen1 | Gen homolog 1, endonuclease (Drosophila) |
| 1450208_a_at | 1.84 | Elmo1 | engulfment and cell motility 1, ced-12 homolog (C. elegans) |
| 1440925_at | 2.36 | | |
| 1431646_a_at | 1.64 | Stx6 | syntaxin 6 |
| 1425609_at | 1.71 | Ncf1 | neutrophil cytosolic factor 1 |
| 1424720_at | 1.79 | Mgat4b | mannoside acetylglucosaminyltransferase 4, isoenzyme B |
| 1426951_at | 2.62 | Crim1 | cysteine rich transmembrane BMP regulator 1 (chordin like) |
| 1437118_at | 1.69 | Usp7 | ubiquitin specific peptidase 7 |
| 1421143_at | 1.53 | Diap1 | diaphanous homolog 1 (Drosophila) |
| 1438807_at | 1.74 | Hnrnpr | heterogeneous nuclear ribonucleoprotein R |
| 1437667_a_at | 1.82 | Bach2 | BTB and CNC homology 2 |
| 1452885_at | 2.29 | Sfrs2ip | splicing factor, arginine/serine-rich 2, interacting protein |
| 1425668_a_at | 2.29 | St3gal4 | ST3 beta-galactoside alpha-2,3-sialyltransferase 4 |
| 1437120_at | 1.52 | Snx30 | sorting nexin family member 30 |
| 1449042_at | 1.78 | Ctcf | CCCTC-binding factor |
| 1437060_at | -1.60 | Olfm4 | olfactomedin 4 |
| 1450040_at | 1.69 | Timp2 | tissue inhibitor of metalloproteinase 2 |
| 1451081_a_at | 2.06 | Tcf25 | transcription factor 25 (basic helix-loop-helix) |
| 1456505_at | 1.94 | Braf | Braf transforming gene |
| 1438294_at | 1.90 | Atxn1 | ataxin 1 |
| 1447343_at | 1.83 | Nrp2 | neuropilin 2 |
| 1423048_a_at | 1.50 | Tollip | toll interacting protein |
| 1452606_at | -1.80 | Mnd1 | meiotic nuclear divisions 1 homolog (S. cerevisiae) |
| 1449877_s_at | -1.73 | Kifc1 | similar to Kifc1 protein |
| 1460241_a_at | 2.99 | St3gal5 | ST3 beta-galactoside alpha-2,3-sialyltransferase 5 |
| 1424755_at | 1.51 | Hip1 | huntingtin interacting protein 1 |
| 1440984_at | 2.26 | Baz2b | bromodomain adjacent to zinc finger domain, 2B |
| 1448332_at | 1.95 | Pex19 | peroxisome biogenesis factor 19 |
| 1420908_at | 1.89 | Cd2ap | CD2-associated protein |
| 1416482_at | 1.98 | Ttc3 | tetratricopeptide repeat domain 3 |
| 1437422_at | 1.70 | Sema5a | sema domain, 7 thrombospondin repeats (type 1 and type 1-like), transmembrane domain and short cytoplasmic domain, (semaphorin) 5A |

| | | | |
|---|---|---|---|
| 1421184_a_at | 1.55 | Mettl7a1 | methyltransferase like 7A1 |
| 1449829_at | -1.97 | Itgb2l | integrin beta 2-like |
| 1421866_at | 2.83 | Nr3c1 | nuclear receptor subfamily 3, group C, member 1 |
| 1457062_at | 1.76 | 1700081L11Rik | RIKEN cDNA 1700081L11 gene |
| 1442435_at | 1.63 | Smg6 | |
| 1453988_a_at | 2.87 | Ide | insulin degrading enzyme |
| AFFX-GapdhMur/M32599_5_at | 5.34 | Gapdh | glyceraldehyde-3-phosphate dehydrogenase |
| 1442041_at | 1.95 | LOC552876 | hypothetical LOC552876 |
| 1422863_s_at | 1.69 | Pdlim5 | PDZ and LIM domain 5 |
| 1428271_at | 1.72 | Acbd4 | acyl-Coenzyme A binding domain containing 4 |
| 1422078_at | 1.72 | Akt3 | thymoma viral proto-oncogene 3 |
| 1449682_s_at | 2.48 | Tubb2b | tubulin, beta 2a, pseudogene 2 |
| 1417747_at | 1.59 | Cplx1 | complexin 1 |
| 1453051_at | 1.73 | Zkscan1 | zinc finger with KRAB and SCAN domains 1 |
| 1418189_s_at | 2.78 | Ramp2 | receptor (calcitonin) activity modifying protein 2 |
| 1427470_s_at | 3.17 | Napb | N-ethylmaleimide sensitive fusion protein attachment protein beta |
| 1456973_at | 1.70 | Arid5b | |
| 1429210_at | 1.99 | Col23a1 | collagen, type XXIII, alpha 1 |
| 1424053_a_at | 2.25 | Tcf25 | transcription factor 25 (basic helix-loop-helix) |
| 1438361_at | 1.63 | 2310035C23Rik | RIKEN cDNA 2310035C23 gene |
| 1430452_at | 2.00 | Cyp20a1 | cytochrome P450, family 20, subfamily A, polypeptide 1 |
| 1452814_at | 1.91 | Cpne3 | copine III |
| 1423777_at | 1.69 | Usp20 | ubiquitin specific peptidase 20 |
| 1419449_a_at | 2.16 | Gnai2 | guanine nucleotide binding protein (G protein), alpha inhibiting 2 |
| 1458408_at | 1.96 | Samd8 | sterile alpha motif domain containing 8 |
| 1416986_a_at | 1.69 | Sirpa | signal-regulatory protein alpha |
| 1421011_at | 2.14 | Hsd17b11 | hydroxysteroid (17-beta) dehydrogenase 11 |
| 1437545_at | 1.66 | Rcor1 | REST corepressor 1 |
| 1420947_at | 2.20 | Atrx | alpha thalassemia/mental retardation syndrome X-linked homolog (human) |
| 1443619_at | 2.34 | Tmem30a | transmembrane protein 30A |
| 1423194_at | 2.41 | Arhgap5 | Rho GTPase activating protein 5 |
| 1447481_at | 1.61 | Setd5 | |
| 1436169_at | 1.78 | C730029A08Rik | RIKEN cDNA C730029A08 gene |
| 1425911_a_at | 4.29 | Fgfr1 | fibroblast growth factor receptor 1 |
| 1435071_at | 1.54 | Zfyve1 | zinc finger, FYVE domain containing 1 |
| 1430980_a_at | 4.52 | Eif4a1 | eukaryotic translation initiation factor 4A1 |
| 1438401_at | 1.84 | Ubn1 | ubinuclein 1 |
| 1415801_at | 4.10 | Gja1 | gap junction protein, alpha 1 |
| 1421916_at | 1.97 | Pdgfra | platelet derived growth factor receptor, alpha polypeptide |
| 1448469_at | 2.15 | Nid1 | nidogen 1 |
| 1459009_at | 2.35 | Utrn | |
| 1434282_at | 2.01 | Ibtk | inhibitor of Bruton agammaglobulinemia tyrosine kinase |
| 1459635_at | 1.91 | Dlg1 | |
| 1456316_a_at | 1.82 | Acbd3 | acyl-Coenzyme A binding domain containing 3 |
| 1421954_at | 1.55 | Crkl | v-crk sarcoma virus CT10 oncogene homolog (avian)-like |
| 1425273_s_at | 1.65 | Emp2 | epithelial membrane protein 2 |
| 1420506_a_at | 2.14 | Stxbp1 | syntaxin binding protein 1 |
| 1447064_at | 2.48 | | |
| 1457214_at | 1.69 | | |
| 1451411_at | 1.67 | Gprc5b | G protein-coupled receptor, family C, group 5, member B |
| 1448155_at | 1.96 | Pdcd6ip | programmed cell death 6 interacting protein |
| 1416180_a_at | 2.70 | Rdx | radixin |
| 1426081_a_at | 1.67 | Dio2 | deiodinase, iodothyronine, type II |
| 1452187_at | 2.65 | Rbm5 | RNA binding motif protein 5 |
| 1431691_a_at | 1.59 | Rab31 | RAB31, member RAS oncogene family |

| | | | |
|---|---|---|---|
| 1450027_at | 1.77 | Sdc3 | syndecan 3 |
| 1453688_at | 1.80 | Cwf19l2 | CWF19-like 2, cell cycle control (S. pombe) |
| 1447567_at | 1.77 | Odz3 | odd Oz/ten-m homolog 3 (Drosophila) |
| 1451285_at | 4.12 | Fus | fusion, derived from t(12;16) malignant liposarcoma (human) |
| 1439293_at | 1.69 | BC031353 | cDNA sequence BC031353 |
| 1457175_at | 1.53 | Numb | |
| 1416104_at | 1.93 | Mpdu1 | mannose-P-dolichol utilization defect 1 |
| 1423578_at | 2.38 | Col11a2 | collagen, type XI, alpha 2 |
| 1424378_at | 2.16 | Ldlrap1 | low density lipoprotein receptor adaptor protein 1 |
| 1458618_at | 1.98 | Ireb2 | |
| 1450522_a_at | 2.09 | H1f0 | H1 histone family, member 0 |
| 1449264_at | 2.94 | Syt11 | synaptotagmin XI |
| 1419191_at | 1.58 | Hipk3 | homeodomain interacting protein kinase 3 |
| 1443160_at | 1.73 | Sbf2 | SET binding factor 2 |
| 1419283_s_at | 1.82 | Tns1 | tensin 1 |
| 1454664_a_at | 2.20 | Eif5 | eukaryotic translation initiation factor 5 |
| 1426653_at | 2.45 | Mcm3 | minichromosome maintenance deficient 3 (S. cerevisiae) |
| 1452497_a_at | 2.11 | Nfatc3 | nuclear factor of activated T-cells, cytoplasmic, calcineurin-dependent 3 |
| 1421862_a_at | 2.44 | Vamp1 | vesicle-associated membrane protein 1 |
| 1454683_at | 1.76 | Sfrs8 | splicing factor, arginine/serine-rich 8 |
| 1424271_at | 2.23 | Dclk1 | doublecortin-like kinase 1 |
| 1424112_at | 2.22 | Igf2r | insulin-like growth factor 2 receptor |
| 1426543_x_at | 2.12 | Endod1 | endonuclease domain containing 1 |
| 1430604_a_at | 1.57 | Dab2 | disabled homolog 2 (Drosophila) |
| 1442992_at | 2.07 | 130004C03 | hypothetical LOC403343 |
| 1417416_at | 2.19 | Kcna1 | potassium voltage-gated channel, shaker-related subfamily, member 1 |
| 1452308_a_at | 3.27 | Atp1a2 | ATPase, Na+/K+ transporting, alpha 2 polypeptide |
| 1422512_a_at | 1.79 | Ogfr | opioid growth factor receptor |
| 1424398_at | 3.06 | Dhx36 | DEAH (Asp-Glu-Ala-His) box polypeptide 36 |
| 1416933_at | 1.55 | Por | P450 (cytochrome) oxidoreductase |
| 1450354_a_at | 2.03 | Ptdss2 | phosphatidylserine synthase 2 |
| 1449054_a_at | 2.97 | Pcbp4 | poly(rC) binding protein 4 |
| 1422272_at | 1.59 | Phxr4 | per-hexamer repeat gene 4 |
| 1448880_at | 1.72 | Ube2l3 | ubiquitin-conjugating enzyme E2L 3 |
| 1422779_at | 3.56 | Smpd3 | sphingomyelin phosphodiesterase 3, neutral |
| 1440609_at | 1.58 | Map4k4 | mitogen-activated protein kinase kinase kinase kinase 4 |
| 1426310_at | 1.64 | Zdhhc5 | zinc finger, DHHC domain containing 5 |
| 1425679_a_at | 1.98 | Mapk8ip1 | mitogen-activated protein kinase 8 interacting protein 1 |
| 1427139_at | 1.60 | Adamts10 | a disintegrin-like and metallopeptidase (reprolysin type) with thrombospondin type 1 motif, 10 |
| 1448573_a_at | -1.62 | Ceacam10 | carcinoembryonic antigen-related cell adhesion molecule 10 |
| 1449341_a_at | 1.71 | Stom | stomatin |
| 1427037_at | 1.63 | Eif4g1 | eukaryotic translation initiation factor 4, gamma 1 |
| 1424325_at | 1.51 | Esco1 | establishment of cohesion 1 homolog 1 (S. cerevisiae) |
| 1415877_at | 1.87 | Dpysl3 | dihydropyrimidinase-like 3 |
| 1450074_at | 1.65 | Kif3b | kinesin family member 3B |
| 1430561_at | 1.83 | Dnajb14 | DnaJ (Hsp40) homolog, subfamily B, member 14 |
| 1459729_at | 1.92 | Slc13a5 | solute carrier family 13 (sodium-dependent citrate transporter), member 5 |
| 1451458_at | 2.96 | Tmem2 | transmembrane protein 2 |
| 1424931_s_at | -1.54 | Igl-V1 | immunoglobulin lambda chain, variable 1 |
| 1429882_at | 2.09 | 6820431F20Rik | RIKEN cDNA 6820431F20 gene |
| 1421044_at | 1.86 | Mrc2 | mannose receptor, C type 2 |
| 1432027_a_at | 1.54 | Tbc1d14 | TBC1 domain family, member 14 |
| 1433147_at | 1.74 | Cald1 | caldesmon 1 |
| 1426587_a_at | 1.63 | Stat3 | signal transducer and activator of transcription 3 |
| 1450480_a_at | 1.57 | Grk6 | G protein-coupled receptor kinase 6 |

| | | | |
|---|---|---|---|
| 1444175_at | 1.81 | Arfgef1 | |
| 1442061_at | 1.54 | Btbd7 | BTB (POZ) domain containing 7 |
| 1434567_at | 1.71 | 4732496O08Rik | RIKEN cDNA 4732496O08 gene |
| 1416159_at | 1.90 | Nr2f2 | nuclear receptor subfamily 2, group F, member 2 |
| 1425465_a_at | 1.78 | Senp2 | SUMO/sentrin specific peptidase 2 |
| 1423598_at | 1.84 | Atp8a1 | ATPase, aminophospholipid transporter (APLT), class I, type 8A, member 1 |
| 1423594_a_at | 1.79 | Ednrb | endothelin receptor type B |
| 1423597_at | 2.00 | Atp8a1 | ATPase, aminophospholipid transporter (APLT), class I, type 8A, member 1 |
| 1451121_a_at | 1.94 | Gltscr2 | glioma tumor suppressor candidate region gene 2 |
| 1456255_at | 1.86 | AI314180 | expressed sequence AI314180 |
| 1455548_at | 1.96 | Dlgap4 | discs, large homolog-associated protein 4 (Drosophila) |
| 1439686_at | 2.87 | | |
| 1448404_at | 2.23 | Scamp2 | secretory carrier membrane protein 2 |
| 1425263_a_at | 1.61 | Mbp | myelin basic protein |
| 1434566_a_at | 1.65 | 4732496O08Rik | RIKEN cDNA 4732496O08 gene |
| 1450965_at | 1.80 | Tex261 | testis expressed gene 261 |
| 1442126_at | 1.60 | 5830417C01Rik | |
| 1422799_at | 1.89 | Bat2 | HLA-B associated transcript 2 |
| 1451920_a_at | 2.05 | Rfc1 | replication factor C (activator 1) 1 |
| 1446548_at | 1.58 | Gltscr1 | |
| 1459747_at | 2.40 | | |
| 1452137_at | 2.42 | Acbd3 | acyl-Coenzyme A binding domain containing 3 |
| 1451577_at | 1.90 | Zbtb20 | zinc finger and BTB domain containing 20 |
| 1418452_at | 2.20 | Gng2 | guanine nucleotide binding protein (G protein), gamma 2 |
| 1448596_at | 1.58 | Slc6a8 | solute carrier family 6 (neurotransmitter transporter, creatine), member 8 |
| 1453224_at | 1.55 | Zfand5 | zinc finger, AN1-type domain 5 |
| 1421922_at | 1.72 | Sh3bp5 | SH3-domain binding protein 5 (BTK-associated) |
| 1438590_at | 1.80 | Rapgef3 | Rap guanine nucleotide exchange factor (GEF) 3 |
| 1426118_a_at | 1.60 | Tomm40 | translocase of outer mitochondrial membrane 40 homolog (yeast) |
| 1455033_at | 1.67 | B430201A12Rik | RIKEN cDNA B430201A12 gene |
| 1451251_at | 1.64 | Appbp2 | amyloid beta precursor protein (cytoplasmic tail) binding protein 2 |
| 1427353_at | 2.17 | Clasp1 | CLIP associating protein 1 |
| 1453836_a_at | 3.06 | Mgll | monoglyceride lipase |
| 1424263_at | 2.02 | 2810003C17Rik | RIKEN cDNA 2810003C17 gene |
| 1457343_at | 1.74 | Strn3 | |
| 1449055_x_at | 1.63 | Pcbp4 | poly(rC) binding protein 4 |
| 1447649_x_at | 1.52 | Dnajc1 | DnaJ (Hsp40) homolog, subfamily C, member 1 |
| 1423652_at | 1.72 | Isca1 | iron-sulfur cluster assembly 1 homolog (S. cerevisiae) |
| 1452742_at | 1.52 | Trak1 | trafficking protein, kinesin binding 1 |
| 1436713_s_at | 2.89 | Meg3 | maternally expressed 3 |
| 1442834_at | 1.59 | Ppp4r2 | |
| 1460288_a_at | 1.79 | Ppp4c | protein phosphatase 4, catalytic subunit |
| 1426259_at | 1.94 | Pank3 | pantothenate kinase 3 |
| 1450732_a_at | 1.96 | Bicd2 | bicaudal D homolog 2 (Drosophila) |
| 1427950_at | 1.51 | Zfp294 | zinc finger protein 294 |
| 1436923_at | 1.71 | Rab2b | RAB2B, member RAS oncogene family |
| 1426366_at | 2.23 | Eif2c2 | eukaryotic translation initiation factor 2C, 2 |
| 1419031_at | 2.58 | Fads2 | fatty acid desaturase 2 |
| 1452115_a_at | 1.53 | Plk4 | polo-like kinase 4 (Drosophila) |
| 1424187_at | 1.78 | Ccdc80 | coiled-coil domain containing 80 |
| 1443522_s_at | 2.09 | Phip | pleckstrin homology domain interacting protein |
| 1448747_at | 1.88 | Fbxo32 | F-box protein 32 |
| 1449615_s_at | 2.07 | Hdlbp | high density lipoprotein (HDL) binding protein |
| 1418533_s_at | 1.83 | Fzd2 | frizzled homolog 2 (Drosophila) |
| 1417084_at | 2.28 | Eif4ebp2 | eukaryotic translation initiation factor 4E binding protein 2 |

| | | | |
|---|---|---|---|
| 1450687_at | -1.51 | Igf2bp3 | insulin-like growth factor 2 mRNA binding protein 3 |
| 1436961_at | 1.74 | Hspa12a | heat shock protein 12A |
| 1415823_at | 5.41 | Scd2 | stearoyl-Coenzyme A desaturase 2 |
| 1421064_at | 1.55 | Mpp5 | membrane protein, palmitoylated 5 (MAGUK p55 subfamily member 5) |
| 1456979_at | 1.96 | Zhx3 | |
| 1451794_at | 2.55 | Tmcc3 | transmembrane and coiled coil domains 3 |
| 1421809_at | 1.73 | Dgcr2 | DiGeorge syndrome critical region gene 2 |
| 1428721_at | 2.08 | Klhl28 | kelch-like 28 (Drosophila) |
| 1458439_a_at | 1.59 | Dzip3 | DAZ interacting protein 3, zinc finger |
| 1420868_s_at | 2.63 | Tmed2 | transmembrane emp24 domain trafficking protein 2 |
| 1456357_at | 1.72 | A930041I02Rik | RIKEN cDNA A930041I02 gene |
| 1458050_at | 1.68 | | |
| 1449134_s_at | -1.61 | Spic | Spi-C transcription factor (Spi-1/PU.1 related) |
| 1436858_at | 3.55 | Mbnl2 | muscleblind-like 2 |
| 1427478_at | 1.51 | Usp12 | ubiquitin specific peptidase 12 |
| 1423630_at | 1.66 | Cygb | cytoglobin |
| 1452360_a_at | 2.25 | Jarid1a | jumonji, AT rich interactive domain 1A (Rbp2 like) |
| 1427293_a_at | 2.14 | Auts2 | autism susceptibility candidate 2 |
| 1418020_s_at | 3.27 | Cpd | carboxypeptidase D |
| 1436023_at | 2.00 | Bclaf1 | BCL2-associated transcription factor 1 |
| 1417236_at | 1.54 | Ehd3 | EH-domain containing 3 |
| 1455831_at | 1.84 | Fus | fusion, derived from t(12;16) malignant liposarcoma (human) |
| 1422024_at | 1.62 | Fli1 | Friend leukemia integration 1 |
| 1430392_at | 1.58 | 9530086O07Rik | RIKEN cDNA 9530086O07 gene |
| 1419088_at | 1.50 | Timp3 | tissue inhibitor of metalloproteinase 3 |
| 1442470_at | 1.60 | | |
| 1453724_a_at | 2.95 | Serpinf1 | serine (or cysteine) peptidase inhibitor, clade F, member 1 |
| 1449846_at | -2.80 | Ear2 | eosinophil-associated, ribonuclease A family, member 2 |
| 1442083_at | 2.33 | Rsrc2 | arginine/serine-rich coiled-coil 2 |
| 1422990_at | 1.97 | Met | met proto-oncogene |
| 1425539_a_at | 2.34 | Rtn3 | reticulon 3 |
| 1426346_at | 1.84 | Prepl | prolyl endopeptidase-like |
| 1452444_at | 2.97 | Napb | N-ethylmaleimide sensitive fusion protein attachment protein beta |
| 1417153_at | 1.85 | Btbd14a | BTB (POZ) domain containing 14A |
| 1420653_at | 2.61 | Tgfb1 | transforming growth factor, beta 1 |
| 1455840_at | 1.80 | Rapgef5 | Rap guanine nucleotide exchange factor (GEF) 5 |
| 1450494_x_at | 1.51 | Ceacam1 | carcinoembryonic antigen-related cell adhesion molecule 1 |
| 1444746_at | 3.10 | Ptbp2 | |
| 1436830_at | 1.51 | Marveld1 | MARVEL (membrane-associating) domain containing 1 |
| 1451069_at | 1.58 | Pim3 | proviral integration site 3 |
| 1448301_s_at | -1.72 | Serpinb1a | serine (or cysteine) peptidase inhibitor, clade B, member 1a |
| 1457491_at | 1.55 | Plekha1 | pleckstrin homology domain containing, family A (phosphoinositide binding specific) member 1 |
| 1456386_at | 3.01 | Rbm39 | |
| 1444140_at | 1.53 | | |
| 1439075_at | 2.17 | Polr3f | polymerase (RNA) III (DNA directed) polypeptide F |
| 1457262_at | 1.53 | 2610207I05Rik | RIKEN cDNA 2610207I05 gene |
| 1423554_at | 2.14 | Ggcx | gamma-glutamyl carboxylase |
| 1424922_a_at | 1.82 | Brd4 | bromodomain containing 4 |
| 1438362_x_at | 1.50 | 2310035C23Rik | RIKEN cDNA 2310035C23 gene |
| 1421144_at | -2.20 | Rpgrip1 | retinitis pigmentosa GTPase regulator interacting protein 1 |
| 1415811_at | 2.54 | Uhrf1 | ubiquitin-like, containing PHD and RING finger domains, 1 |
| 1422164_at | 2.03 | Pou3f4 | POU domain, class 3, transcription factor 4 |
| 1425487_at | 2.42 | Slu7 | SLU7 splicing factor homolog (S. cerevisiae) |
| 1418441_at | 3.09 | Col8a1 | collagen, type VIII, alpha 1 |
| 1432007_s_at | 1.58 | Ap2a2 | adaptor protein complex AP-2, alpha 2 subunit |

| | | | |
|---|---|---|---|
| 1423743_at | 1.70 | Arcn1 | archain 1 |
| 1451658_a_at | 1.50 | Polr3c | polymerase (RNA) III (DNA directed) polypeptide C |
| 1417343_at | 1.80 | Fxyd6 | FXYD domain-containing ion transport regulator 6 |
| 1451691_at | 2.22 | Ednra | endothelin receptor type A |
| 1454106_a_at | 1.53 | Cxxc1 | CXXC finger 1 (PHD domain) |
| 1442483_at | 2.00 | Fut8 | |
| 1448945_at | 1.75 | Pllp | plasma membrane proteolipid |
| 1421187_at | 1.75 | Ccr2 | chemokine (C-C motif) receptor 2 |
| 1435355_at | -2.31 | Neb | nebulin |
| 1437633_at | 1.75 | Ankrd11 | ankyrin repeat domain 11 |
| 1434480_at | 1.81 | 4930402E16Rik | RIKEN cDNA 4930402E16 gene |
| 1422528_a_at | 2.87 | Zfp36l1 | zinc finger protein 36, C3H type-like 1 |
| 1418768_at | 1.53 | Opa1 | optic atrophy 1 homolog (human) |
| 1452806_at | 2.46 | 1500016O10Rik | RIKEN cDNA 1500016O10 gene |
| 1456054_a_at | 1.72 | Pum1 | pumilio 1 (Drosophila) |
| 1448908_at | 2.76 | Ppap2b | phosphatidic acid phosphatase type 2B |
| 1448414_at | 2.25 | Rad1 | RAD1 homolog (S. pombe) |
| 1453054_at | 1.71 | Scamp1 | secretory carrier membrane protein 1 |
| 1442939_at | 1.57 | Rif1 | Rap1 interacting factor 1 homolog (yeast) |
| 1434106_at | 3.01 | Epm2aip1 | EPM2A (laforin) interacting protein 1 |
| 1427967_at | 1.77 | Cdk5rap2 | CDK5 regulatory subunit associated protein 2 |
| 1449548_at | 1.53 | Efnb2 | ephrin B2 |
| 1431197_at | 1.86 | Arl6ip2 | ADP-ribosylation factor-like 6 interacting protein 2 |
| 1431052_at | 2.24 | Arhgap12 | Rho GTPase activating protein 12 |
| 1430332_a_at | 2.04 | Gusb | glucuronidase, beta |
| 1456923_at | 2.71 | Trpm3 | transient receptor potential cation channel, subfamily M, member 3 |
| 1458056_at | 2.17 | Sfrs12 | splicing factor, arginine/serine-rich 12 |
| 1437244_at | 2.38 | Gas2l3 | growth arrest-specific 2 like 3 |
| 1426485_at | 1.79 | Ubxd2 | UBX domain protein 4 |
| 1445758_at | 1.63 | | |
| 1417502_at | 3.39 | Tspan7 | tetraspanin 7 |
| 1446536_at | 1.73 | Sema6d | sema domain, transmembrane domain (TM), and cytoplasmic domain, (semaphorin) 6D |
| 1450085_at | 1.68 | Angptl2 | angiopoietin-like 2 |
| 1456112_at | 2.03 | Tpr | translocated promoter region |
| 1431218_at | 1.65 | Zdhhc20 | zinc finger, DHHC domain containing 20 |
| 1424307_at | 1.52 | Arhgap1 | Rho GTPase activating protein 1 |
| 1440083_at | 1.53 | A430061O12Rik | RIKEN cDNA A430061O12 gene |
| 1460328_at | 1.61 | Brd3 | bromodomain containing 3 |
| 1438824_at | 2.41 | Slc20a1 | solute carrier family 20, member 1 |
| 1427430_at | 2.60 | AI848100 | expressed sequence AI848100 |
| 1416525_at | 3.61 | Spop | speckle-type POZ protein |
| 1416160_at | 2.04 | Nr2f2 | nuclear receptor subfamily 2, group F, member 2 |
| 1451109_a_at | 1.76 | Nedd4 | neural precursor cell expressed, developmentally down-regulated 4 |
| 1425529_s_at | 1.71 | D19Wsu162e | DNA segment, Chr 19, Wayne State University 162, expressed |
| 1448818_at | 1.79 | Wnt5a | wingless-related MMTV integration site 5A |
| 1438719_at | 1.68 | Map3k2 | mitogen-activated protein kinase kinase kinase 2 |
| 1449136_at | -2.57 | Epx | eosinophil peroxidase |
| 1457338_at | 2.04 | Ppp1r12b | |
| 1431314_a_at | 1.60 | 5830417I10Rik | RIKEN cDNA 5830417I10 gene |
| 1431465_s_at | 1.64 | Fyttd1 | forty-two-three domain containing 1 |
| 1450384_at | 1.85 | Bace1 | beta-site APP cleaving enzyme 1 |
| 1441448_at | 1.79 | Pum2 | pumilio 2 (Drosophila) |
| 1436434_at | 2.06 | E2f2 | E2F transcription factor 2 |
| 1429123_at | 1.78 | Rab27a | RAB27A, member RAS oncogene family |
| 1458222_at | 1.90 | | |

| | | | |
|---|---|---|---|
| 1416726_s_at | 1.92 | Ube2s | ubiquitin-conjugating enzyme E2S |
| 1415798_at | 1.54 | Ddr1 | discoidin domain receptor family, member 1 |
| 1440013_at | 1.54 | Trim44 | |
| 1418292_at | 2.48 | Asna1 | arsA arsenite transporter, ATP-binding, homolog 1 (bacterial) |
| 1417359_at | 1.77 | Mfap2 | microfibrillar-associated protein 2 |
| 1459485_at | 1.64 | Neo1 | |
| 1436968_x_at | 1.78 | Klhl24 | kelch-like 24 (Drosophila) |
| 1432558_a_at | 2.27 | Mal | myelin and lymphocyte protein, T-cell differentiation protein |
| 1442837_at | 2.33 | | |
| 1419534_at | -1.53 | Olr1 | oxidized low density lipoprotein (lectin-like) receptor 1 |
| 1427058_at | 2.41 | Eif4a1 | eukaryotic translation initiation factor 4A1 |
| 1421381_a_at | 1.85 | Col9a1 | collagen, type IX, alpha 1 |
| 1430996_at | 2.13 | Etnk1 | ethanolamine kinase 1 |
| 1459917_at | 1.70 | Ggnbp2 | gametogenetin binding protein 2 |
| 1452418_at | 1.89 | 1200016E24Rik | RIKEN cDNA 1200016E24 gene |
| 1456506_at | 1.65 | Prpf38b | PRP38 pre-mRNA processing factor 38 (yeast) domain containing B |
| AFFX-b-ActinMur/M12481_M_at | 1.95 | Actb | actin, beta |
| 1431293_a_at | 1.53 | Cldnd1 | claudin domain containing 1 |
| 1440573_at | 2.10 | Erbb2ip | predicted gene, 100040086 |
| 1422455_s_at | 1.58 | Nsf | N-ethylmaleimide sensitive fusion protein |
| 1422412_x_at | -1.90 | Ear3 | eosinophil-associated, ribonuclease A family, member 3 |
| 1436706_at | 2.56 | Tmem32 | transmembrane protein 32 |
| 1421027_a_at | 1.57 | Mef2c | myocyte enhancer factor 2C |
| 1427604_a_at | 2.60 | Atp9a | ATPase, class II, type 9A |
| 1441352_at | 1.67 | Tor1aip1 | torsin A interacting protein 1 |
| 1419102_at | 1.82 | Sin3a | transcriptional regulator, SIN3A (yeast) |
| 1456659_at | 1.70 | LOC552902 | hypothetical LOC552902 |
| 1440440_at | 1.57 | | |
| 1423974_at | 1.58 | Numa1 | nuclear mitotic apparatus protein 1 |
| 1456983_at | 1.57 | Trps1 | |
| 1453222_at | 1.78 | Gse1 | genetic suppressor element 1 |
| 1443904_at | 2.41 | Fads6 | fatty acid desaturase domain family, member 6 |
| 1446500_at | 1.56 | Mef2a | |
| 1439158_at | 2.13 | Tlk1 | tousled-like kinase 1 |
| 1439035_at | 1.77 | Zfp322a | zinc finger protein 322a |
| 1444811_at | 1.70 | Tloc1 | SEC62 homolog (S. cerevisiae) |
| 1456048_at | 1.55 | Cpeb3 | cytoplasmic polyadenylation element binding protein 3 |
| 1424620_at | 1.70 | D13Wsu177e | DNA segment, Chr 13, Wayne State University 177, expressed |
| 1456993_at | 1.71 | D2Ertd640e | DNA segment, Chr 2, ERATO Doi 640, expressed |
| 1451348_at | 1.87 | Depdc6 | DEP domain containing 6 |
| 1427773_a_at | 2.30 | Rabac1 | Rab acceptor 1 (prenylated) |
| 1448780_at | 1.83 | Slc12a2 | solute carrier family 12, member 2 |
| 1460336_at | 1.75 | Ppargc1a | peroxisome proliferative activated receptor, gamma, coactivator 1 alpha |
| 1430498_at | 2.14 | 9130009I01Rik | RIKEN cDNA 9130009I01 gene |
| 1426541_a_at | 2.97 | Endod1 | endonuclease domain containing 1 |
| 1421233_at | 2.71 | Pknox1 | Pbx/knotted 1 homeobox |
| 1417355_at | 1.70 | Peg3 | paternally expressed 3 |
| 1443002_at | 1.68 | Zfr | zinc finger RNA binding protein |
| 1435129_at | 1.97 | Ptp4a2 | |
| 1437110_at | 2.73 | 2810474O19Rik | RIKEN cDNA 2810474O19 gene |
| 1437002_at | 1.51 | C030011O14Rik | RIKEN cDNA C030011O14 gene |
| 1449028_at | 2.02 | Rhou | ras homolog gene family, member U |
| 1423716_s_at | 1.57 | Atp5d | ATP synthase, H+ transporting, mitochondrial F1 complex, delta subunit |
| 1458701_at | 1.66 | Prei4 | preimplantation protein 4 |
| 1449066_a_at | 1.55 | Arhgef7 | Rho guanine nucleotide exchange factor (GEF7) |

| | | | |
|---|---|---|---|
| 1422411_s_at | -2.71 | Ear3 | eosinophil-associated, ribonuclease A family, member 3 |
| 1453841_at | 1.52 | 2310050P20Rik | RIKEN cDNA 2310050P20 gene |
| 1450035_a_at | 1.83 | Prpf40a | PRP40 pre-mRNA processing factor 40 homolog A (yeast) |
| 1439606_at | 1.85 | | |
| 1421802_at | -3.44 | Ear1 | eosinophil-associated, ribonuclease A family, member 1 |
| 1440553_at | 1.64 | Mecr | mitochondrial trans-2-enoyl-CoA reductase |
| 1456781_at | 1.55 | | |
| 1423250_a_at | 1.81 | Tgfb2 | transforming growth factor, beta 2 |
| 1439087_a_at | 2.55 | 5830455E04Rik | RIKEN cDNA 5830455E04 gene |
| 1438702_at | 1.76 | Flrt2 | fibronectin leucine rich transmembrane protein 2 |
| 1435909_at | -1.61 | C030034I22Rik | RIKEN cDNA C030034I22 gene |
| 1440771_at | 1.56 | Zkscan1 | zinc finger with KRAB and SCAN domains 1 |
| 1433916_at | 1.71 | Vamp3 | vesicle-associated membrane protein 3 |
| 1457047_at | 1.50 | | |
| 1436722_a_at | 1.51 | Actb | actin, beta |
| 1421348_a_at | 2.20 | Cend1 | cell cycle exit and neuronal differentiation 1 |
| 1441354_at | 1.63 | AU018740 | expressed sequence AU018740 |
| 1425538_x_at | 1.73 | Ceacam1 | carcinoembryonic antigen-related cell adhesion molecule 1 |
| 1442886_at | 1.53 | Tra2a | |
| 1455642_a_at | 1.50 | Tspan17 | tetraspanin 17 |
| 1436892_at | 1.77 | Spred2 | sprouty-related, EVH1 domain containing 2 |
| 1425480_at | 1.90 | Cnot6l | CCR4-NOT transcription complex, subunit 6-like |
| 1421378_s_at | 1.90 | Abcc1 | ATP-binding cassette, sub-family C (CFTR/MRP), member 1 |
| 1450440_at | 1.92 | Gfra1 | glial cell line derived neurotrophic factor family receptor alpha 1 |
| 1427225_at | 2.04 | Epn2 | epsin 2 |
| 1456947_at | 1.63 | Pafah1b1 | |
| 1458847_at | 2.07 | | |
| 1439129_at | 1.54 | Dock5 | dedicator of cytokinesis 5 |
| 1420948_s_at | 2.93 | Atrx | alpha thalassemia/mental retardation syndrome X-linked homolog (human) |
| 1425522_at | 1.91 | Rbm25 | RNA binding motif protein 25 |
| 1415957_a_at | 3.14 | Rrp1 | ribosomal RNA processing 1 homolog (S. cerevisiae) |
| 1430829_s_at | 1.63 | Fto | fat mass and obesity associated |
| 1441326_at | 1.75 | Cp | ceruloplasmin |
| 1416738_at | 1.51 | Brap | BRCA1 associated protein |
| 1460047_at | 1.72 | Fnbp1 | |
| 1437657_at | 1.59 | Scaper | S phase cyclin A-associated protein in the ER |
| 1444087_at | 1.53 | Prpf38a | PRP38 pre-mRNA processing factor 38 (yeast) domain containing A |
| 1416250_at | 1.91 | Btg2 | B-cell translocation gene 2, anti-proliferative |
| 1421349_x_at | 2.88 | Cend1 | cell cycle exit and neuronal differentiation 1 |
| 1425296_a_at | 1.54 | Rgs3 | regulator of G-protein signaling 3 |
| 1458994_at | 2.01 | Csnk1g3 | |
| 1418892_at | 2.09 | Rhoj | ras homolog gene family, member J |
| 1430981_s_at | 3.51 | Gpbp1 | GC-rich promoter binding protein 1 |
| 1448200_at | 1.58 | Tcn2 | transcobalamin 2 |
| 1444066_at | 1.52 | Gapvd1 | GTPase activating protein and VPS9 domains 1 |
| 1438268_at | 1.50 | Rc3h2 | ring finger and CCCH-type zinc finger domains 2 |
| 1431606_a_at | 2.87 | Angel2 | angel homolog 2 (Drosophila) |
| 1438735_at | 1.51 | Rsf1 | remodeling and spacing factor 1 |
| 1423432_at | 1.71 | Phip | pleckstrin homology domain interacting protein |
| 1434643_at | 1.58 | Tbl1x | transducin (beta)-like 1 X-linked |
| 1454011_a_at | 2.53 | Rpa2 | replication protein A2 |
| 1447143_at | 1.69 | Phf14 | |
| 1443390_at | 1.69 | Dock1 | |
| 1449219_at | 1.60 | Fads3 | fatty acid desaturase 3 |
| 1421727_at | 1.76 | Eya1 | eyes absent 1 homolog (Drosophila) |

| | | | |
|---|---|---|---|
| 1451119_a_at | 2.19 | Fbln1 | fibulin 1 |
| 1443675_at | 1.50 | Epc1 | |
| 1448605_at | 2.47 | Rhoc | ras homolog gene family, member C |
| 1450051_at | 2.18 | Atrx | alpha thalassemia/mental retardation syndrome X-linked homolog (human) |
| 1453163_at | 2.41 | Ppp1r12a | protein phosphatase 1, regulatory (inhibitor) subunit 12A |
| 1455980_a_at | 2.52 | Gas2l3 | growth arrest-specific 2 like 3 |
| 1441038_at | 1.57 | Utrn | |
| 1418279_a_at | 1.56 | Akap1 | A kinase (PRKA) anchor protein 1 |
| 1450038_s_at | 2.51 | Usp9x | ubiquitin specific peptidase 9, X chromosome |
| 1436221_at | 2.01 | D1Ertd471e | DNA segment, Chr 1, ERATO Doi 471, expressed |
| 1455736_at | -1.56 | Mybpc2 | myosin binding protein C, fast-type |
| 1439330_at | 1.57 | D230040J21Rik | RIKEN cDNA D230040J21 gene |
| 1415939_at | 1.52 | Fmod | fibromodulin |
| 1416835_s_at | 2.35 | Amd1 | S-adenosylmethionine decarboxylase 1 |
| 1425888_at | -1.53 | Klra17 | killer cell lectin-like receptor, subfamily A, member 17 |
| 1416188_at | 2.44 | Gm2a | GM2 ganglioside activator protein |
| 1445885_at | 1.70 | Ube2d2 | ubiquitin-conjugating enzyme E2D 2 |
| 1454721_at | 2.11 | 1110018G07Rik | RIKEN cDNA 1110018G07 gene |
| 1416228_at | 1.90 | Pin1 | protein (peptidyl-prolyl cis/trans isomerase) NIMA-interacting 1 |
| 1448510_at | 1.87 | Efna1 | ephrin A1 |
| 1434610_at | 1.85 | Plec1 | plectin 1 |
| 1416583_at | 1.54 | Bad | BCL2-associated agonist of cell death |
| 1425732_a_at | 1.56 | Mxi1 | Max interacting protein 1 |
| 1439305_at | 2.26 | | |
| 1434707_at | 1.63 | Sbf1 | SET binding factor 1 |
| 1443412_s_at | 1.53 | Mmp16 | matrix metallopeptidase 16 |
| 1431216_s_at | 1.58 | Dnajc6 | DnaJ (Hsp40) homolog, subfamily C, member 6 |
| 1444507_at | 1.53 | Usp53 | ubiquitin specific peptidase 53 |
| 1421028_a_at | 2.11 | Mef2c | myocyte enhancer factor 2C |
| 1435449_at | 1.72 | Bcl2l11 | BCL2-like 11 (apoptosis facilitator) |
| 1430151_at | 1.77 | Nisch | nischarin |
| 1445322_x_at | 1.82 | E430025E21Rik | |
| 1438040_a_at | 2.34 | Hsp90b1 | heat shock protein 90, beta (Grp94), member 1 |
| 1426710_at | 1.52 | Calm3 | calmodulin 3 |
| 1418006_at | 1.71 | Zc3h18 | zinc finger CCCH-type containing 18 |
| 1430820_a_at | 1.83 | Bbx | bobby sox homolog (Drosophila) |
| 1441370_at | 1.74 | Tmcc1 | transmembrane and coiled coil domains 1 |
| 1443728_at | 2.70 | | |
| 1418464_at | 1.70 | Matn4 | matrilin 4 |
| 1433662_s_at | 2.36 | Timp2 | tissue inhibitor of metalloproteinase 2 |
| 1421315_s_at | 2.65 | Cttn | cortactin |
| 1416801_at | 1.54 | Trpm7 | transient receptor potential cation channel, subfamily M, member 7 |
| 1421959_s_at | 1.81 | Adcy3 | adenylate cyclase 3 |
| 1453313_at | 1.50 | Sesn3 | sestrin 3 |
| 1448211_at | 1.69 | Atp6v0e2 | ATPase, H+ transporting, lysosomal V0 subunit E2 |
| 1427379_at | 1.55 | Pnpla6 | patatin-like phospholipase domain containing 6 |
| 1450537_at | 1.57 | Mid2 | midline 2 |
| 1419157_at | 2.27 | Sox4 | SRY-box containing gene 4 |
| 1438515_at | 1.66 | Zfp207 | zinc finger protein 207 |
| 1433205_at | 1.61 | 2810436B12Rik | RIKEN cDNA 2810436B12 gene |
| 1415951_at | 2.01 | Fkbp10 | FK506 binding protein 10 |
| 1425676_a_at | 2.10 | Elovl1 | elongation of very long chain fatty acids (FEN1/Elo2, SUR4/Elo3, yeast)-like 1 |
| 1427125_s_at | 1.59 | Lrrc41 | leucine rich repeat containing 41 |
| 1455757_at | 1.64 | D3Ertd254e | DNA segment, Chr 3, ERATO Doi 254, expressed |
| 1440318_at | 1.51 | Wdr35 | WD repeat domain 35 |

| | | | |
|---|---|---|---|
| 1442141_at | 1.92 | BC028454 | cDNA sequence BC028454 |
| 1435587_at | 1.83 | Pcid2 | PCI domain containing 2 |
| 1420942_s_at | 3.51 | Rgs5 | regulator of G-protein signaling 5 |
| 1458450_at | 1.82 | Zfr | zinc finger RNA binding protein |
| 1423947_at | 1.64 | 1110008P14Rik | RIKEN cDNA 1110008P14 gene |
| 1460295_s_at | 2.96 | Il6st | interleukin 6 signal transducer |
| 1451897_a_at | 2.28 | Nbr1 | neighbor of Brca1 gene 1 |
| 1425505_at | 2.01 | Mylk | myosin, light polypeptide kinase |
| 1436833_x_at | 2.02 | Ttll1 | tubulin tyrosine ligase-like 1 |
| 1451839_a_at | 2.24 | Pde7a | phosphodiesterase 7A |
| 1430533_a_at | 3.98 | Ctnnb1 | catenin (cadherin associated protein), beta 1 |
| 1437207_at | 1.75 | Rnf170 | ring finger protein 170 |
| 1439095_at | 1.52 | Sfrs11 | splicing factor, arginine/serine-rich 11 |
| 1434644_at | 2.51 | Tbl1x | transducin (beta)-like 1 X-linked |
| 1439832_at | 2.55 | Wasl | |
| 1450674_at | 1.66 | Cdk5 | cyclin-dependent kinase 5 |
| 1428877_at | 1.57 | Srp72 | signal recognition particle 72 |
| 1459894_at | 1.79 | Iqgap2 | IQ motif containing GTPase activating protein 2 |
| 1425188_s_at | 2.23 | Sel1l | sel-1 suppressor of lin-12-like (C. elegans) |
| 1427306_at | -1.58 | Ryr1 | ryanodine receptor 1, skeletal muscle |
| 1452893_s_at | 1.99 | 2310040A07Rik | RIKEN cDNA 2310040A07 gene |
| 1416741_at | 2.74 | Col5a1 | collagen, type V, alpha 1 |
| 1432294_at | 1.67 | 9330177L23Rik | RIKEN cDNA 9330177L23 gene |
| 1422748_at | 2.08 | Zeb2 | zinc finger E-box binding homeobox 2 |
| 1424663_at | 1.95 | BC017647 | cDNA sequence BC017647 |
| 1427391_a_at | 1.70 | Col12a1 | collagen, type XII, alpha 1 |
| 1460635_at | 1.62 | Fastk | Fas-activated serine/threonine kinase |
| 1436910_at | 2.12 | Rasal2 | RAS protein activator like 2 |
| 1451485_at | 1.65 | 3300001P08Rik | RIKEN cDNA 3300001P08 gene |
| 1426641_at | 2.86 | Trib2 | tribbles homolog 2 (Drosophila) |
| 1416317_a_at | 1.54 | Stambp | Stam binding protein |
| 1430514_a_at | 2.06 | Cd99 | CD99 antigen |
| 1449302_at | 1.60 | Abca2 | ATP-binding cassette, sub-family A (ABC1), member 2 |
| 1436519_a_at | 1.72 | 1110057K04Rik | RIKEN cDNA 1110057K04 gene |
| 1427526_at | 2.06 | Fgfr1op2 | FGFR1 oncogene partner 2 |
| 1417664_a_at | 2.06 | Ndrg3 | N-myc downstream regulated gene 3 |
| 1439122_at | 2.46 | Ddx6 | DEAD (Asp-Glu-Ala-Asp) box polypeptide 6 |
| 1437497_a_at | 2.38 | Hsp90aa1 | heat shock protein 90, alpha (cytosolic), class A member 1 |
| 1455026_at | 1.79 | Sbno1 | sno, strawberry notch homolog 1 (Drosophila) |
| 1419280_at | 2.59 | Pip4k2a | phosphatidylinositol-5-phosphate 4-kinase, type II, alpha |
| 1452170_at | 1.54 | 2010209O12Rik | RIKEN cDNA 2010209O12 gene |
| 1444020_at | 1.91 | Ncan | |
| 1449504_at | 1.59 | Kpna1 | karyopherin (importin) alpha 1 |
| 1421219_at | 1.64 | Ttll3 | tubulin tyrosine ligase-like family, member 3 |
| 1444969_at | 1.63 | 5033414K04Rik | RIKEN cDNA 5033414K04 gene |
| 1424270_at | 1.61 | Dclk1 | doublecortin-like kinase 1 |
| 1441131_at | 1.78 | | |
| 1422807_at | 1.78 | Arf5 | ADP-ribosylation factor 5 |
| AFFX-GapdhMur/M32599_M_at | 2.54 | Gapdh | glyceraldehyde-3-phosphate dehydrogenase |
| 1431230_a_at | 1.65 | Btbd9 | BTB (POZ) domain containing 9 |
| 1450846_at | 2.47 | Bzw1 | basic leucine zipper and W2 domains 1 |
| 1421839_at | 2.38 | Abca1 | ATP-binding cassette, sub-family A (ABC1), member 1 |
| 1416459_at | 2.49 | Arf2 | ADP-ribosylation factor 2 |
| 1441643_at | 2.10 | 3-Mar | membrane-associated ring finger (C3HC4) 3 |
| 1434379_at | 1.56 | Mxd4 | |

| | | | |
|---|---|---|---|
| 1423312_at | -1.52 | Tpbg | trophoblast glycoprotein |
| 1439554_at | 1.58 | Scmh1 | |
| 1447195_at | 1.54 | Elp4 | |
| 1427481_a_at | 2.68 | Atp1a3 | ATPase, Na+/K+ transporting, alpha 3 polypeptide |
| 1448144_at | 1.52 | Hnrpab | heterogeneous nuclear ribonucleoprotein A/B |
| 1437892_at | 1.52 | Zkscan3 | zinc finger with KRAB and SCAN domains 3 |
| 1428850_x_at | 2.08 | Cd99 | CD99 antigen |
| 1448901_at | 1.53 | Cpxm1 | carboxypeptidase X 1 (M14 family) |
| 1453556_x_at | 1.77 | Cd99 | CD99 antigen |
| 1425425_a_at | 1.85 | Wif1 | Wnt inhibitory factor 1 |
| 1450379_at | 3.79 | Msn | moesin |
| 1437082_at | 2.24 | Akap9 | A kinase (PRKA) anchor protein (yotiao) 9 |
| 1422264_s_at | 2.64 | Klf9 | Kruppel-like factor 9 |
| 1426667_a_at | 2.07 | Unc84a | unc-84 homolog A (C. elegans) |
| 1438714_at | 1.73 | Zfp207 | |
| 1452160_at | 2.06 | Tiparp | TCDD-inducible poly(ADP-ribose) polymerase |
| 1453727_at | 1.94 | Esf1 | ESF1, nucleolar pre-rRNA processing protein, homolog (S. cerevisiae) |
| 1421462_a_at | 1.59 | Lepre1 | leprecan 1 |
| 1458052_at | 1.50 | | |
| 1460692_at | 2.35 | Ehmt2 | euchromatic histone lysine N-methyltransferase 2 |
| 1439566_at | 1.81 | Gprin3 | GPRIN family member 3 |
| 1438739_at | 1.65 | Cnbp | |
| 1436983_at | 2.59 | Crebbp | CREB binding protein |
| 1432466_a_at | 2.15 | Apoe | apolipoprotein E |
| 1427254_at | 1.81 | Zfp445 | zinc finger protein 445 |
| 1451981_at | 2.09 | Gtrgeo22 | gene trap ROSA b-geo 22 |
| 1427385_s_at | 1.52 | Actn1 | actinin, alpha 1 |
| 1443086_at | 1.57 | Alcam | activated leukocyte cell adhesion molecule |
| 1439033_at | 2.07 | Zcchc7 | zinc finger, CCHC domain containing 7 |
| 1425686_at | 1.73 | Cflar | CASP8 and FADD-like apoptosis regulator |
| 1425338_at | 1.93 | Plcb4 | phospholipase C, beta 4 |
| 1431316_at | 1.73 | Itch | itchy, E3 ubiquitin protein ligase |
| 1432088_at | 1.73 | Veph1 | ventricular zone expressed PH domain homolog 1 (zebrafish) |
| 1443491_at | 1.70 | Ptprk | |
| 1433633_at | 2.05 | Irf2bp2 | interferon regulatory factor 2 binding protein 2 |
| 1430984_at | 2.26 | Azin1 | antizyme inhibitor 1 |
| 1425456_a_at | 2.35 | Map2k3 | mitogen-activated protein kinase kinase 3 |
| 1460741_x_at | 2.43 | D17Wsu92e | DNA segment, Chr 17, Wayne State University 92, expressed |
| 1459886_at | -1.57 | | |
| 1432411_a_at | 2.04 | Fbxw2 | F-box and WD-40 domain protein 2 |
| 1426574_a_at | 2.45 | Add3 | adducin 3 (gamma) |
| 1418155_at | -1.80 | Myot | myotilin |
| 1457357_at | 2.07 | Tlk2 | tousled-like kinase 2 (Arabidopsis) |
| 1430780_a_at | 1.97 | Pmm1 | phosphomannomutase 1 |
| 1451832_at | 1.61 | Cklf | chemokine-like factor |
| 1442365_at | 1.54 | Rtn3 | |
| 1458685_at | 1.56 | Garnl1 | GTPase activating RANGAP domain-like 1 |
| 1422746_s_at | 1.77 | Bicd2 | bicaudal D homolog 2 (Drosophila) |
| 1446606_at | -1.67 | LOC625175 | predicted gene, 625175 |
| 1416484_at | 3.22 | Ttc3 | tetratricopeptide repeat domain 3 |
| 1460256_at | 3.33 | Car3 | carbonic anhydrase 3 |
| 1425895_a_at | 1.53 | Id1 | inhibitor of DNA binding 1 |
| 1429649_at | 1.83 | Slc35a3 | solute carrier family 35 (UDP-N-acetylglucosamine (UDP-GlcNAc) transporter), member 3 |
| 1438403_s_at | 2.47 | Malat1 | metastasis associated lung adenocarcinoma transcript 1 (non-coding RNA) |
| 1442095_at | 1.56 | Asxl3 | additional sex combs like 3 (Drosophila) |

| | | | |
|---|---|---|---|
| 1421990_at | 1.53 | Syt1 | synaptotagmin I |
| 1422908_at | -1.84 | Atp1b4 | ATPase, (Na+)/K+ transporting, beta 4 polypeptide |
| 1442866_at | 1.54 | Rcsd1 | RCSD domain containing 1 |
| 1424115_at | 2.26 | Ppp5c | protein phosphatase 5, catalytic subunit |
| 1442400_at | 1.64 | Prickle1 | |
| 1416660_at | 1.74 | Eif3s10 | eukaryotic translation initiation factor 3, subunit A |
| 1444058_at | 2.00 | Dzip3 | DAZ interacting protein 3, zinc finger |
| 1449376_at | 1.92 | Nicn1 | nicolin 1 |
| 1460666_a_at | 1.58 | Ebf3 | early B-cell factor 3 |
| 1425859_a_at | 2.29 | Psmd4 | proteasome (prosome, macropain) 26S subunit, non-ATPase, 4 |
| 1430692_a_at | 2.28 | Sel1l | sel-1 suppressor of lin-12-like (C. elegans) |
| 1437210_a_at | 1.82 | Brd2 | bromodomain containing 2 |
| 1459695_at | 2.23 | | |
| 1430918_at | 1.82 | 3-Mar | membrane-associated ring finger (C3HC4) 3 |
| 1435433_at | 1.50 | Centg2 | centaurin, gamma 2 |
| 1441212_at | 1.91 | 6720467C03Rik | RIKEN cDNA 6720467C03 gene |
| 1458605_at | 1.57 | Arl15 | |
| 1450743_s_at | 2.62 | Syncrip | synaptotagmin binding, cytoplasmic RNA interacting protein |
| 1452708_a_at | 2.82 | Luc7l | Luc7 homolog (S. cerevisiae)-like |
| 1424926_at | 1.58 | Sec63 | SEC63-like (S. cerevisiae) |
| 1448346_at | 1.80 | Cfl1 | cofilin 1, non-muscle |
| 1426738_at | 1.56 | Dgkz | diacylglycerol kinase zeta |
| 1417821_at | 1.68 | D17H6S56E-5 | DNA segment, Chr 17, human D6S56E 5 |
| 1455388_at | 1.81 | Pcmtd1 | protein-L-isoaspartate (D-aspartate) O-methyltransferase domain containing 1 |
| 1426226_at | 2.02 | Dyrk1a | dual-specificity tyrosine-(Y)-phosphorylation regulated kinase 1a |
| 1439477_at | 1.58 | 5430406J06Rik | RIKEN cDNA 5430406J06 gene |
| 1435585_at | -1.81 | Tceal7 | transcription elongation factor A (SII)-like 7 |
| 1419816_s_at | 1.59 | Errfi1 | ERBB receptor feedback inhibitor 1 |
| 1427266_at | 1.88 | Pbrm1 | polybromo 1 |
| 1456398_at | 2.77 | Tug1 | taurine upregulated gene 1 |
| 1441466_at | 1.54 | Sfrs10 | splicing factor, arginine/serine-rich 10 (transformer 2 homolog, Drosophila) |
| 1450143_at | 1.56 | Rasgrp1 | RAS guanyl releasing protein 1 |
| 1417238_at | 2.21 | Ewsr1 | Ewing sarcoma breakpoint region 1 |
| 1452688_at | 1.70 | Prpf39 | PRP39 pre-mRNA processing factor 39 homolog (yeast) |
| 1428157_at | 1.75 | Gng2 | guanine nucleotide binding protein (G protein), gamma 2 |
| 1428432_at | 2.59 | 2310047A01Rik | zinc finger, CCHC domain containing 24 |
| 1425153_at | -2.25 | Myh2 | myosin, heavy polypeptide 2, skeletal muscle, adult |
| 1451241_at | 2.39 | Lamb1-1 | laminin B1 subunit 1 |
| AFFX-b-ActinMur/M12481_5_at | 3.44 | Actb | actin, beta |
| 1416311_s_at | 1.53 | Tuba3a | tubulin, alpha 3A |
| 1452030_a_at | 2.98 | Hnrnpr | heterogeneous nuclear ribonucleoprotein R |
| 1418562_at | 1.78 | Sf3b1 | splicing factor 3b, subunit 1 |
| 1425467_a_at | 2.76 | Plp1 | proteolipid protein (myelin) 1 |
| 1424345_s_at | 1.60 | Ube2m | ubiquitin-conjugating enzyme E2M (UBC12 homolog, yeast) |
| 1425581_s_at | 2.39 | Galnt7 | UDP-N-acetyl-alpha-D-galactosamine: polypeptide N-acetylgalactosaminyltransferase 7 |
| 1445068_at | 1.52 | Malt1 | mucosa associated lymphoid tissue lymphoma translocation gene 1 |
| 1450512_at | 1.60 | Ntn4 | netrin 4 |
| 1429463_at | 1.75 | Prkaa2 | protein kinase, AMP-activated, alpha 2 catalytic subunit |
| 1451350_a_at | 2.74 | Leprot | leptin receptor overlapping transcript |
| 1456677_at | 2.02 | Herc4 | |
| 1421013_at | 1.95 | Pitpnb | phosphatidylinositol transfer protein, beta |
| 1456616_a_at | 1.88 | Bsg | basigin |
| 1423557_at | 1.56 | Ifngr2 | interferon gamma receptor 2 |
| 1448119_at | 2.20 | Bpgm | 2,3-bisphosphoglycerate mutase |
| 1438265_at | 1.54 | | |

| | | | |
|---|---|---|---|
| 1457666_s_at | -1.51 | Ifi202b | interferon activated gene 202B |
| 1444693_at | 1.62 | Cacnb2 | |
| 1459332_at | 1.68 | Itch | itchy, E3 ubiquitin protein ligase |
| 1424570_at | 2.62 | Ddx46 | DEAD (Asp-Glu-Ala-Asp) box polypeptide 46 |
| 1422769_at | 2.55 | Syncrip | synaptotagmin binding, cytoplasmic RNA interacting protein |
| 1426657_s_at | 2.29 | Phgdh | 3-phosphoglycerate dehydrogenase |
| 1423117_at | 2.36 | Pum1 | pumilio 1 (Drosophila) |
| 1453960_a_at | 2.99 | Capzb | capping protein (actin filament) muscle Z-line, beta |
| 1453739_at | 1.65 | Tmem126b | transmembrane protein 126B |
| 1451056_at | 2.59 | Psmd7 | proteasome (prosome, macropain) 26S subunit, non-ATPase, 7 |
| 1417699_at | 1.85 | Gtf2f1 | general transcription factor IIF, polypeptide 1 |
| 1438533_at | 1.83 | Myo9b | myosin IXb |
| 1450080_at | 1.56 | Cxx1c | CAAX box 1 homolog C (human) |
| 1458941_at | 1.56 | Zeb2 | zinc finger E-box binding homeobox 2 |
| 1426575_at | 1.53 | Sgms1 | sphingomyelin synthase 1 |
| 1415819_a_at | 2.03 | Ppp2r1a | protein phosphatase 2 (formerly 2A), regulatory subunit A (PR 65), alpha isoform |
| 1440862_at | 1.55 | | |
| 1450377_at | 3.94 | Thbs1 | thrombospondin 1 |
| 1456256_at | 1.54 | Eif5 | eukaryotic translation initiation factor 5 |
| 1438244_at | 1.82 | Nfib | nuclear factor I/B |
| 1438556_a_at | 2.45 | Tmod3 | tropomodulin 3 |
| 1420951_a_at | 3.11 | Son | Son DNA binding protein |
| 1419064_a_at | 2.45 | Ugt8a | UDP galactosyltransferase 8A |
| 1424778_at | 2.26 | Reep3 | receptor accessory protein 3 |
| 1421832_at | 2.87 | Twsg1 | twisted gastrulation homolog 1 (Drosophila) |
| 1416348_at | 1.79 | Men1 | multiple endocrine neoplasia 1 |
| 1422910_s_at | 1.87 | Smc6 | structural maintenance of chromosomes 6 |
| 1425281_a_at | 1.69 | Tsc22d3 | TSC22 domain family, member 3 |
| 1423746_at | 2.08 | Txndc5 | thioredoxin domain containing 5 |
| 1422556_at | 2.24 | Gna13 | guanine nucleotide binding protein, alpha 13 |
| 1424768_at | 3.50 | Cald1 | caldesmon 1 |
| 1452542_x_at | 1.54 | Tcfe2a | transcription factor E2a |
| 1445048_at | 1.54 | Baz2b | bromodomain adjacent to zinc finger domain, 2B |
| 1427867_at | -1.69 | Myh1 | myosin, heavy polypeptide 1, skeletal muscle, adult |
| 1425862_a_at | 2.20 | Pik3c2a | phosphatidylinositol 3-kinase, C2 domain containing, alpha polypeptide |
| 1451782_a_at | 2.45 | Slc29a1 | solute carrier family 29 (nucleoside transporters), member 1 |
| 1453367_a_at | 2.39 | Abhd12 | abhydrolase domain containing 12 |
| 1453263_at | 2.33 | Mak10 | MAK10 homolog, amino-acid N-acetyltransferase subunit, (S. cerevisiae) |
| 1421070_at | 2.60 | D3Ertd300e | DNA segment, Chr 3, ERATO Doi 300, expressed |
| 1449660_s_at | 1.98 | Coro1c | coronin, actin binding protein 1C |

**Table S1D - Genes that are differentially regulated in a9-/- cochleas compared to wild-type controls at P60**

| Probe ID | Fold Change (log2) | Gene Symbol | Gene Description |
|---|---|---|---|
| 1447360_at | 4.42 | Tsc22d1 | TSC22 domain family, member 1 |
| 1417851_at | -3.34 | Cxcl13 | chemokine (C-X-C motif) ligand 13 |
| 1421280_at | 3.12 | Gabra1 | gamma-aminobutyric acid (GABA-A) receptor, subunit alpha 1 |
| 1421990_at | 3.09 | Syt1 | synaptotagmin I |
| 1450826_a_at | -3.03 | Saa3 | serum amyloid A 3 |
| 1442019_at | 3.01 | Rcvrn | |
| 1415824_at | 2.92 | Scd2 | stearoyl-Coenzyme A desaturase 2 |
| 1430980_a_at | 2.95 | Eif4a1 | eukaryotic translation initiation factor 4A1 |
| 1452907_at | -2.86 | Galc | galactosylceramidase |
| 1415823_at | 2.91 | Scd2 | stearoyl-Coenzyme A desaturase 2 |

| | | | |
|---|---|---|---|
| 1421063_s_at | 2.91 | Snurf | SNRPN upstream reading frame |
| 1451961_a_at | 2.80 | Mbp | myelin basic protein |
| 1458676_at | 2.76 | Nktr | natural killer tumor recognition sequence |
| 1420948_s_at | 2.76 | Atrx | alpha thalassemia/mental retardation syndrome X-linked homolog (human) |
| 1417069_a_at | 2.75 | Gmfb | glia maturation factor, beta |
| 1444333_at | 2.72 | | |
| 1431030_a_at | 2.73 | Rnf14 | ring finger protein 14 |
| 1452638_s_at | 2.71 | Dnm1l | dynamin 1-like |
| 1420899_at | 2.66 | Rab18 | RAB18, member RAS oncogene family |
| AFFX-GapdhMur/M32599_5_at | 2.67 | Gapdh | glyceraldehyde-3-phosphate dehydrogenase |
| 1415801_at | 2.60 | Gja1 | gap junction protein, alpha 1 |
| 1431686_a_at | 2.58 | Gmfb | glia maturation factor, beta |
| 1426341_at | 2.57 | Slc1a3 | solute carrier family 1 (glial high affinity glutamate transporter), member 3 |
| 1448541_at | 2.55 | Klc1 | kinesin light chain 1 |
| 1420610_at | 2.52 | Prkacb | protein kinase, cAMP dependent, catalytic, beta |
| 1451285_at | 2.57 | Fus | fusion, derived from t(12;16) malignant liposarcoma (human) |
| 1421866_at | 2.51 | Nr3c1 | nuclear receptor subfamily 3, group C, member 1 |
| 1458534_at | 2.50 | Rgs7bp | regulator of G-protein signalling 7 binding protein |
| 1456080_a_at | 2.48 | Serinc3 | serine incorporator 3 |
| 1437798_at | -2.45 | 6720422M22Rik | RIKEN cDNA 6720422M22 gene |
| 1444001_at | 2.44 | Strbp | |
| 1433515_s_at | 2.45 | Etnk1 | ethanolamine kinase 1 |
| 1416959_at | 2.41 | Nr1d2 | nuclear receptor subfamily 1, group D, member 2 |
| 1443612_at | 2.37 | | transmembrane protein 16C |
| 1451706_a_at | 2.38 | Gabra6 | gamma-aminobutyric acid (GABA-A) receptor, subunit alpha 6 |
| 1420816_at | 2.34 | Ywhag | tyrosine 3-monooxygenase/tryptophan 5-monooxygenase activation protein, gamma polypeptide |
| 1419684_at | -2.33 | Ccl8 | chemokine (C-C motif) ligand 8 |
| 1448183_a_at | 2.33 | Hif1a | hypoxia inducible factor 1, alpha subunit |
| 1420858_at | 2.32 | Pkia | protein kinase inhibitor, alpha |
| 1415784_at | 2.32 | Vps35 | vacuolar protein sorting 35 |
| 1425576_at | 2.30 | Ahcyl1 | S-adenosylhomocysteine hydrolase-like 1 |
| 1452444_at | 2.30 | Napb | N-ethylmaleimide sensitive fusion protein attachment protein beta |
| 1431191_a_at | 2.27 | Syt1 | synaptotagmin I |
| 1429332_at | 2.27 | 4632427E13Rik | RIKEN cDNA 4632427E13 gene |
| 1450105_at | 2.27 | Adam10 | a disintegrin and metallopeptidase domain 10 |
| 1417502_at | 2.28 | Tspan7 | tetraspanin 7 |
| 1421851_at | 2.26 | Mtap1b | microtubule-associated protein 1B |
| 1448285_at | 2.26 | Rgs4 | regulator of G-protein signaling 4 |
| 1450037_at | 2.25 | Usp9x | ubiquitin specific peptidase 9, X chromosome |
| 1450379_at | 2.25 | Msn | moesin |
| 1450088_a_at | 2.27 | Mobp | myelin-associated oligodendrocytic basic protein |
| 1432646_a_at | 2.25 | 2900097C17Rik | RIKEN cDNA 2900097C17 gene |
| 1449682_s_at | 2.23 | Tubb2b | tubulin, beta 2a, pseudogene 2 |
| 1435635_at | 2.23 | Pcmtd1 | protein-L-isoaspartate (D-aspartate) O-methyltransferase domain containing 1 |
| 1421955_a_at | 2.23 | Nedd4 | neural precursor cell expressed, developmentally down-regulated 4 |
| 1443273_at | 2.22 | Epha3 | |
| 1449931_at | 2.21 | Cpeb4 | cytoplasmic polyadenylation element binding protein 4 |
| AFFX-r2-Bs-dap-5_at | 2.23 | | |
| 1450184_s_at | 2.19 | Tef | thyrotroph embryonic factor |
| 1431645_a_at | 2.21 | Gdi2 | guanosine diphosphate (GDP) dissociation inhibitor 2 |
| 1420975_at | 2.19 | Baz1b | bromodomain adjacent to zinc finger domain, 1B |
| 1421813_a_at | 2.22 | Psap | prosaposin |
| 1429786_a_at | 2.20 | Zwint | ZW10 interactor |
| 1416525_at | 2.19 | Spop | speckle-type POZ protein |
| 1425338_at | 2.16 | Plcb4 | phospholipase C, beta 4 |

| | | | |
|---|---|---|---|
| 1429685_at | 2.15 | Gabrb2 | gamma-aminobutyric acid (GABA-A) receptor, subunit beta 2 |
| 1453760_at | 2.15 | Mier1 | mesoderm induction early response 1 homolog (Xenopus laevis |
| 1438714_at | 2.14 | Zfp207 | |
| 1420506_a_at | 2.13 | Stxbp1 | syntaxin binding protein 1 |
| 1439041_at | 2.12 | Slc39a10 | solute carrier family 39 (zinc transporter), member 10 |
| 1415773_at | 2.15 | Ncl | nucleolin |
| AFFX-DapX-5_at | 2.13 | | |
| 1445061_at | 2.10 | Pcdh9 | |
| 1427058_at | 2.11 | Eif4a1 | eukaryotic translation initiation factor 4A1 |
| 1449264_at | 2.11 | Syt11 | synaptotagmin XI |
| 1431606_a_at | 2.10 | Angel2 | angel homolog 2 (Drosophila) |
| 1431096_at | 2.09 | Ints8 | integrator complex subunit 8 |
| 1439517_at | 2.09 | Mysm1 | |
| 1430981_s_at | 2.10 | Gpbp1 | GC-rich promoter binding protein 1 |
| 1416132_at | 2.10 | Efr3a | EFR3 homolog A (S. cerevisiae) |
| 1439300_at | 2.06 | Chic1 | cysteine-rich hydrophobic domain 1 |
| 1421070_at | 2.07 | D3Ertd300e | DNA segment, Chr 3, ERATO Doi 300, expressed |
| 1453540_at | 2.06 | 5430404G13Rik | RIKEN cDNA 5430404G13 gene |
| 1450407_a_at | 2.08 | Anp32a | acidic (leucine-rich) nuclear phosphoprotein 32 family, member A |
| 1441974_at | 2.05 | Camk4 | calcium/calmodulin-dependent protein kinase IV |
| 1458830_at | 2.06 | Fgf14 | |
| 1420951_a_at | 2.06 | Son | Son DNA binding protein |
| 1434106_at | 2.05 | Epm2aip1 | EPM2A (laforin) interacting protein 1 |
| 1450370_a_at | 2.03 | Kcnip4 | Kv channel interacting protein 4 |
| 1428402_at | 2.02 | Zcchc3 | zinc finger, CCHC domain containing 3 |
| 1448348_at | 2.03 | Caprin1 | cell cycle associated protein 1 |
| 1417462_at | 2.05 | Cap1 | CAP, adenylate cyclase-associated protein 1 (yeast) |
| 1435462_at | 2.01 | Plcxd2 | phosphatidylinositol-specific phospholipase C, X domain containing 2 |
| 1449645_s_at | 2.04 | Cct3 | chaperonin containing Tcp1, subunit 3 (gamma) |
| 1449228_at | 2.01 | Sh3gl2 | SH3-domain GRB2-like 2 |
| 1430996_at | 2.00 | Etnk1 | ethanolamine kinase 1 |
| 1424893_at | -2.01 | Ndel1 | nuclear distribution gene E-like homolog 1 (A. nidulans) |
| 1431216_s_at | 1.99 | Dnajc6 | DnaJ (Hsp40) homolog, subfamily C, member 6 |
| 1460295_s_at | 1.99 | Il6st | interleukin 6 signal transducer |
| 1446065_at | 1.98 | Kcnd2 | |
| 1426413_at | 2.00 | Neurod1 | neurogenic differentiation 1 |
| 1438556_a_at | 1.99 | Tmod3 | tropomodulin 3 |
| 1456610_at | 2.00 | Jmjd3 | jumonji domain containing 3 |
| 1447996_at | 1.97 | Ptchd1 | patched domain containing 1 |
| 1441373_at | -1.98 | Msi2 | |
| 1420344_x_at | -1.97 | Gzmd | granzyme D |
| 1419099_x_at | 1.96 | Stom | stomatin |
| 1430984_at | 1.96 | Azin1 | antizyme inhibitor 1 |
| 1458934_at | -1.96 | D5Ertd505e | DNA segment, Chr 5, ERATO Doi 505 , expressed |
| 1452526_a_at | 1.95 | Pax6 | paired box gene 6 |
| 1438009_at | 1.98 | Hist1h2ae | histone cluster 1, H2ae |
| 1450804_at | 1.94 | Kif5c | kinesin family member 5C |
| 1430533_a_at | 1.96 | Ctnnb1 | catenin (cadherin associated protein), beta 1 |
| 1452430_s_at | 1.94 | Sfrs1 | splicing factor, arginine/serine-rich 1 (ASF/SF2) |
| 1426229_s_at | 1.95 | Kras | v-Ki-ras2 Kirsten rat sarcoma viral oncogene homolog |
| 1432344_a_at | 1.94 | Aplp2 | amyloid beta (A4) precursor-like protein 2 |
| 1425461_at | 1.94 | Fbxw11 | F-box and WD-40 domain protein 11 |
| 1452308_a_at | 1.99 | Atp1a2 | ATPase, Na+/K+ transporting, alpha 2 polypeptide |
| 1421862_a_at | 1.94 | Vamp1 | vesicle-associated membrane protein 1 |
| 1458524_at | -1.93 | Fndc3a | fibronectin type III domain containing 3a |

| | | | |
|---|---|---|---|
| 1427470_s_at | 1.93 | Napb | N-ethylmaleimide sensitive fusion protein attachment protein beta |
| 1429113_at | 1.92 | Prrt2 | proline-rich transmembrane protein 2 |
| 1450038_s_at | 1.92 | Usp9x | ubiquitin specific peptidase 9, X chromosome |
| 1454043_a_at | 1.90 | Kcnab1 | potassium voltage-gated channel, shaker-related subfamily, beta member 1 |
| 1431031_at | 1.91 | Arid4b | AT rich interactive domain 4B (RBP1-like) |
| 1449054_a_at | 1.96 | Pcbp4 | poly(rC) binding protein 4 |
| 1449984_at | -1.91 | Cxcl2 | chemokine (C-X-C motif) ligand 2 |
| 1415893_at | 1.90 | Sgpl1 | sphingosine phosphate lyase 1 |
| 1451846_at | 1.91 | Nebl | nebulette |
| 1418452_at | 1.89 | Gng2 | guanine nucleotide binding protein (G protein), gamma 2 |
| 1460650_at | 1.89 | Atp6v0a1 | ATPase, H+ transporting, lysosomal V0 subunit A1 |
| AFFX-b-ActinMur/M12481_5_at | 1.93 | Actb | actin, beta |
| 1451447_at | 1.88 | Cuedc1 | CUE domain containing 1 |
| 1448458_at | 1.89 | Top2b | topoisomerase (DNA) II beta |
| 1428574_a_at | 1.89 | Chn2 | chimerin (chimaerin) 2 |
| 1418020_s_at | 1.88 | Cpd | carboxypeptidase D |
| 1416162_at | 1.86 | Rad21 | RAD21 homolog (S. pombe) |
| 1453307_a_at | 1.86 | Anapc5 | anaphase-promoting complex subunit 5 |
| 1450846_at | 1.85 | Bzw1 | basic leucine zipper and W2 domains 1 |
| 1421660_at | 1.84 | Scn9a | sodium channel, voltage-gated, type IX, alpha |
| 1421351_at | 1.84 | Gria4 | glutamate receptor, ionotropic, AMPA4 (alpha 4) |
| 1438130_at | -1.84 | Taf15 | TAF15 RNA polymerase II, TATA box binding protein (TBP)-associated factor |
| 1415997_at | 1.86 | Txnip | thioredoxin interacting protein |
| 1419098_at | 1.84 | Stom | stomatin |
| 1420867_at | 1.84 | Tmed2 | transmembrane emp24 domain trafficking protein 2 |
| 1448538_a_at | 1.85 | D4Wsu53e | DNA segment, Chr 4, Wayne State University 53, expressed |
| 1423325_at | 1.84 | Pnn | pinin |
| 1418768_at | 1.83 | Opa1 | optic atrophy 1 homolog (human) |
| 1453960_a_at | 1.85 | Capzb | capping protein (actin filament) muscle Z-line, beta |
| 1421013_at | 1.83 | Pitpnb | phosphatidylinositol transfer protein, beta |
| 1419728_at | -1.83 | Cxcl5 | chemokine (C-X-C motif) ligand 5 |
| 1425467_a_at | 1.83 | Plp1 | proteolipid protein (myelin) 1 |
| 1433804_at | 1.83 | Jak1 | Janus kinase 1 |
| 1443115_at | 1.81 | Tgfbr2 | |
| 1447513_at | 1.82 | Kcnd3 | potassium voltage-gated channel, Shal-related family, member 3 |
| 1444338_at | 1.82 | | |
| 1443728_at | 1.83 | | |
| 1422009_at | 1.81 | Atp1b2 | ATPase, Na+/K+ transporting, beta 2 polypeptide |
| 1455061_a_at | 1.84 | Acaa2 | acetyl-Coenzyme A acyltransferase 2 (mitochondrial 3-oxoacyl-Coenzyme A thiolase) |
| 1456637_at | 1.80 | Lrrtm2 | leucine rich repeat transmembrane neuronal 2 |
| 1427281_at | 1.81 | Scn2a1 | sodium channel, voltage-gated, type II, alpha 1 |
| 1421845_at | 1.81 | Golph3 | golgi phosphoprotein 3 |
| 1417029_a_at | 1.82 | Trim2 | tripartite motif-containing 2 |
| 1435145_at | 1.80 | Cadm2 | cell adhesion molecule 2 |
| 1450010_at | 1.80 | Hsd17b12 | hydroxysteroid (17-beta) dehydrogenase 12 |
| 1420920_a_at | 1.81 | Arf1 | ADP-ribosylation factor 1 |
| 1456088_at | 1.79 | Xiap | X-linked inhibitor of apoptosis |
| 1460241_a_at | 1.81 | St3gal5 | ST3 beta-galactoside alpha-2,3-sialyltransferase 5 |
| 1450392_at | 1.79 | Abca1 | ATP-binding cassette, sub-family A (ABC1), member 1 |
| 1421889_a_at | 1.80 | Aplp2 | amyloid beta (A4) precursor-like protein 2 |
| 1435239_at | 1.79 | Gria1 | glutamate receptor, ionotropic, AMPA1 (alpha 1) |
| 1450208_a_at | 1.78 | Elmo1 | engulfment and cell motility 1, ced-12 homolog (C. elegans) |
| 1444564_at | -1.80 | Apod | similar to apolipoprotein D |
| 1422966_a_at | 1.78 | Tfrc | transferrin receptor |
| 1453740_a_at | 1.79 | Ccnl2 | cyclin L2 |

| | | | |
|---|---|---|---|
| 1421281_at | 1.78 | Gabra1 | gamma-aminobutyric acid (GABA-A) receptor, subunit alpha 1 |
| 1455445_at | 1.78 | Cbln3 | cerebellin 3 precursor protein |
| 1421102_a_at | 1.77 | Vamp3 | vesicle-associated membrane protein 3 |
| 1450007_at | 1.76 | 1500003O03Rik | RIKEN cDNA 1500003O03 gene |
| 1433491_at | 1.77 | Epb4.1l2 | erythrocyte protein band 4.1-like 2 |
| 1421832_at | 1.76 | Twsg1 | twisted gastrulation homolog 1 (Drosophila) |
| 1416562_at | 1.75 | Gad1 | glutamic acid decarboxylase 1 |
| 1425861_x_at | 1.75 | Cacna2d1 | calcium channel, voltage-dependent, alpha2/delta subunit 1 |
| 1421141_a_at | 1.76 | Foxp1 | forkhead box P1 |
| 1449153_at | -1.75 | Mmp12 | matrix metallopeptidase 12 |
| 1428718_at | 1.76 | Scrn1 | secernin 1 |
| 1433492_at | 1.75 | Epb4.1l2 | erythrocyte protein band 4.1-like 2 |
| 1460279_a_at | 1.76 | Gtf2i | general transcription factor II I |
| 1421190_at | 1.74 | Gabrb3 | gamma-aminobutyric acid (GABA-A) receptor, subunit beta 3 |
| 1455986_at | 1.75 | Zdhhc17 | zinc finger, DHHC domain containing 17 |
| 1421328_at | 1.74 | Mtap2 | microtubule-associated protein 2 |
| 1434357_a_at | 1.75 | Kpnb1 | karyopherin (importin) beta 1 |
| 1441785_at | 1.74 | Gm879 | gene model 879, (NCBI) |
| 1425911_a_at | 1.77 | Fgfr1 | fibroblast growth factor receptor 1 |
| 1422135_at | 1.74 | Zfp146 | zinc finger protein 146 |
| 1438397_a_at | 1.75 | Rbm39 | RNA binding motif protein 39 |
| 1425622_at | 1.73 | Edil3 | EGF-like repeats and discoidin I-like domains 3 |
| 1426831_at | 1.74 | Ahcyl1 | S-adenosylhomocysteine hydrolase-like 1 |
| 1452688_at | 1.73 | Prpf39 | PRP39 pre-mRNA processing factor 39 homolog (yeast) |
| 1425929_a_at | 1.74 | Rnf14 | ring finger protein 14 |
| 1439151_at | 1.73 | Msrb3 | methionine sulfoxide reductase B3 |
| 1447473_at | 1.72 | | |
| 1422249_s_at | 1.73 | Zfa | zinc finger protein, autosomal |
| 1431042_at | 1.74 | Paqr8 | progestin and adipoQ receptor family member VIII |
| 1436858_at | 1.74 | Mbnl2 | muscleblind-like 2 |
| 1425660_at | 1.72 | Btbd3 | BTB (POZ) domain containing 3 |
| 1422051_a_at | 1.72 | Gabbr1 | gamma-aminobutyric acid (GABA-B) receptor, 1 |
| 1423598_at | 1.72 | Atp8a1 | ATPase, aminophospholipid transporter (APLT), class I, type 8A, member 1 |
| 1428909_at | -1.72 | A130040M12Rik | RIKEN cDNA A130040M12 gene |
| 1456127_at | 1.73 | Cnpy1 | canopy 1 homolog (zebrafish) |
| 1416190_a_at | 1.73 | Sec61a1 | Sec61 alpha 1 subunit (S. cerevisiae) |
| 1458719_at | -1.72 | | |
| 1421116_a_at | 1.73 | Rtn4 | reticulon 4 |
| AFFX-TransRecMur/X57349_3_at | 1.72 | Tfrc | transferrin receptor |
| 1436706_at | 1.72 | Tmem32 | transmembrane protein 32 |
| 1449262_s_at | 1.72 | Lin7c | lin-7 homolog C (C. elegans) |
| 1418309_at | 1.72 | Tnfrsf11b | tumor necrosis factor receptor superfamily, member 11b (osteoprotegerin) |
| 1453801_at | -1.71 | Them5 | thioesterase superfamily member 5 |
| 1446102_at | 1.70 | D9Ertd292e | DNA segment, Chr 9, ERATO Doi 292, expressed |
| 1418498_at | 1.71 | Fgf13 | fibroblast growth factor 13 |
| 1420901_a_at | 1.72 | Hk1 | hexokinase 1 |
| 1448364_at | 1.71 | Ccng2 | cyclin G2 |
| 1454174_a_at | 1.70 | C330007P06Rik | RIKEN cDNA C330007P06 gene |
| 1425487_at | 1.71 | Slu7 | SLU7 splicing factor homolog (S. cerevisiae) |
| 1458368_at | 1.70 | Myh4 | myosin, heavy polypeptide 4, skeletal muscle |
| 1426319_at | 1.69 | Pdgfd | platelet-derived growth factor, D polypeptide |
| 1416484_at | 1.70 | Ttc3 | tetratricopeptide repeat domain 3 |
| 1417356_at | 1.69 | Peg3 | paternally expressed 3 |
| 1446612_at | 1.69 | 9330118A15Rik | RIKEN cDNA 9330118A15 gene |
| 1431233_at | 1.69 | Cnnm4 | cyclin M4 |

| | | | |
|---|---|---|---|
| 1448732_at | 1.72 | Ctsb | cathepsin B |
| 1453163_at | 1.68 | Ppp1r12a | protein phosphatase 1, regulatory (inhibitor) subunit 12A |
| 1418126_at | -1.68 | Ccl5 | chemokine (C-C motif) ligand 5 |
| 1444454_at | 1.68 | Rb1 | |
| 1446682_at | 1.67 | | |
| 1430692_a_at | 1.68 | Sel1l | sel-1 suppressor of lin-12-like (C. elegans) |
| 1420924_at | 1.68 | Timp2 | tissue inhibitor of metalloproteinase 2 |
| 1452030_a_at | 1.68 | Hnrnpr | heterogeneous nuclear ribonucleoprotein R |
| 1422967_a_at | 1.67 | Tfrc | transferrin receptor |
| 1428820_at | 1.67 | Mapre1 | microtubule-associated protein, RP/EB family, member 1 |
| 1456398_at | 1.67 | Tug1 | taurine upregulated gene 1 |
| 1416732_at | 1.67 | Top2b | topoisomerase (DNA) II beta |
| 1415957_a_at | 1.69 | Rrp1 | ribosomal RNA processing 1 homolog (S. cerevisiae) |
| 1460717_at | 1.66 | Tspyl1 | testis-specific protein, Y-encoded-like 1 |
| 1441101_at | 1.67 | Hecw1 | HECT, C2 and WW domain containing E3 ubiquitin protein ligase 1 |
| 1422017_s_at | 1.66 | 4833439L19Rik | RIKEN cDNA 4833439L19 gene |
| 1420856_a_at | 1.66 | Lancl2 | LanC (bacterial lantibiotic synthetase component C)-like 2 |
| 1427510_at | 1.66 | Gnai1 | guanine nucleotide binding protein (G protein), alpha inhibiting 1 |
| 1418292_at | 1.69 | Asna1 | arsA arsenite transporter, ATP-binding, homolog 1 (bacterial) |
| 1450166_at | 1.65 | Ids | iduronate 2-sulfatase |
| 1419549_at | -1.67 | Arg1 | arginase, liver |
| 1439305_at | 1.67 | | |
| 1418536_at | -1.65 | | histocompatibility 2, Q region locus 7 |
| 1439423_x_at | -1.66 | U46068 | cDNA sequence U46068 |
| 1421924_at | 1.65 | Slc2a3 | solute carrier family 2 (facilitated glucose transporter), member 3 |
| 1428888_at | 1.65 | Tmem33 | transmembrane protein 33 |
| 1456827_at | 1.65 | Zfp87 | zinc finger protein 87 |
| 1440862_at | 1.64 | | |
| 1421227_at | -1.65 | Gzme | granzyme E |
| 1456386_at | 1.65 | Rbm39 | |
| 1427381_at | -1.65 | Irg1 | immunoresponsive gene 1 |
| 1452806_at | 1.66 | 1500016O10Rik | RIKEN cDNA 1500016O10 gene |
| 1453988_a_at | 1.64 | Ide | insulin degrading enzyme |
| 1425539_a_at | 1.65 | Rtn3 | reticulon 3 |
| 1416986_a_at | 1.64 | Sirpa | signal-regulatory protein alpha |
| 1440651_at | 1.63 | Dusp16 | |
| 1450108_at | 1.65 | Kif1a | kinesin family member 1A |
| 1425227_a_at | 1.63 | Atp6v0a1 | ATPase, H+ transporting, lysosomal V0 subunit A1 |
| 1452427_s_at | 1.64 | Ptplad1 | protein tyrosine phosphatase-like A domain containing 1 |
| 1422130_at | 1.63 | Nptx1 | neuronal pentraxin 1 |
| 1440228_at | 1.63 | Ranbp6 | RAN binding protein 6 |
| 1443437_at | -1.63 | EG633285 | |
| AFFX-r2-Bs-thr-5_s_at | 1.63 | | |
| 1418652_at | -1.63 | Cxcl9 | chemokine (C-X-C motif) ligand 9 |
| 1448793_a_at | -1.63 | Sdc4 | syndecan 4 |
| 1422565_s_at | 1.63 | Nfic | nuclear factor I/C |
| 1436530_at | -1.62 | OTTMUSG00000000971 | predicted gene, OTTMUSG00000000971 |
| 1450291_s_at | -1.62 | Ms4a4c | membrane-spanning 4-domains, subfamily A, member 4C |
| 1443536_at | 1.62 | Slc7a11 | solute carrier family 7 (cationic amino acid transporter, y+ system), member 11 |
| 1456656_at | 1.62 | Lin7a | lin-7 homolog A (C. elegans) |
| 1422911_at | 1.62 | Cbln3 | cerebellin 3 precursor protein |
| 1421448_at | 1.62 | Garnl1 | GTPase activating RANGAP domain-like 1 |
| 1422959_s_at | 1.61 | Zfp313 | ring finger protein 114 |
| 1426824_at | 1.62 | Psme4 | proteasome (prosome, macropain) activator subunit 4 |
| 1460310_a_at | 1.66 | Gh | growth hormone |

| | | | |
|---|---|---|---|
| 1418930_at | -1.61 | Cxcl10 | chemokine (C-X-C motif) ligand 10 |
| 1433015_at | 1.61 | 6330436F06Rik | RIKEN cDNA 6330436F06 gene |
| 1421606_a_at | 1.61 | Sult4a1 | sulfotransferase family 4A, member 1 |
