# Supplementary material for: Lack of nAChR Activity Depresses Cochlear Maturation and Up-Regulates GABA System Components: Temporal Profiling of Gene Expression in α9 Null Mice
Source: PLoS One. 2010 Feb 4;5(2):e9058. doi: 10.1371/journal.pone.0009058 (PMC2816210; doi:10.1371/journal.pone.0009058)
Supplement: Table S2 — Intersection of differentially expressed genes at P13 and P60. Intersection of differentially expressed genes at P13 and P60 along with their fold changes. Adjusted p-values<0.05, absolute fold changes >1.5. (0.06 MB PDF) [file pone.0009058.s003.pdf]

Table S2 - Intersection of differentially expressed genes at P13 and P60

Probe ID	Fold Change (log2) P13	Fold Change (log2) P60	Gene Symbol	Gene Description
1415784_at	3.433059	2.321088	Vps35	vacuolar protein sorting 35
1415801_at	4.101195	2.601822	Gja1	gap junction protein, alpha 1
1415823_at	5.406224	2.913104	Scd2	stearoyl-Coenzyme A desaturase 2
1415824_at	4.00198	2.915696	Scd2	stearoyl-Coenzyme A desaturase 2
1415893_at	2.817963	1.898136	Sgpl1	sphingosine phosphate lyase 1
1415957_a_at	3.141059	1.691971	Rrp1	ribosomal RNA processing 1 homolog (S. cerevisiae)
1415997_at	3.240269	1.855701	Txnip	thioredoxin interacting protein
1416190_a_at	3.895035	1.727618	Sec61a1	Sec61 alpha 1 subunit (S. cerevisiae)
1416484_at	3.221754	1.700582	Ttc3	tetratricopeptide repeat domain 3
1416525_at	3.607463	2.189889	Spop	speckle-type POZ protein
1416959_at	2.546208	2.412899	Nr1d2	nuclear receptor subfamily 1, group D, member 2
1416986_a_at	1.693842	1.637361	Sirpa	signal-regulatory protein alpha
1417029_a_at	2.513678	1.815466	Trim2	tripartite motif-containing 2
1417069_a_at	4.147902	2.753739	Gmfb	glia maturation factor, beta
1417502_at	3.387392	2.276252	Tspan7	tetraspanin 7
1418020_s_at	3.267313	1.879499	Cpd	carboxypeptidase D
1418292_at	2.48026	1.686748	Asna1	arsA arsenite transporter, ATP-binding, homolog 1 (bacterial)
1418452_at	2.195641	1.894159	Gng2	guanine nucleotide binding protein (G protein), gamma 2
1418768_at	1.530035	1.834519	Opa1	optic atrophy 1 homolog (human)
1419098_at	3.434734	1.835479	Stom	stomatin
1419099_x_at	3.303005	1.9617	Stom	stomatin
1420506_a_at	2.144152	2.129219	Stxbp1	syntaxin binding protein 1
1420610_at	3.021126	2.518742	Prkacb	protein kinase, cAMP dependent, catalytic, beta
1420816_at	2.268115	2.343326	Ywhag	tyrosine 3-monooxygenase/tryptophan 5-monooxygenase activation protein, gamma polypeptide
1420867_at	3.312996	1.83774	Tmed2	transmembrane emp24 domain trafficking protein 2
1420901_a_at	3.984849	1.724831	Hk1	hexokinase 1
1420924_at	3.578872	1.680721	Timp2	tissue inhibitor of metalloproteinase 2
1420948_s_at	2.931337	2.756593	Atrx	alpha thalassemia/mental retardation syndrome X-linked homolog (human)
1420951_a_at	3.11349	2.063684	Son	Son DNA binding protein
1420975_at	3.209455	2.189343	Baz1b	bromodomain adjacent to zinc finger domain, 1B
1421013_at	1.954613	1.82984	Pitpnb	phosphatidylinositol transfer protein, beta
1421070_at	2.59621	2.072055	D3Ertd300e	DNA segment, Chr 3, ERATO Doi 300, expressed
1421102_a_at	2.69399	1.767351	Vamp3	vesicle-associated membrane protein 3
1421606_a_at	2.414538	1.606752	Sult4a1	sulfotransferase family 4A, member 1
1421832_at	2.87429	1.757248	Twsg1	twisted gastrulation homolog 1 (Drosophila)
1421851_at	3.883313	2.260612	Mtap1b	microtubule-associated protein 1B
1421862_a_at	2.439096	1.938734	Vamp1	vesicle-associated membrane protein 1
1421866_at	2.829756	2.509237	Nr3c1	nuclear receptor subfamily 3, group C, member 1
1421955_a_at	3.319549	2.228188	Nedd4	neural precursor cell expressed, developmentally down-regulated 4
1421990_at	1.529026	3.08988	Syt1	synaptotagmin I
1422009_at	3.29081	1.810695	Atp1b2	ATPase, Na+/K+ transporting, beta 2 polypeptide
1422017_s_at	2.250677	1.658762	4833439L19Rik	RIKEN cDNA 4833439L19 gene
1422249_s_at	2.461006	1.726582	Zfa	zinc finger protein, autosomal
1422959_s_at	2.40897	1.610084	Zfp313	ring finger protein 114
1422966_a_at	2.005324	1.783075	Tfrc	transferrin receptor
1422967_a_at	3.44129	1.669243	Tfrc	transferrin receptor
1423325_at	2.922166	1.837624	Pnn	pinin
1423598_at	1.839129	1.722407	Atp8a1	ATPase, aminophospholipid transporter (APLT), class I, type 8A, member 1
1425338_at	1.931654	2.156073	Plcb4	phospholipase C, beta 4
1425461_at	2.579796	1.936184	Fbxw11	F-box and WD-40 domain protein 11
1425467_a_at	2.762797	1.827791	Plp1	proteolipid protein (myelin) 1
1425487_at	2.418868	1.7061	Slu7	SLU7 splicing factor homolog (S. cerevisiae)
1425539_a_at	2.339107	1.652486	Rtn3	reticulon 3

1425576_at	2.976323	2.300633	Ahcyl1	S-adenosylhomocysteine hydrolase-like 1
1425911_a_at	4.294646	1.767592	Fgfr1	fibroblast growth factor receptor 1
1427058_at	2.40955	2.108888	Eif4a1	eukaryotic translation initiation factor 4A1
1427470_s_at	3.166934	1.930992	Napb	N-ethylmaleimide sensitive fusion protein attachment protein beta
1428820_at	2.640228	1.669406	Mapre1	microtubule-associated protein, RP/EB family, member 1
1428888_at	2.043293	1.648453	Tmem33	transmembrane protein 33
1430533_a_at	3.983495	1.9611	Ctnnb1	catenin (cadherin associated protein), beta 1
1430692_a_at	2.275132	1.680689	Sel1l	sel-1 suppressor of lin-12-like (C. elegans)
1430980_a_at	4.520959	2.95185	Eif4a1	eukaryotic translation initiation factor 4A1
1430981_s_at	3.514609	2.096159	Gpbp1	GC-rich promoter binding protein 1
1430984_at	2.257294	1.957925	Azin1	antizyme inhibitor 1
1430996_at	2.130542	2.000058	Etnk1	ethanolamine kinase 1
1431030_a_at	4.433675	2.725354	Rnf14	ring finger protein 14
1431216_s_at	1.579359	1.987764	Dnajc6	DnaJ (Hsp40) homolog, subfamily C, member 6
1431233_at	2.93336	1.688964	Cnnm4	cyclin M4
1431606_a_at	2.867901	2.099579	Angel2	angel homolog 2 (Drosophila)
1431686_a_at	3.917631	2.584548	Gmfb	glia maturation factor, beta
1432344_a_at	2.20097	1.937626	Aplp2	amyloid beta (A4) precursor-like protein 2
1433492_at	2.902238	1.74905	Epb4.1l2	erythrocyte protein band 4.1-like 2
1433515_s_at	3.442424	2.446707	Etnk1	ethanolamine kinase 1
1433804_at	3.063185	1.825171	Jak1	Janus kinase 1
1434106_at	3.012675	2.049006	Epm2aip1	EPM2A (laforin) interacting protein 1
1434357_a_at	3.246418	1.745137	Kpnb1	karyopherin (importin) beta 1
1435635_at	2.989369	2.232329	Pcmtd1	protein-L-isoaspartate (D-aspartate) O-methyltransferase domain containing 1
1436706_at	2.555032	1.720037	Tmem32	transmembrane protein 32
1436858_at	3.546539	1.737685	Mbnl2	muscleblind-like 2
1438556_a_at	2.45016	1.987141	Tmod3	tropomodulin 3
1438714_at	1.731712	2.136938	Zfp207	
1439151_at	2.388456	1.725848	Msrb3	methionine sulfoxide reductase B3
1439305_at	2.263026	1.672706		
1439517_at	2.667241	2.085226	Mysm1	
1440862_at	1.552168	1.641955		
1442019_at	3.92711	3.01256	Rcvrn	
1443728_at	2.699002	1.8324		
1444001_at	3.280561	2.437371	Strbp	
1447360_at	4.972183	4.418096	Tsc22d1	TSC22 domain family, member 1
1448183_a_at	2.857963	2.333977	Hif1a	hypoxia inducible factor 1, alpha subunit
1448285_at	2.492461	2.255611	Rgs4	regulator of G-protein signaling 4
1448348_at	2.966815	2.025491	Caprin1	cell cycle associated protein 1
1448458_at	3.188629	1.889407	Top2b	topoisomerase (DNA) II beta
1448538_a_at	4.253041	1.847343	D4Wsu53e	DNA segment, Chr 4, Wayne State University 53, expressed
1448541_at	3.872011	2.552021	Klc1	kinesin light chain 1
1449054_a_at	2.966922	1.959988	Pcbp4	poly(rC) binding protein 4
1449262_s_at	2.100273	1.715523	Lin7c	lin-7 homolog C (C. elegans)
1449264_at	2.94458	2.105196	Syt11	synaptotagmin XI
1449682_s_at	2.481223	2.226367	Tubb2b	tubulin, beta 2a, pseudogene 2
1449931_at	2.242467	2.205036	Cpeb4	cytoplasmic polyadenylation element binding protein 4
1450007_at	2.757845	1.761852	1500003O03Rik	RIKEN cDNA 1500003O03 gene
1450037_at	2.958919	2.248753	Usp9x	ubiquitin specific peptidase 9, X chromosome
1450038_s_at	2.505561	1.915591	Usp9x	ubiquitin specific peptidase 9, X chromosome
1450108_at	2.244475	1.654364	Kif1a	kinesin family member 1A
1450208_a_at	1.840287	1.781292	Elmo1	engulfment and cell motility 1, ced-12 homolog (C. elegans)
1450291_s_at	-1.812101	-1.620883	Ms4a4c	membrane-spanning 4-domains, subfamily A, member 4C
1450379_at	3.792238	2.252815	Msn	moesin
1450392_at	3.477232	1.793951	Abca1	ATP-binding cassette, sub-family A (ABC1), member 1
1450846_at	2.473852	1.85175	Bzw1	basic leucine zipper and W2 domains 1
1451285_at	4.123669	2.573944	Fus	fusion, derived from t(12;16) malignant liposarcoma (human)

1451846_at	3.178015	1.906572	Nebl	nebulette
1451961_a_at	5.844186	2.796962	Mbp	myelin basic protein
1452030_a_at	2.984259	1.683975	Hnrnpr	heterogeneous nuclear ribonucleoprotein R
1452308_a_at	3.267716	1.987681	Atp1a2	ATPase, Na+/K+ transporting, alpha 2 polypeptide
1452427_s_at	2.864206	1.636373	Ptplad1	protein tyrosine phosphatase-like A domain containing 1
1452444_at	2.968607	2.301508	Napb	N-ethylmaleimide sensitive fusion protein attachment protein beta
1452638_s_at	3.117866	2.711231	Dnm1l	dynamin 1-like
1452688_at	1.695354	1.726259	Prpf39	PRP39 pre-mRNA processing factor 39 homolog (yeast)
1452806_at	2.46267	1.663138	1500016O10Rik	RIKEN cDNA 1500016O10 gene
1453163_at	2.412696	1.680639	Ppp1r12a	protein phosphatase 1, regulatory (inhibitor) subunit 12A
1453307_a_at	3.823676	1.863647	Anapc5	anaphase-promoting complex subunit 5
1453740_a_at	4.213176	1.790344	Ccnl2	cyclin L2
1453760_at	2.617526	2.151796	Mier1	mesoderm induction early response 1 homolog (Xenopus laevis)
1453960_a_at	2.994733	1.849085	Capzb	capping protein (actin filament) muscle Z-line, beta
1453988_a_at	2.871974	1.644895	Ide	insulin degrading enzyme
1454174_a_at	1.818308	1.700105	C330007P06Rik	RIKEN cDNA C330007P06 gene
1455986_at	2.145313	1.745366	Zdhhc17	zinc finger, DHHC domain containing 17
1456080_a_at	4.937984	2.481565	Serinc3	serine incorporator 3
1456088_at	2.458152	1.792742	Xiap	X-linked inhibitor of apoptosis
1456386_at	3.010843	1.647867	Rbm39	
1456398_at	2.774336	1.665971	Tug1	taurine upregulated gene 1
1456610_at	2.887038	1.998251	Jmjd3	jumonji domain containing 3
1456827_at	2.290211	1.649953	Zfp87	zinc finger protein 87
1458676_at	3.844988	2.762408	Nktr	natural killer tumor recognition sequence
1460241_a_at	2.990871	1.810792	St3gal5	ST3 beta-galactoside alpha-2,3-sialyltransferase 5
1460279_a_at	3.9904	1.764153	Gtf2i	general transcription factor II I
1460295_s_at	2.958201	1.994436	Il6st	interleukin 6 signal transducer
1460650_at	1.986057	1.894885	Atp6v0a1	ATPase, H+ transporting, lysosomal V0 subunit A1
1460717_at	2.45665	1.663754	Tspyl1	testis-specific protein, Y-encoded-like 1
AFFX-b-ActinMur/M12481_5_at	3.435492	1.934098	Actb	actin, beta
AFFX-GapdhMur/M32599_5_at	5.34447	2.673308	Gapdh	glyceraldehyde-3-phosphate dehydrogenase
AFFX-TransRecMur/X57349_3_at	1.993207	1.717723	Tfrc	transferrin receptor
