# Supplementary material for: Lack of nAChR Activity Depresses Cochlear Maturation and Up-Regulates GABA System Components: Temporal Profiling of Gene Expression in α9 Null Mice
Source: PLoS One. 2010 Feb 4;5(2):e9058. doi: 10.1371/journal.pone.0009058 (PMC2816210; doi:10.1371/journal.pone.0009058)
Supplement: Table S3 — Functional annotations for biclusters obtained with ISA and details for bicluster9. Table S3A contains statistically over-represented Panther categories as identified by DAVID for all 33 biclusters. Table S3B includes the gene content of bicluster9, along with Affymetrix probe identifiers. (0.40 MB DOC) [file pone.0009058.s004.doc]

| **Bic #** | **Top Overrepresented Panther Categories (Biological Function)** | **adjusted *p*-value** | **Top Overrepresented Panther Categories (Molecular Function)** | **adjusted *p*-value** |
| --- | --- | --- | --- | --- |
| 1 | BP00285:Cell structure and motility | 3.60E-05 | MF00091:Cytoskeletal protein | 3.80E-05 |
| 2 | BP00199:Neurogenesis | 2.20E-02 | MF00213:Non-receptor serine/threonine protein kinase | 7.50E-05 |
| 3 | BP00285:Cell structure and motility | 1.80E-10 | MF00091:Cytoskeletal protein | 1.00E-05 |
| 4 | BP00019:Lipid, fatty acid and steroid metabolism | 8.00E-04 | MF00034:Voltage-gated potassium channel | 3.60E-02 |
| 5 | None | NA | None | NA |
| 6 | BP00071:Proteolysis | 7.00E-04 | MF00213:Non-receptor serine/threonine protein kinase | 5.20E-03 |
| 7 | BP00285:Cell structure and motility | 2.20E-04 | MF00042:Nucleic acid binding | 8.10E-07 |
| 8 | BP00071:Proteolysis | 1.20E-04 | MF00267:Membrane traffic protein | 1.90E-02 |
| 9 | BP00071:Proteolysis | 3.00E-04 | MF00042:Nucleic acid binding | 2.10E-06 |
| 10 | BP00071:Proteolysis | 5.80E-05 | MF00267:Membrane traffic protein | 4.10E-03 |
| 11 | BP00071:Proteolysis | 7.90E-06 | MF00213:Non-receptor serine/threonine protein kinase | 1.30E-05 |
| 12 | None |  | none |  |
| 13 | BP00071:Proteolysis | 2.20E-05 | MF00213:Non-receptor serine/threonine protein kinase | 2.00E-04 |
| 14 | BP00287:Cell motility | 4.2E-04 | MF00213:Non-receptor serine/threonine protein kinase | 1.90E-04 |
| 15 | None | NA | None | NA |
| 16 | BP00143:Cation transport | 6.20E-04 | MF00213:Non-receptor serine/threonine protein kinase | 2.30E-05 |
| 17 | None | NA | None | NA |
| 18 | None |  | MF00262:Non-motor actin binding protein | 4.40E-02 |
| 19-33 | None | NA | None | NA |

**Table S3A: Overrepresented Panther categories of neuronal-biclusters.** Top

overrepresented Panther categories are listed in the table below. DAVID (Database for Annotation, Visualization and Integrated Discovery) tool was used to functionally annotate biclusters. Adjusted p-values (BH) are reported for each category.

**Table S3B: Bicluster9 gene list.** Affymetrix probe IDs, gene symbols and gene descriptions for Bicluster9 is indicated below.

| **Probe ID** | **Gene Symbol** | **Gene Description** |
| --- | --- | --- |
| 1415804_at | Cx3cl1 | chemokine (C-X3-C motif) ligand 1 |
| 1415843_at | Gbl | G protein beta subunit-like |
| 1415988_at | Hdlbp | high density lipoprotein (HDL) binding protein |
| 1416204_at | Gpd1 | glycerol-3-phosphate dehydrogenase 1 (soluble) |
| 1416348_at | Men1 | multiple endocrine neoplasia 1 |
| 1416515_at | Fscn1 | fascin homolog 1, actin bundling protein (Strongylocentrotus purpuratus) |
| 1416516_at | Fscn1 | fascin homolog 1, actin bundling protein (Strongylocentrotus purpuratus) |
| 1416521_at | Sepw1 | selenoprotein W, muscle 1 |
| 1416798_a_at | Nme4 | non-metastatic cells 4, protein expressed in |
| 1416851_at | St13 | suppression of tumorigenicity 13 |
| 1417198_at | Wwc2 | WW, C2 and coiled-coil domain containing 2 |
| 1417229_at | Capn1 | calpain 1 |
| 1417230_at | Ralgps2 | Ral GEF with PH domain and SH3 binding motif 2 |
| 1417502_at | Tspan7 | tetraspanin 7 |
| 1417548_at | Sart3 | squamous cell carcinoma antigen recognized by T-cells 3 |
| 1417613_at | Ier5 | immediate early response 5 |
| 1417678_at | Mmp24 | matrix metallopeptidase 24 |
| 1417860_a_at | Spon2 | spondin 2, extracellular matrix protein |
| 1417882_at | Slc39a3 | solute carrier family 39 (zinc transporter), member 3 |
| 1418795_at | Cds2 | CDP-diacylglycerol synthase (phosphatidate cytidylyltransferase) 2 |
| 1418938_at | Dio2 | deiodinase, iodothyronine, type II |
| 1419022_a_at | Eno1 | enolase 1, alpha non-neuron |
| 1419092_a_at | Slk | STE20-like kinase (yeast) |
| 1419099_x_at | Stom | stomatin |
| 1419372_at | Gosr2 | golgi SNAP receptor complex member 2 |
| 1419667_at | Sgcb | sarcoglycan, beta (dystrophin-associated glycoprotein) |
| 1419679_at | Lats2 | large tumor suppressor 2 |
| 1419682_a_at | Trp53rk | transformation related protein 53 regulating kinase |
| 1419864_x_at | Tnpo1 | transportin 1 |
| 1420373_at | Foxj2 | forkhead box J2 |
| 1420506_a_at | Stxbp1 | syntaxin binding protein 1 |
| 1420669_at | Arnt2 | aryl hydrocarbon receptor nuclear translocator 2 |
| 1420670_at | Arnt2 | aryl hydrocarbon receptor nuclear translocator 2 |
| 1420744_at | Chrnb2 | cholinergic receptor, nicotinic, beta polypeptide 2 (neuronal) |
| 1420749_a_at | Pou6f1 | POU domain, class 6, transcription factor 1 |
| 1420833_at | Vamp2 | vesicle-associated membrane protein 2 |
| 1420881_at | Acd | adrenocortical dysplasia |
| 1420893_a_at | Tgfbr1 | transforming growth factor, beta receptor I |
| 1420923_at | Usp9x | ubiquitin specific peptidase 9, X chromosome |
| 1420927_at | St6gal1 | beta galactoside alpha 2,6 sialyltransferase 1 |
| 1420954_a_at | Add1 | adducin 1 (alpha) |
| 1420968_at | Btbd14b | BTB (POZ) domain containing 14B |
| 1421032_a_at | Dnajb12 | DnaJ (Hsp40) homolog, subfamily B, member 12 |
| 1421053_at | Kif1a | kinesin family member 1A |
| 1421060_at | Mllt1 | myeloid/lymphoid or mixed-lineage leukemia  (trithorax homolog, Drosophila); translocated to, 1 |
| 1421146_at | Rapgef1 | Rap guanine nucleotide exchange factor (GEF) 1 |
| 1421181_at | Nptxr | neuronal pentraxin receptor |
| 1421200_at | Dlg2 | discs, large homolog 2 (Drosophila) |
| 1421216_a_at | Ids | iduronate 2-sulfatase |
| 1421232_at | Plxna1 | plexin A1 |
| 1421239_at | Il6st | interleukin 6 signal transducer |
| 1421298_a_at | Hipk1 | homeodomain interacting protein kinase 1 |
| 1421339_at | Extl3 | exostoses (multiple)-like 3 |
| 1421368_at | Scrt1 | scratch homolog 1, zinc finger protein (Drosophila) |
| 1421376_at | Traf6 | Tnf receptor-associated factor 6 |
| 1421413_a_at | Pdlim5 | PDZ and LIM domain 5 |
| 1421480_a_at | Adarb1 | adenosine deaminase, RNA-specific, B1 |
| 1421518_at | Kcns1 | K+ voltage-gated channel, subfamily S, 1 |
| 1421673_s_at | Stx1b | syntaxin 1B |
| 1421789_s_at | Arf3 | ADP-ribosylation factor 3 |
| 1421900_at | Eif2ak1 | eukaryotic translation initiation factor 2 alpha kinase 1 |
| 1421949_a_at | Ccdc123 | coiled-coil domain containing 123 |
| 1421994_a_at | Hs1bp3 | HCLS1 binding protein 3 |
| 1422044_at | Ndst1 | N-deacetylase/N-sulfotransferase (heparan glucosaminyl) 1 |
| 1422107_at | 2410066E13Rik | RIKEN cDNA 2410066E13 gene |
| 1422119_at | Rab5b | RAB5B, member RAS oncogene family |
| 1422132_at | Mthfr | 5,10-methylenetetrahydrofolate reductase |
| 1422136_at | Uhmk1 | U2AF homology motif (UHM) kinase 1 |
| 1422223_at | Grin2b | glutamate receptor, ionotropic, NMDA2B (epsilon 2) |
| 1422281_at | Sstr4 | somatostatin receptor 4 |
| 1422314_at | Clcn6 | chloride channel 6 |
| 1422331_at | Pou3f3 | POU domain, class 3, transcription factor 3 |
| 1422967_a_at | Tfrc | transferrin receptor |
| 1422968_at | Ihpk1 | inositol hexaphosphate kinase 1 |
| 1422984_at | Clip2 | CAP-GLY domain containing linker protein 2 |
| 1423004_at | Vipr1 | vasoactive intestinal peptide receptor 1 |
| 1423075_at | Lman2 | lectin, mannose-binding 2 |
| 1423402_at | Creb1 | cAMP responsive element binding protein 1 |
| 1423528_at | Bcas3 | breast carcinoma amplified sequence 3 |
| 1423661_s_at | Ctdsp2 | CTD (carboxy-terminal domain, RNA polymerase II, polypeptide A)  small phosphatase 2 |
| 1424178_at | Tmem38a | transmembrane protein 38A |
| 1424486_a_at | Txnrd1 | thioredoxin reductase 1 |
| 1424538_at | Ubl4 | ubiquitin-like 4 |
| 1424787_a_at | Nrf1 | nuclear respiratory factor 1 |
| 1425097_a_at | Zfp106 | zinc finger protein 106 |
| 1425154_a_at | Csf1 | colony stimulating factor 1 (macrophage) |
| 1425170_a_at | Adam15 | a disintegrin and metallopeptidase domain 15 (metargidin) |
| 1425204_s_at | Ddx19b | DEAD (Asp-Glu-Ala-Asp) box polypeptide 19b |
| 1425227_a_at | Atp6v0a1 | ATPase, H+ transporting, lysosomal V0 subunit A1 |
| 1425337_at | Slc12a5 | solute carrier family 12, member 5 |
| 1425426_a_at | Mef2a | myocyte enhancer factor 2A |
| 1425535_at | Repin1 | replication initiator 1 |
| 1425587_a_at | Ptprj | protein tyrosine phosphatase, receptor type, J |
| 1425693_at | Braf | Braf transforming gene |
| 1425741_at | Srgap3 | SLIT-ROBO Rho GTPase activating protein 3 |
| 1425817_a_at | Slc8a1 | solute carrier family 8 (sodium/calcium exchanger), member 1 |
| 1425855_a_at | Crk | v-crk sarcoma virus CT10 oncogene homolog (avian) |
| 1425865_a_at | Lig3 | ligase III, DNA, ATP-dependent |
| 1426016_a_at | Tro | trophinin |
| 1426055_a_at | Pigq | phosphatidylinositol glycan anchor biosynthesis, class Q |
| 1426115_a_at | Kcnj9 | potassium inwardly-rectifying channel, subfamily J, member 9 |
| 1426125_a_at | Casp9 | caspase 9 |
| 1426179_a_at | Twsg1 | twisted gastrulation homolog 1 (Drosophila) |
| 1426191_a_at | Bcl2l1 | BCL2-like 1 |
| 1426335_at | Kcnq2 | potassium voltage-gated channel, subfamily Q, member 2 |
| 1426379_at | Eif4b | eukaryotic translation initiation factor 4B |
| 1426458_at | Slmap | sarcolemma associated protein |
| 1426538_a_at | Trp53 | transformation related protein 53 |
| 1426699_at | AU040320 | expressed sequence AU040320 |
| 1426795_at | Ptprs | protein tyrosine phosphatase, receptor type, S |
| 1426916_at | Scrn3 | secernin 3 |
| 1426975_at | Os9 | amplified in osteosarcoma |
| 1427077_a_at | Ap2b1 | adaptor-related protein complex 2, beta 1 subunit |
| 1427117_at | Mtmr3 | myotubularin related protein 3 |
| 1427652_x_at | Synj2 | synaptojanin 2 |
| 1427732_s_at | Abcg4 | ATP-binding cassette, sub-family G (WHITE), member 4 |
| 1427871_at | Ptafr | platelet-activating factor receptor |
| 1427967_at | Cdk5rap2 | CDK5 regulatory subunit associated protein 2 |
| 1428054_at | Slc8a2 | solute carrier family 8 (sodium/calcium exchanger), member 2 |
| 1428123_at | 2610528K11Rik | RIKEN cDNA 2610528K11 gene |
| 1428155_at | Commd9 | COMM domain containing 9 |
| 1428690_at | Tysnd1 | trypsin domain containing 1 |
| 1428850_x_at | Cd99 | CD99 antigen |
| 1428993_at | 1110017I16Rik | RIKEN cDNA 1110017I16 gene |
| 1429112_at | Tln2 | talin 2 |
| 1429362_a_at | Sf3b2 | splicing factor 3b, subunit 2 |
| 1429517_at | Zfyve20 | zinc finger, FYVE domain containing 20 |
| 1429566_a_at | Hipk2 | homeodomain interacting protein kinase 2 |
| 1429607_at | Trak2 | trafficking protein, kinesin binding 2 |
| 1429820_at | Sgsm1 | small G protein signaling modulator 1 |
| 1430021_a_at | Sae1 | SUMO1 activating enzyme subunit 1 |
| 1430024_at | Yipf6 | Yip1 domain family, member 6 |
| 1430144_at | 5830411G16Rik | RIKEN cDNA 5830411G16 gene |
| 1430275_a_at | Aqr | aquarius |
| 1430453_a_at | Bcl2l2 | BCL2-like 2 |
| 1430514_a_at | Cd99 | CD99 antigen |
| 1430564_at | Mobkl1a | MOB1, Mps One Binder kinase activator-like 1A (yeast) |
| 1430615_at | Ttll7 | tubulin tyrosine ligase-like family, member 7 |
| 1430631_at | Ppm1f | protein phosphatase 1F (PP2C domain containing) |
| 1430632_at | Asb1 | ankyrin repeat and SOCS box-containing 1 |
| 1430685_at | 6330503C03Rik | RIKEN cDNA 6330503C03 gene |
| 1430784_a_at | 4932417H02Rik | RIKEN cDNA 4932417H02 gene |
| 1430829_s_at | Fto | fat mass and obesity associated |
| 1430837_a_at | Mbd1 | methyl-CpG binding domain protein 1 |
| 1430971_a_at | Aqr | aquarius |
| 1430976_a_at | Mrpl9 | mitochondrial ribosomal protein L9 |
| 1430987_s_at | Wbp11 | WW domain binding protein 11 |
| 1431021_at | Cyb561d1 | cytochrome b-561 domain containing 1 |
| 1431092_at | Ppp1r12c | protein phosphatase 1, regulatory (inhibitor) subunit 12C |
| 1431097_at | Garnl1 | GTPase activating RANGAP domain-like 1 |
| 1431116_at | C030003D03Rik | RIKEN cDNA C030003D03 gene |
| 1431213_a_at | LOC67527 | murine leukemia retrovirus |
| 1431385_a_at | Mbtps1 | membrane-bound transcription factor peptidase, site 1 |
| 1431400_a_at | Gas7 | growth arrest specific 7 |
| 1431646_a_at | Stx6 | syntaxin 6 |
| 1431663_a_at | Cntfr | ciliary neurotrophic factor receptor |
| 1431675_a_at | Gtf2i | general transcription factor II I |
| 1431676_x_at | Gtf2i | general transcription factor II I |
| 1431828_a_at | Synj2 | synaptojanin 2 |
| 1431932_s_at | Trim44 | tripartite motif-containing 44 |
| 1432196_a_at | Dscaml1 | Down syndrome cell adhesion molecule-like 1 |
| 1432638_at | 2810002N01Rik | RIKEN cDNA 2810002N01 gene |
| 1433541_a_at | Ubap2l | ubiquitin associated protein 2-like |
| 1434466_at | Atcay | ataxia, cerebellar, Cayman type homolog (human) |
| 1434467_at | Atcay | ataxia, cerebellar, Cayman type homolog (human) |
| 1434668_at | Nucks1 | nuclear casein kinase and cyclin-dependent kinase substrate 1 |
| 1434885_at | Spty2d1 | SPT2, Suppressor of Ty, domain containing 1 (S. cerevisiae) |
| 1434902_at | Rnf157 | ring finger protein 157 |
| 1434938_at | Rbm9 | RNA binding motif protein 9 |
| 1435918_at | BC055107 | cDNA sequence BC055107 |
| 1435942_at | Kcnq2 | potassium voltage-gated channel, subfamily Q, member 2 |
| 1435944_s_at | Igh | immunoglobulin heavy chain complex |
| 1436083_at | Lrp3 | low density lipoprotein receptor-related protein 3 |
| 1436306_at | Saps1 | SAPS domain family, member 1 |
| 1436952_at | Klf9 | Kruppel-like factor 9 |
| 1437063_at | Fem1a | feminization 1 homolog a (C. elegans) |
| 1437452_x_at | Vdac1 | voltage-dependent anion channel 1 |
| 1437536_at | Fkrp | fukutin related protein |
| 1437648_at | Pcyt1b | phosphate cytidylyltransferase 1, choline, beta isoform |
| 1437653_at | Irgq | immunity-related GTPase family, Q |
| 1437768_at | Ankib1 | ankyrin repeat and IBR domain containing 1 |
| 1438043_at |  |  |
| 1438085_at | Heatr5b | HEAT repeat containing 5B |
| 1438138_a_at | Pex6 | peroxisomal biogenesis factor 6 |
| 1438253_at | Ssh1 | slingshot homolog 1 (Drosophila) |
| 1438425_at | Gtf3c1 | general transcription factor III C 1 |
| 1438440_at | Rnasen | ribonuclease III, nuclear |
| 1438686_at | Eif4g1 | eukaryotic translation initiation factor 4, gamma 1 |
| 1438860_a_at | Slc44a2 | solute carrier family 44, member 2 |
| 1439037_at | Ddx17 | DEAD (Asp-Glu-Ala-Asp) box polypeptide 17 |
| 1439076_at | Dhx29 | DEAH (Asp-Glu-Ala-His) box polypeptide 29 |
| 1439171_at | C530008M17Rik | RIKEN cDNA C530008M17 gene |
| 1439358_a_at | Nrxn1 | neurexin I |
| 1439359_x_at | Nrxn1 | neurexin I |
| 1439776_at | Ogfod1 | 2-oxoglutarate and iron-dependent oxygenase domain containing 1 |
| 1439910_a_at | Tradd | TNFRSF1A-associated via death domain |
| 1440031_at | Dact3 | dapper homolog 3, antagonist of beta-catenin (xenopus) |
| 1440288_at | Ptchd2 | patched domain containing 2 |
| 1440318_at | Wdr35 | WD repeat domain 35 |
| 1440437_at | Herc1 | hect (homologous to the E6-AP (UBE3A) carboxyl terminus)  domain and RCC1 (CHC1)-like domain (RLD) 1 |
| 1440480_at | AB182283 | cDNA sequence AB182283 |
| 1440844_at | Tob1 | transducer of ErbB-2.1 |
| 1441309_at | Adamts10 | a disintegrin-like and metallopeptidase (reprolysin type)  with thrombospondin type 1 motif, 10 |
| 1441928_x_at | Ell | elongation factor RNA polymerase II |
| 1442043_at | Lhfpl4 | lipoma HMGIC fusion partner-like protein 4 |
| 1442142_at | 2700050L05Rik | RIKEN cDNA 2700050L05 gene |
| 1442481_at | 675578 | predicted gene, 675578 |
| 1443597_at | Med14 | mediator complex subunit 14 |
| 1443701_at | Fam171a2 | family with sequence similarity 171, member A2 |
| 1443781_at |  |  |
| 1443916_at | 2900026A02Rik | RIKEN cDNA 2900026A02 gene |
| 1443980_at | Agrn | agrin |
| 1443991_at | Dock1 | dedicator of cytokinesis 1 |
| 1444055_at |  |  |
| 1444141_at | Snx13 | sorting nexin 13 |
| 1444298_at | A130090K04Rik | RIKEN cDNA A130090K04 gene |
| 1444357_at | Akap6 | A kinase (PRKA) anchor protein 6 |
| 1444511_at | Slc41a1 | solute carrier family 41, member 1 |
| 1444863_at |  |  |
| 1445189_at | Gpatch2 | G patch domain containing 2 |
| 1445241_at | Rab11fip4 | RAB11 family interacting protein 4 (class II) |
| 1446652_at | Reps2 | RALBP1 associated Eps domain containing protein 2 |
| 1446729_at | Disp2 | dispatched homolog 2 (Drosophila) |
| 1447207_at | Zyg11b | zyg-ll homolog B (C. elegans) |
| 1447369_at | 1190005F20Rik | RIKEN cDNA 1190005F20 gene |
| 1447538_at |  |  |
| 1447569_at | 1700081L11Rik | RIKEN cDNA 1700081L11 gene |
| 1447945_at | Maf | avian musculoaponeurotic fibrosarcoma (v-maf) AS42 oncogene homolog |
| 1448207_at | Lasp1 | LIM and SH3 protein 1 |
| 1448216_at | Syngr3 | synaptogyrin 3 |
| 1449129_a_at | Kcnip3 | Kv channel interacting protein 3, calsenilin |
| 1449552_at | Zfr | zinc finger RNA binding protein |
| 1450022_at | Gtpbp1 | GTP binding protein 1 |
| 1450041_a_at | Tub | tubby candidate gene |
| 1450100_a_at | Tcerg1 | transcription elongation regulator 1 (CA150) |
| 1450161_at | Ikbkg | inhibitor of kappaB kinase gamma |
| 1450229_at | Med14 | mediator complex subunit 14 |
| 1450326_at | Shc3 | src homology 2 domain-containing transforming protein C3 |
| 1450382_at | Nf2 | neurofibromatosis 2 |
| 1450402_at | Med1 | mediator complex subunit 1 |
| 1450500_at | Uhmk1 | U2AF homology motif (UHM) kinase 1 |
| 1450515_at | Kcnj11 | potassium inwardly rectifying channel, subfamily J, member 11 |
| 1450528_at | B3galt5 | UDP-Gal:betaGlcNAc beta 1,3-galactosyltransferase, polypeptide 5 |
| 1450556_at | Spnb1 | spectrin beta 1 |
| 1450651_at | Myo10 | myosin X |
| 1450747_at | Keap1 | kelch-like ECH-associated protein 1 |
| 1450799_at | Adcyap1r1 | adenylate cyclase activating polypeptide 1 receptor 1 |
| 1451250_at | Gprin1 | G protein-regulated inducer of neurite outgrowth 1 |
| 1451680_at | Srxn1 | sulfiredoxin 1 homolog (S. cerevisiae) |
| 1451795_at | Tom1l2 | target of myb1-like 2 (chicken) |
| 1451849_a_at | Lmnb2 | lamin B2 |
| 1451981_at | Gtrgeo22 | gene trap ROSA b-geo 22 |
| 1451992_at | Adrbk1 | adrenergic receptor kinase, beta 1 |
| 1452057_at | Actr1b | ARP1 actin-related protein 1 homolog B, centractin beta (yeast) |
| 1452174_at | Srebf2 | sterol regulatory element binding factor 2 |
| 1452185_at | Ipo8 | importin 8 |
| 1452413_at | C230081A13Rik | RIKEN cDNA C230081A13 gene |
| 1452419_at | Heatr1 | HEAT repeat containing 1 |
| 1452467_at | Mmab | methylmalonic aciduria (cobalamin deficiency) type B homolog (human) |
| 1452538_at | Igh-6 | immunoglobulin heavy chain 6 (heavy chain of IgM) |
| 1452684_at | Akt1s1 | AKT1 substrate 1 (proline-rich) |
| 1452747_at | Atp13a2 | ATPase type 13A2 |
| 1453114_at | Nol9 | nucleolar protein 9 |
| 1453242_x_at | 2810047C21Rik | RIKEN cDNA 2810047C21 gene |
| 1453556_x_at | Cd99 | CD99 antigen |
| 1453607_at | Mfap3l | microfibrillar-associated protein 3-like |
| 1453730_at | Samd8 | sterile alpha motif domain containing 8 |
| 1453751_at | Dhx38 | DEAH (Asp-Glu-Ala-His) box polypeptide 38 |
| 1453837_at | 6330500D04Rik | RIKEN cDNA 6330500D04 gene |
| 1453946_a_at | Sdccag8 | serologically defined colon cancer antigen 8 |
| 1453995_a_at | Htf9c | HpaII tiny fragments locus 9c |
| 1454106_a_at | Cxxc1 | CXXC finger 1 (PHD domain) |
| 1454184_a_at | Ikbkb | inhibitor of kappaB kinase beta |
| 1454250_at | Exoc6b | exocyst complex component 6B |
| 1455639_at | Slc25a39 | solute carrier family 25, member 39 |
| 1455691_at | Cyp21a1 | cytochrome P450, family 21, subfamily a, polypeptide 1 |
| 1455764_at | Lrpap1 | low density lipoprotein receptor-related protein associated protein 1 |
| 1455873_a_at | Vps18 | vacuolar protein sorting 18 (yeast) |
| 1456024_at | Gtf3c1 | general transcription factor III C 1 |
| 1456255_at | AI314180 | expressed sequence AI314180 |
| 1456304_at | Gm996 | gene model 996, (NCBI) |
| 1456486_at | Zfp574 | zinc finger protein 574 |
| 1456490_at | Cdk2ap2 | CDK2-associated protein 2 |
| 1456595_x_at | Gh | growth hormone |
| 1456608_at | Sac3d1 | SAC3 domain containing 1 |
| 1456675_at | Mprip | myosin phosphatase Rho interacting protein |
| 1456676_a_at | Pfkfb3 | 6-phosphofructo-2-kinase/fructose-2,6-biphosphatase 3 |
| 1457351_at | Taf2 | TAF2 RNA polymerase II, TATA box binding protein (TBP)-associated factor |
| 1458410_at | Garnl1 | GTPase activating RANGAP domain-like 1 |
| 1458411_at | Kndc1 | kinase non-catalytic C-lobe domain (KIND) containing 1 |
| 1458443_at | Crtc1 | CREB regulated transcription coactivator 1 |
| 1458636_at | Rnf219 | ring finger protein 219 |
| 1459317_at | Ank2 | ankyrin 2, brain |
| 1459385_at | Camsap1l1 | calmodulin regulated spectrin-associated protein 1-like 1 |
| 1459430_at | Gpr158 | G protein-coupled receptor 158 |
| 1459639_at | Brsk2 | BR serine/threonine kinase 2 |
| 1459766_x_at | Sf1 | splicing factor 1 |
| 1459769_at | BC048609 | cDNA sequence BC048609 |
| 1459832_s_at | Ap1m1 | adaptor-related protein complex AP-1, mu subunit 1 |
| 1460100_at |  |  |
| 1460269_at | Pnmt | phenylethanolamine-N-methyltransferase |
| 1460314_s_at | Hist2h3c1 | histone cluster 2, H3c1 |
| 1460353_at | Tmem48 | transmembrane protein 48 |
| AFFX-PyruCarbMur/L09192_5_at | Pcx | pyruvate carboxylase |
| AFFX-PyruCarbMur/L09192_MA_at | Pcx | pyruvate carboxylase |
| AFFX-TransRecMur/X57349_5_at | Tfrc | transferrin receptor |
