# Supplementary material for: Lack of nAChR Activity Depresses Cochlear Maturation and Up-Regulates GABA System Components: Temporal Profiling of Gene Expression in α9 Null Mice
Source: PLoS One. 2010 Feb 4;5(2):e9058. doi: 10.1371/journal.pone.0009058 (PMC2816210; doi:10.1371/journal.pone.0009058)
Supplement: Table S4 — Leading edge subsets for Bicluster9 for all ages. Leading edge subset of GSEA results for Bicluster9 for P3, P7, P13 and P60. Both Affymetrix Probe IDs and their corresponding gene symbols are given. The rank order of genes in each age indicates their contribution to the running sum score (with genes listed at the top contributing highly). (0.05 MB PDF) [file pone.0009058.s005.pdf]

Table S4- Leading edge subsets for Bicluster9 for P3, P7, P13, P60

Leading Edge Subsets							
**P60**	**P60 Gene Symbol**	**P13**	**P13 Gene Symbol**	**P7**	**P7 Gene Symbols**	**P3**	**P3 Gene Symbol**
1422044_at	NDST1	AFFX-TransRecMur/X57349_5_at	TFRC	1422044_at	NDST1	1438686_at	EIF4G1
1430615_at	4921517B04RIK	1430829_s_at	FTO	1431213_a_at	1300007C21RIK	1438425_at	GTF3C1
1421200_at	DLGH2	1419099_x_at	STOM	1431385_a_at	MBTPS1	1430631_at	PPM1F
1422967_a_at	TFRC	1454106_a_at	CXXC1	1421060_at	MLLT1	1431385_a_at	MBTPS1
1420881_at	NSD1	1426975_at	4632413K17RIK	1431675_a_at	GTF2I	1444511_at	SLC41A1
1447207_at	2810482G21RIK	1422967_a_at	TFRC	1456675_at	AA536749	1431213_a_at	1300007C21RIK
1419099_x_at	STOM	1444141_at	SNX13	1452419_at	HEATR1	1421060_at	MLLT1
1454106_a_at	CXXC1	1420893_a_at	TGFBR1	1430144_at	5830411G16RIK	1430971_a_at	AQR
1456255_at	AI314180	1456255_at	AI314180	1430971_a_at	AQR	1426795_at	PTPRS
AFFX-TransRecMur/X57349_5_at	TFRC	1427967_at	SRGAP2	1421339_at	EXTL3	1447207_at	2810482G21RIK
1420923_at	USP9X	1417229_at	CAPN1	1448207_at	LASP1	1417548_at	SART3
1431385_a_at	MBTPS1	1429362_a_at	SF3B2	1420881_at	NSD1	1448207_at	LASP1
1431675_a_at	GTF2I	1420506_a_at	STXBP1	1422331_at	POU3F3	1416516_at	FSCN1
1420893_a_at	TGFBR1	1415988_at	HDLBP	1438686_at	EIF4G1	1450651_at	MYO10
1420506_a_at	STXBP1	1431213_a_at	1300007C21RIK	1456024_at	GTF3C1	1447569_at	1700081L11RIK
1425337_at	SLC12A5	1442142_at	2700050L05RIK	1425337_at	SLC12A5	1430514_a_at	CD99
1426179_a_at	TWSG1	1417502_at	TSPAN7	1420923_at	USP9X	1421789_s_at	ARF3
1421789_s_at	ARF3	1428850_x_at	CD99	1451795_at	TOM1L2	1421232_at	PLXNA1
1456675_at	AA536749	1437768_at	ANKIB1	1420749_a_at	POU6F1	1453556_x_at	CD99
1425426_a_at	MEF2A	1420954_a_at	ADD1	1431097_at	GARNL1	1415843_at	GBL
1451795_at	TOM1L2	1453995_a_at	HTF9C	1430784_a_at	4932417H02RIK	1459769_at	ZFPL1
1427967_at	SRGAP2	1426179_a_at	TWSG1	1453242_x_at	2810047C21RIK	1430987_s_at	WBP11
1430971_a_at	AQR	1430514_a_at	CD99	1425535_at	REPIN1	1428850_x_at	CD99
1422119_at	RAB5B	1437536_at	FKRP	1430631_at	PPM1F	1452413_at	C230081A13RIK
1431676_x_at	GTF2I	1453556_x_at	CD99	1450500_at	UHMK1	1454106_a_at	CXXC1
1446652_at	REPS2	1457351_at	TAF2	1454106_a_at	CXXC1	1451795_at	TOM1L2
1420833_at	VAMP2	1443991_at	DOCK1	1460100_at	LBXCOR1	1459385_at	CAMSAP1L1
1458636_at	2610206B13RIK	1420881_at	NSD1	1453751_at	DHX38	1425227_a_at	ATP6V0A1
1444298_at	A130090K04RIK	1447569_at	1700081L11RIK	1420670_at	ARNT2	1422044_at	NDST1
1444141_at	SNX13	1450747_at	KEAP1	1415988_at	HDLBP	1429517_at	ZFYVE20
1431213_a_at	1300007C21RIK	1425426_a_at	MEF2A	1431021_at	CYB561D1	1453751_at	DHX38
1452185_at	IPO8	1440318_at	WDR35	1453556_x_at	CD99	1447945_at	MAF
1426191_a_at	BCL2L1	1431646_a_at	STX6	1443597_at	CRSP2	1423661_s_at	CTDSP2
1421060_at	MLLT1	1426458_at	SLMAP	1421146_at	RAPGEF1	1430275_a_at	AQR
1438686_at	EIF4G1	1420927_at	ST6GAL1	1429607_at	TRAK2	1427967_at	SRGAP2
1425227_a_at	ATP6V0A1	1451981_at	C19ORF20	1421053_at	KIF1A	1443991_at	DOCK1
1444511_at	SLC41A1	1450100_a_at	TCERG1	1438253_at	SSH1	1453946_a_at	SDCCAG8
1438043_at	EIF2C2	1452684_at	AKT1S1	1429517_at	ZFYVE20	1426975_at	4632413K17RIK
1447569_at	1700081L11RIK	1454184_a_at	IKBKB	1450161_at	IKBKG	1441309_at	ADAMTS10
1442142_at	2700050L05RIK	1456486_at	ZFP574	1422281_at	SSTR4	1422331_at	POU3F3
1415988_at	HDLBP	1426191_a_at	BCL2L1	1422136_at	UHMK1	1417613_at	IER5
1422314_at	CLCN6	1416348_at	MEN1	1416348_at	MEN1	1428123_at	2610528K11RIK
1421239_at	IL6ST	1430971_a_at	AQR	1425227_a_at	ATP6V0A1	1422107_at	2410066E13RIK
1457351_at	TAF2	1421239_at	IL6ST	1439359_x_at	NRXN1	1425426_a_at	MEF2A
1424486_a_at	TXNRD1	1430615_at	4921517B04RIK	1444511_at	SLC41A1	1443916_at	2900026A02RIK
1430144_at	5830411G16RIK	1450382_at	NF2	1428850_x_at	CD99	1446652_at	REPS2
1429607_at	TRAK2	1431676_x_at	GTF2I	1446652_at	REPS2	1428690_at	TYSND1
1430829_s_at	FTO	1458636_at	2610206B13RIK	1456255_at	AI314180	1452185_at	IPO8
1417502_at	TSPAN7	1421413_a_at	PDLIM5	1421413_a_at	PDLIM5	1439359_x_at	NRXN1
1436952_at	KLF9	1438085_at	A230048G03RIK	1425741_at	SRGAP3	1425741_at	SRGAP3
1421413_a_at	PDLIM5	1423528_at	BCAS3	1417613_at	IER5	1421949_a_at	2610507L03RIK
1420670_at	ARNT2	1430987_s_at	WBP11	1439776_at	OGFOD1	1444357_at	AKAP6
1419092_a_at	SLK	1453946_a_at	SDCCAG8	1417198_at	WWC2	1420833_at	VAMP2
1438085_at	A230048G03RIK	1454250_at	SEC15L2	1431676_x_at	GTF2I	1443597_at	CRSP2
1421216_a_at	IDS	1431675_a_at	GTF2I	1438085_at	A230048G03RIK	1437452_x_at	VDAC1
1439076_at	DHX29	1425587_a_at	PTPRJ	1430514_a_at	CD99	1440288_at	PTCHD2

1415843_at	GBL	1429607_at	TRAK2	1419092_a_at	SLK	1422314_at	CLCN6
1450528_at	B3GALT5	1426055_a_at	PIGQ	1415843_at	GBL	1440437_at	HERC1
1423528_at	BCAS3	1440844_at	TOB1	1421518_at	KCNS1	1429566_a_at	HIPK2
1449129_a_at	KCNIP3	1415843_at	GBL	1420893_a_at	TGFBR1	1422119_at	RAB5B
1440437_at	HERC1	1426538_a_at	TP53	1425426_a_at	MEF2A	1431676_x_at	GTF2I
1429517_at	ZFYVE20	1430976_a_at	MRPL9	1439076_at	DHX29	1438860_a_at	SLC44A2
1421339_at	EXTL3	1422044_at	NDST1	1421298_a_at	HIPK1	1440844_at	TOB1
1416204_at	GPD1	1440437_at	HERC1	1419682_a_at	TRP53RK	1423528_at	BCAS3
1425535_at	REPIN1	1430144_at	5830411G16RIK	1453946_a_at	SDCCAG8	1430784_a_at	4932417H02RIK
1458411_at	KNDC1	1424486_a_at	TXNRD1	1435918_at	BC055107	1420881_at	NSD1
1437648_at	PCYT1B	1436952_at	KLF9	1437648_at	PCYT1B	1415988_at	HDLBP
1419679_at	LATS2	1431385_a_at	MBTPS1	1434466_at	ATCAY	1421339_at	EXTL3
1426975_at	4632413K17RIK	1420923_at	USP9X	1417548_at	SART3	1450528_at	B3GALT5
1426458_at	SLMAP	1416515_at	FSCN1	1452185_at	IPO8	1422132_at	MTHFR
1422136_at	UHMK1	1438686_at	EIF4G1	1429566_a_at	HIPK2	1437536_at	FKRP
1421298_a_at	HIPK1	1452185_at	IPO8	1458410_at	GARNL1	1421181_at	NPTXR
1434467_at	ATCAY	1452057_at	ACTR1B	1419372_at	GOSR2	1450100_a_at	TCERG1
1420954_a_at	ADD1	1453114_at	NOL9	1459430_at	GPR158	1426191_a_at	BCL2L1
1434885_at	SPTY2D1	1439076_at	DHX29	1434467_at	ATCAY	1436952_at	KLF9
1448216_at	SYNGR3	1430021_a_at	UBLE1A	1421232_at	PLXNA1	1425587_a_at	PTPRJ
1454250_at	SEC15L2	1420670_at	ARNT2	1435944_s_at	CENPB	1450500_at	UHMK1
1434466_at	ATCAY	1431828_a_at	SYNJ2	1422968_at	IHPK1	1439776_at	OGFOD1
1437536_at	FKRP	1419092_a_at	SLK	1428123_at	2610528K11RIK	1455764_at	B930007L02RIK
1443991_at	DOCK1	1417548_at	SART3	1458411_at	KNDC1	1420968_at	BTBD14B
1456486_at	ZFP574	1425855_a_at	CRK	1442142_at	2700050L05RIK	1436083_at	LRP3
1459639_at	BRSK2	1422314_at	CLCN6	1443991_at	DOCK1	1438253_at	SSH1
1440844_at	TOB1	1439037_at	DDX17	1447369_at	1190005F20RIK	1422281_at	SSTR4
1425693_at	BRAF	1451795_at	TOM1L2	1441309_at	ADAMTS10	1450161_at	IKBKG
1417229_at	CAPN1	1447207_at	2810482G21RIK	1421239_at	IL6ST	1439076_at	DHX29
1431828_a_at	SYNJ2	1446652_at	REPS2	1425693_at	BRAF	1430615_at	4921517B04RIK
1431932_s_at	TRIM44	1422119_at	RAB5B	1425204_s_at	DDX19A /// DDX19B	1451981_at	C19ORF20
1416515_at	FSCN1	1434466_at	ATCAY	1422314_at	CLCN6	1425535_at	REPIN1
1429566_a_at	HIPK2	1434885_at	SPTY2D1	1431932_s_at	TRIM44	1416515_at	FSCN1
1438425_at	GTF3C1	1444511_at	SLC41A1	1444357_at	AKAP6	1456676_a_at	PFKFB3
1453946_a_at	SDCCAG8	1436306_at	SAPS1	1432196_a_at	DSCAML1	1432196_a_at	DSCAML1
1448207_at	LASP1	1419372_at	GOSR2	1436952_at	KLF9	1421146_at	RAPGEF1
1443597_at	CRSP2	1421339_at	EXTL3	1438425_at	GTF3C1	1417882_at	SLC39A3
1430976_a_at	MRPL9	1425170_a_at	ADAM15	1421949_a_at	2610507L03RIK	1450041_a_at	TUB
1458410_at	GARNL1	1421789_s_at	ARF3	1452747_at	ATP13A2	1431116_at	C030003D03RIK
1437768_at	ANKIB1	1450161_at	IKBKG	1434885_at	SPTY2D1	1453114_at	NOL9
1450747_at	KEAP1	1421900_at	EIF2AK1	1459385_at	CAMSAP1L1	1423402_at	CREB1
1420744_at	CHRNB2	1418795_at	CDS2	1416516_at	FSCN1	1418938_at	DIO2
1453995_a_at	HTF9C	1438860_a_at	SLC44A2			1431097_at	GARNL1
1459317_at	ANK2	1425693_at	BRAF			1450402_at	PPARBP
1421053_at	KIF1A	1438043_at	EIF2C2			1425154_a_at	CSF1
1421146_at	RAPGEF1	1435918_at	BC055107			1454184_a_at	IKBKB
1450100_a_at	TCERG1	1452467_at	MMAB			1425855_a_at	CRK
1459430_at	GPR158	1429517_at	ZFYVE20			1420923_at	USP9X
1420927_at	ST6GAL1	1419022_a_at	ENO1			1416851_at	ST13
1447945_at	MAF	1437648_at	PCYT1B			1419679_at	LATS2
1451981_at	C19ORF20	1450022_at	GTPBP1			1457351_at	TAF2
1456676_a_at	PFKFB3	1419679_at	LATS2			1458443_at	CRTC1
1439776_at	OGFOD1	1424178_at	TMEM38A			1439910_a_at	TRADD
1428123_at	2610528K11RIK	1432638_at	BAG5			1425097_a_at	ZFP106
1416348_at	MEN1	1452419_at	HEATR1			1420749_a_at	POU6F1
1450382_at	NF2	1456490_at	CDK2AP2			1419372_at	GOSR2
1422984_at	CYLN2	1421298_a_at	HIPK1			1425337_at	SLC12A5
1452057_at	ACTR1B	1448207_at	LASP1			1420893_a_at	TGFBR1
1445241_at	RAB11FIP4	1428993_at	1110017I16RIK			1459832_s_at	AP1M1
1430987_s_at	WBP11	1421216_a_at	IDS			1452419_at	HEATR1

1419682_a_at	TRP53RK
1438253_at	SSH1
1420749_a_at	POU6F1
1453751_at	DHX38
1440318_at	WDR35
1456024_at	GTF3C1
1426699_at	AU040320
1453114_at	NOL9
1424178_at	TMEM38A
1419372_at	GOSR2
1439037_at	DDX17
1431021_at	CYB561D1
1421232_at	PLXNA1
1425855_a_at	CRK
1425170_a_at	ADAM15
1430514_a_at	CD99
1421900_at	EIF2AK1
1444357_at	AKAP6
1428850_x_at	CD99
1452747_at	ATP13A2
1450500_at	UHMK1
1417548_at	SART3
1426055_a_at	PIGQ
1426795_at	PTPRS
1428690_at	TYSND1
1438860_a_at	SLC44A2
1430021_a_at	UBLE1A
1429362_a_at	SF3B2
1450022_at	GTPBP1
1418795_at	CDS2
1417678_at	MMP24
1426538_a_at	TP53
1421480_a_at	ADARB1
1425204_s_at	DDX19A /// DDX19B
1453607_at	MFAP3L
1459385_at	CAMSAP1L1
1435944_s_at	CENPB
1436306_at	SAPS1
1419022_a_at	ENO1
1452684_at	AKT1S1
1431400_a_at	GAS7
1420968_at	BTBD14B
1426125_a_at	CASP9
1431646_a_at	STX6
1427117_at	MTMR3
1454184_a_at	IKBKB
1429820_at	RUTBC2
1445189_at	GPATC2
AFFX-PyruCarbMur/L09192_5_at	PCX
1453730_at	SAMD8
1418938_at	DIO2
1440480_at	AB182283
1425865_a_at	LIG3
1453556_x_at	CD99
1431663_a_at	CNTFR
1427652_x_at	SYNJ2
1439359_x_at	NRXN1
1450651_at	MYO10
1456595_x_at	GGH
1422107_at	2410066E13RIK

1445189_at	GPATC2
1452174_at	SREBF2
1458410_at	GARNL1
1445241_at	RAB11FIP4
1434467_at	ATCAY
1420749_a_at	POU6F1
1418938_at	DIO2
1455873_a_at	VPS18
1450556_at	SPNB1
1421949_a_at	2610507L03RIK
1425204_s_at	DDX19A /// DDX19B
1450651_at	MYO10
1428690_at	TYSND1
1431021_at	CYB561D1
1455764_at	B930007L02RIK
1431097_at	GARNL1
1420833_at	VAMP2
1421232_at	PLXNA1
1460314_s_at	HIST2H3C1 /// HIST2H
1416521_at	SEPW1
1421053_at	KIF1A
1425535_at	REPIN1
1453607_at	MFAP3L
1438425_at	GTF3C1
1422331_at	POU3F3
1424787_a_at	NRF1
1425865_a_at	LIG3
1444298_at	A130090K04RIK
1419682_a_at	TRP53RK
1417198_at	WWC2
1421060_at	MLLT1
1421518_at	KCNS1
1420373_at	FOXJ2
1416204_at	GPD1
1421146_at	RAPGEF1
1452413_at	C230081A13RIK
1451680_at	SRXN1

1417229_at	CAPN1
1429112_at	TLN2

1434902_at	RNF157
1421994_a_at	HS1BP3
1450515_at	KCNJ11
AFFX-PyruCarbMur/L09192_MA_at	PCX
1425817_a_at	SLC8A1
1452413_at	C230081A13RIK
1456304_at	GM996
1450161_at	IKBKG
1460314_s_at	HIST2H3C1 /// HIST2H
1420373_at	FOXJ2
1452174_at	SREBF2
1426115_a_at	KCNJ9
1451849_a_at	LMNB2
1422968_at	IHPK1
1449552_at	ZFR
1429112_at	TLN2
1438440_at	RNASEN
1421181_at	NPTXR
1417613_at	IER5
1424787_a_at	NRF1
1421032_a_at	DNAJB12
1415804_at	CX3CL1
1425097_a_at	ZFP106
1436083_at	LRP3
1427732_s_at	ABCG4
